# Comparative cranial morphology of the Late Cretaceous protostegid sea turtle *Desmatochelys lowii*

**DOI:** 10.7717/peerj.5964

**Published:** 2018-12-07

**Authors:** Irena Raselli

**Affiliations:** Departement of Geosciences, University of Fribourg, Fribourg, Switzerland

**Keywords:** Protostegidae, Chelonioidea, Comparative morphology, Cranial anatomy, Testudines, *Desmatochelys lowii*, Late Cretaceous, Cryptodira, Sea turtle evolution

## Abstract

**Background:**

The phylogenetic placement of Cretaceous marine turtles, especially Protostegidae, is still under debate among paleontologists. Whereas protostegids were traditionally thought to be situated within the clade of recent marine turtles (Chelonioidea), some recent morphological and molecular studies suggest placement along the stem of Cryptodira. The main reason why the evolution of marine turtles is still poorly understood, is in part due to a lack of insights into the cranial anatomy of protostegids. However, a general availability of high-quality fossil material, combined with modern analysis techniques, such as X-ray microtomography, provide ample opportunity to improve this situation. The scope of this study is to help resolve its phylogenetic relationships by providing a detailed description of the external and internal cranial morphology of the extinct protostegid sea turtle *Desmatochelys lowii* Williston, 1894.

**Material and Methods:**

This study is based on the well-preserved holotype of *Desmatochelys lowii* from the Late Cretaceous (middle Cenomanian to early Turonian) Greenhorn Limestone of Jefferson County, Nebraska. The skulls of two recent marine turtles, *Eretmochelys imbricata* (Linnaeus, 1766) (Cheloniidae) and *Dermochelys coriacea* Lydekker, 1889 (Dermochelyidae), as well as the snapping turtle *Chelydra serpentina* (Linnaeus, 1758) (Chelydridae) provide a comparative basis. All skulls were scanned using regular or micro CT scanners and the scans were then processed with the software program Amira to create 3D isosurface models. In total, 81 bones are virtually isolated, figured, and described, including the nature of their contacts. The novel bone contact data is compiled and utilized in a preliminary phenetic study. In addition, an update phylogenetic analysis is conduced that utilizes newly obtained anatomical insights.

**Results:**

The detailed examination of the morphology of the herein used specimens allowed to explore some features of the skull, to refine the scoring of *Desmatochelys lowii* in the recent global matrix of turtles, and develop five new characters. The alleged pineal foramen in the type skull of *Desmatochelys lowii* is shown to be the result of damage. Instead, it appears that the pineal gland only approached the skull surface, as it is in *Dermochelys coriacea*. Whereas the parasphenoid in confirmed to be absent in hard-shelled sea turtles, ist possible presence in *Desmatochelys lowii* is unclear. The results of the phenetic study show that *Desmatochelys lowii* is least similar to the other examined taxa in regards to the nature of its bone contacts, and therefore suggests a placement outside Americhelydia for this protostegid sea turtle. The phylogenetic study results in a placement of Protostegidae along the stem of Chelonioidea, which is a novel position for the group.

## Introduction

All recent marine turtles (i.e., turtles that permanently live in fully marine environments) are currently accepted to form a monophyletic group, Chelonioidea Baur, 1893, that consists of two clades: the hard-shelled Cheloniidae Bonaparte, 1832 and the leathery-shelled Dermochelyidae Lydekker, 1889 (Crawford et al., 2015; Pereira et al., 2017). Whereas the former group consists of six species distributed across all tropical to warm temperate oceans, the latter group is represented only by one species with a global distribution, the leatherback turtle *Dermochelys coriacea* (Turtle Taxonomy Working Group (TTWG), 2017). The ancestry of marine turtles, however, is controversial for paleontologists, because relationships are unclear between extant marine turtles and various groups of fossils turtles adapted to marine environments (see Cadena & Parham, 2015, for recent summary). The following groups of fossil turtles are currently thought to be marine: the Late Jurassic Eurysternidae Dollo, 1886, Plesiochelyidae Baur, 1888, and Thalassemydidae Zittel, 1889 (Anquetin, Püntener & Joyce, 2017), which were recently grouped as Thalassochelydia Anquetin, Püntener & Joyce, 2017, the Cretaceous Protostegidae Cope, 1872 (Cadena & Parham, 2015) and certainly paraphyletic or polyphyletic toxochelyid-grade stem chelonioids (Parham & Pyenson, 2010), the Early Cretaceous to Paleogene Bothremydidae Baur, 1891 (Gaffney, Tong & Meylan, 2006) and Sandownidae Tong & Meylan, 2012 (Cadena, 2015), and, finally, the Tertiary Stereogenyina Gaffney et al., 2011. Whereas it is apparent that the two clades of marine pleurodires, Bothremydidae and Stereogenyina, have no immediate relationships with their extant cryptodiran cousins, all other fossil groups have at one point or the other been suggested to be ancestral or related to extant marine turtles.

Recent discussions have focused on the phylogenetic position of protostegids. On the one hand, some studies suggest that protostegids are situated within crown Chelonioidea (Gaffney & Meylan, 1988; Hirayama, 1998; Cadena & Parham, 2015), while others place them outside of crown Cryptodira (Joyce, 2007; Anquetin, 2012; Rabi et al., 2013). However, it has to be mentioned, that these studies base on the same dataset for sea turtles and their sampling of Protostegidae is limited to *Santanachelys gaffneyi* Hirayama, 1998. The latter hypothesis is concordant with molecular calibration analyses, as these suggest a divergence of crown Chelonioidea near the K/T boundary event (Joyce et al., 2013). One of the primary reasons why the interrelationships of marine turtles remain unresolved is because only few specimens from the Mesozoic have been described in detail, even though much interesting material is available for study and new methods available to access anatomical information. As a consequence, modern studies using cladistic methodologies still often rely on outdated literature that often provides simplified or incomplete illustrations of specimens. A good example is the holotype of the Late Cretaceous protostegid *Desmatochelys lowii* Williston, 1894. The specimen was collected in 1893 near Fairbury, Jefferson County, Nebraska from the Late Cretaceous Greenhorn Limestone (formerly Benton Cretaceous) in sediments that had once been deposited in the Western Interior Seaway. The “Benton Cretaceous” is now regionally classified as the Greenhorn Limestone, which is middle Cenomanian to lower Turonian in age (Hattin, 1975). Even though the specimen includes the best-preserved skull of *Desmatochelys lowii* in particular, but also one of the best preserved protostegid skulls in general, it was only superficially described by Williston (1894), Zangerl & Sloan (1960), and Elliot, Irby & Hutchison (1997).

The purpose of this study is to document in detail the cranial morphology of the holotype of *Desmatochelys lowii*. As the detailed cranial anatomy is not yet available for any other species of protostegid, the skull is here compared on a bone-by-bone basis to the extant marine turtles *Eretmochelys imbricata* (Cheloniidae) and *Dermochelys coriacea* (Dermochelyidae), as well as the freshwater snapping turtle *Chelydra serpentina* (Chelydridae). All specimens were CT scanned and their bones 3D visualized to provide the greatest amount of possible new insights into their cranial morphology. The resulting data was then used to update the scoring of *Desmatochelys lowii* in the latest available global matrix of marine turtle relationships (Cadena & Parham, 2015). In addition, an exploratory phenetic study is conducted that seeks phylogenetic information from bone contacts.

## Materials and Methods

### Material

The osteology of the fossil marine turtle *Desmatochelys lowii* is herein described in detail based on CT scans. For comparison, the study includes scans of two recent marine turtles, *E. imbricata* and *Dermochelys coriacea*, and *C. serpentina* as the “outgroup.” The left side of each skull is illustrated and serves for the description of the morphology of each isolated bone. In cases of asymmetry or partial damage of the skull, the morphological structures on other side of the skull have been scrutinized and included into the description.

*Desmatochelys lowii* Williston, 1894

The description of this species is based KUVP 1200, which is the holotype *Desmatochelys lowii*, currently housed in the collection of the University of Kansas in Lawrence, Kansas, USA. The skull is 21.5 cm long and has a maximum width of 14 cm. It is posteroventrally crushed and its basicranium is pierced by a hole of 1.5 cm in diameter that was drilled into the skull for mounting following its initial description. Although this specimen is lightly damaged and internally filled with matrix, it is the best-preserved skull of this species known to date (Everhart & Pearson, 2009; Elliot, Irby & Hutchison, 1997).

*Eretmochelys imbricata* (Linnaeus, 1766)

The species is represented by NMB C.2417, which is housed in the collection of the Naturhistorisches Museum Basel, Switzerland. The skull is 12.5 cm long and has a maximum width of 6.5 cm. The specimen lacks locality information.

*Dermochelys coriacea* (Vandellii, 1761)

The description of this species is based on SMF 62797 of the Department of Herpetology at the Senckenberg Naturmuseum Frankfurt, Germany. The skull is 25 cm long and has a maximum width of 21 cm. It was collected in 1966 near the Hebrides in the North Sea by the Institut für Meeresforschung in Bremerhaven and donated to the Senckenberg in 1967.

*Chelydra serpentina* (Linnaeus, 1758)

This species is based on UFR VP1, which is currently housed at the Department of Geosciences at the University in Fribourg in Switzerland. The specimen lacks locality data, but likely originates from the USA. The skull is 12 cm long and has a maximal width of nine cm.

### Digital data generation

#### X-ray computed tomography and reconstruction

The four selected specimens were scanned at three different CT scanning facilities, mostly due to logistic demands and size constraints. The raw data were afterward converted into slices using in-house software associated with the CT scanners. The most important scanning and reconstruction parameters are provided in the Tables 1 and 2. The original CT scan images are available on Morphobank (see link in Appendix).

**Table 1:** Scan settings for the specimens used herein.

Specimen	Scanner	Institution	Voltage (kV)	Current (μA)	Voxel size (μm)	Filter
*Desmatochelys lowii*	Phoenix v¦tome¦x	University of Chicago, Department of Organismal Biology and Anatomy	210	190	79.8	Cu 0.15 mm
	μCT					Sn
						0.50 mm
*Eretmochelys imbricata*	Bruker SkyScan	University of Fribourg, Department of Geosciences	125	63	43.0	0.50 Al mm
	μCT					
*Dermochelys coriacea*	Siemens	University of Bern, Institute of Forensic Medicine	120	210	98.6	–
	Medical					
	CT					
*Chelydra serpentina*	Bruker SkyScan	University of Fribourg, Department of Geosciences	80	550	35.0	Ti
	μCT					0.50 mm
						Al
						0.125 mm

**Table 2:** Settings used in the conversion of scan data to slice data.

Specimen	Software	Smoothing	Ring artifact correction	Beam hardening correction
*Desmatochelys lowii*	–	–	–	–
*Eretmochelys imbricata*	NRecon	1	0	70%
*Dermochelys coriacea*	–	–	–	–
*Chelydra serpentina*	NRecon	0	16	13%

#### Segmentation and generation of 3D models

**Figure 1:**
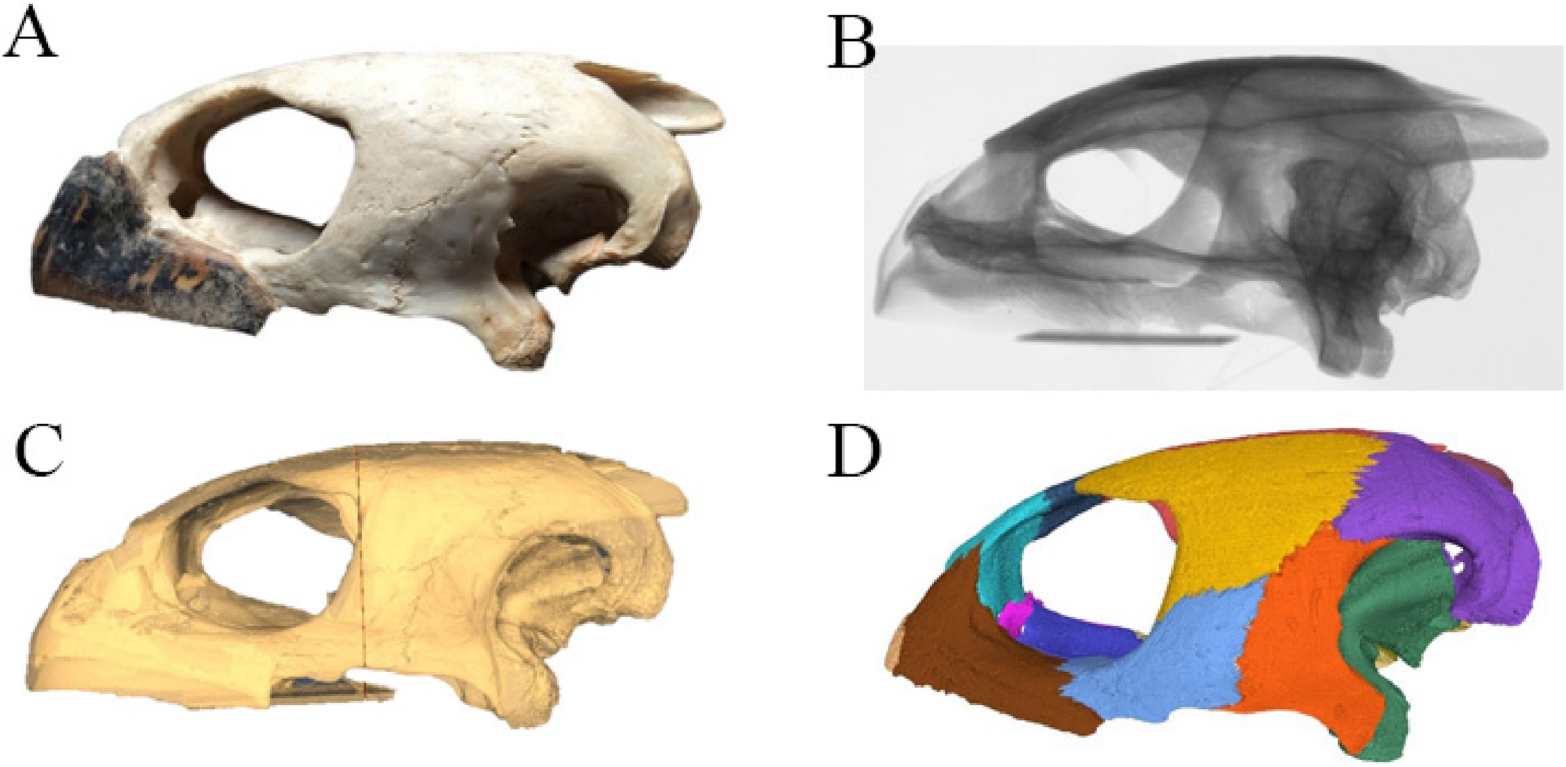
The skull of *Eretmochelys imbricata* in four types of digital representation (A–D).

Virtual isolation of the skull bones was performed with the software Amira (version 6.0.0) (Fig. 1 and Table S2.1). Segmentation was implemented from all perspectives by hand, mostly with the brush tool and, occasionally, with the magic wand, drawing the limits by hand. Each bone was labeled using a different color and the same color was applied to homologous structures in different specimens. Depending on the complexity of bone morphology, every third or fifth slice was labeled, followed by interpolation of the marked areas. The segmentation process was performed applying the masking option for the pixels belonging to the gray scale rage of the bone. The 3D models used herein were generated in Amira as well. The 3D models of the skulls were left unaltered with exception of that of *Dermochelys coriacea*, which was smoothed using a factor of 2 to cache stepping caused by disproportionally large voxel size. The images provided in the text were generated using the screenshot function of Amira.

### Bone contact analysis

**Figure 2:**
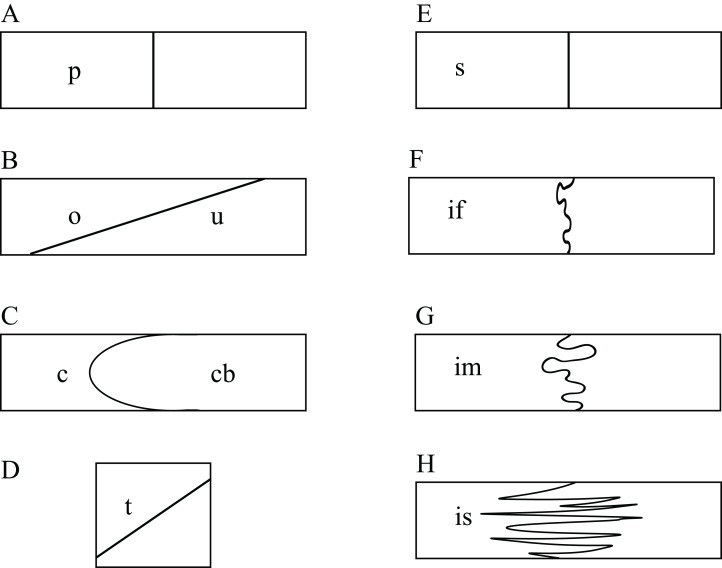
Sketches illustrating the categories of observed spatial relations between bones (A–D) and the depth of sutures (E–H). The spatial relations between bones can be classified into: (A) p, parallel; (B) o, overlapping or u, underlying; (C) c, clasping or cb: clasped by; and (D) t, vertically transverse. The depth of suture can range from (E) s, smooth; (F) if, faintly interfingering; (G) im, moderately interfingering to; and (H) is, strongly interfingering.

**Figure 3:**
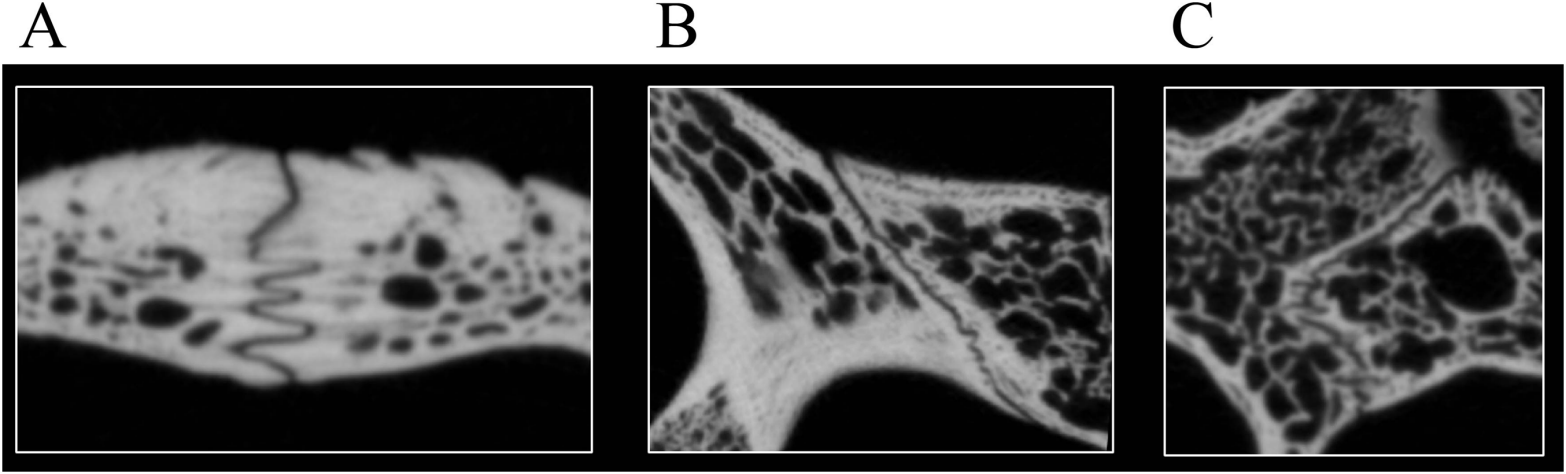
Examples of bone contacts based on cross section images in *Eretmochelys imbricata*. (A) Parallel and moderately interfingering; (B) underlying/overlapping and faintly interfingering; and (C) clasping and faintly interfingering. The width of each image is about one cm.

The use of CT data allows studying the detailed nature at which bones contacts each other within a skull, which is a novel source of data that may prove valuable in the future in phylogenetic or biomechanical analyses. The following classification was developed to provide a standardized nomenclature and to help generate data. Two different morphological aspects are addressed in this classification. On the one side, contacts can be described in regards to the spatial relationships of the involved structures. Bones can contact each other in a suture that stands perpendicular to their surfaces (i.e., “parallel”), they can broadly overlap each other (i.e., “overlapping” or “underlying”), or one bone can clasp another (i.e., “clasping”). On the other side, the actual contact between two bones can vary in regards to the depth of the suture, ranging from blunt (i.e., “smooth”) to strongly interfingering (i.e., “faintly interfingering,” “moderately interfingering,” and “strongly interfingering”). These categories are figured in Fig. 2 and examples provided in Fig. 3.

The varying contacts that can be observed in each of the four scanned specimens were documented in the Description using the newly developed nomenclature (see below) and tabulated for analysis (Tables S3.1–S3.4). Whenever contacts vary between two bones the different kinds of contacts are listed from the anterior to posterior, also in the tables. Some bone contacts are not clear in the scanned specimen of *D. lowii* and the spatial relations of these bones are therefore determined, but not the type of suture. The contacts of the nasal were excluded from the table as this bone only occurs in *D. lowii*.

To explore if bone contact data contains a signal, a similarity matrix was calculated that expresses the percentage of bony contacts that are similar between two given species. Any difference in spatial relationships of bones is hereby considered to constitute dissimilarity. However, to be considered dissimilar, the suture depth has to be different by at least two categories. Along those lines, a faintly interfingering clasping suture is considered to be similar to an intermediately interfingering clasping suture, but not with a strongly interfingering clasping suture.

### Phylogenetic analysis

A phylogenetic analysis was performed herein to explore if the new insights gained into the anatomy of *D. lowii* have an impact on its phylogenetic placement. For this purpose, the global character/taxon matrix of Cadena & Parham (2015) was utilized, which in return is a combination of previously published matrices of marine turtle relationships (Hirayama, 1994, 1998; Kear & Lee, 2006; Parham & Pyenson, 2010; Bardet et al., 2013; Lapparent De Broin et al., 2014) and global turtle phylogenies (Joyce, 2007; Sterli, 2008; Joyce et al., 2011; Anquetin, 2012; Rabi et al., 2013; Sterli & De la Fuente, 2013; Zhou, Rabi & Joyce, 2014). The matrix was adjusted using the following modification. First, the codings were updated for 10 characters for *D. lowii* (see Results for list of changes). Second, the matrix was expanded by seven characters, of which five have previously not been used in phylogenetic analyses. The matrix was assembled in Mesquite 3.31. The final matrix consists of 154 taxa and 263 characters. The phylogenetic analysis was performed using TNT 1.1 (Goloboff, Farris & Nixon, 2008). *Odontochelys semitestacea* was defined as the out-group. Characters 7, 17, 22, 44, 49, 52, 55, 57, 59, 66, 70, 71, 76, 79, 89, 101, 106, 118, 122, 128, 135, 138, 144, 148, 171, 174, 189, 191, 211, 223, 225, 229, 244, 256, 257, 258, and 262 form morphoclines and were therefore run ordered. Following Cadena & Parham (2015), 81 taxa were deactivated and a backbone constraint tree topology was used that constrains the topology of extant turtles based on the molecular analysis of Crawford et al. (2015) (see Appendix 4). The matrix was subjected to 1,000 replicates of random addition sequences followed by a second round of tree bisection-reconnection. To eliminate “wildcard” taxa, the strict consensus tree was pruned through the “iterPCR” script form Pol & Escapa (2009). The tree was then reduced by the two “wildcard” taxa, the *Buliachelys suteri* and *Puppigerus camperi*. Finally, the consistency index and the retention index were calculated for the resulting tree, using the script “statsall” (designed by Peterson, L. Lopes).

## Description

The description of the available skulls, bases on the virtually isolated bones and includes comparison on a bone-by-bone level.

### Quality of CT data

**Figure 4:**
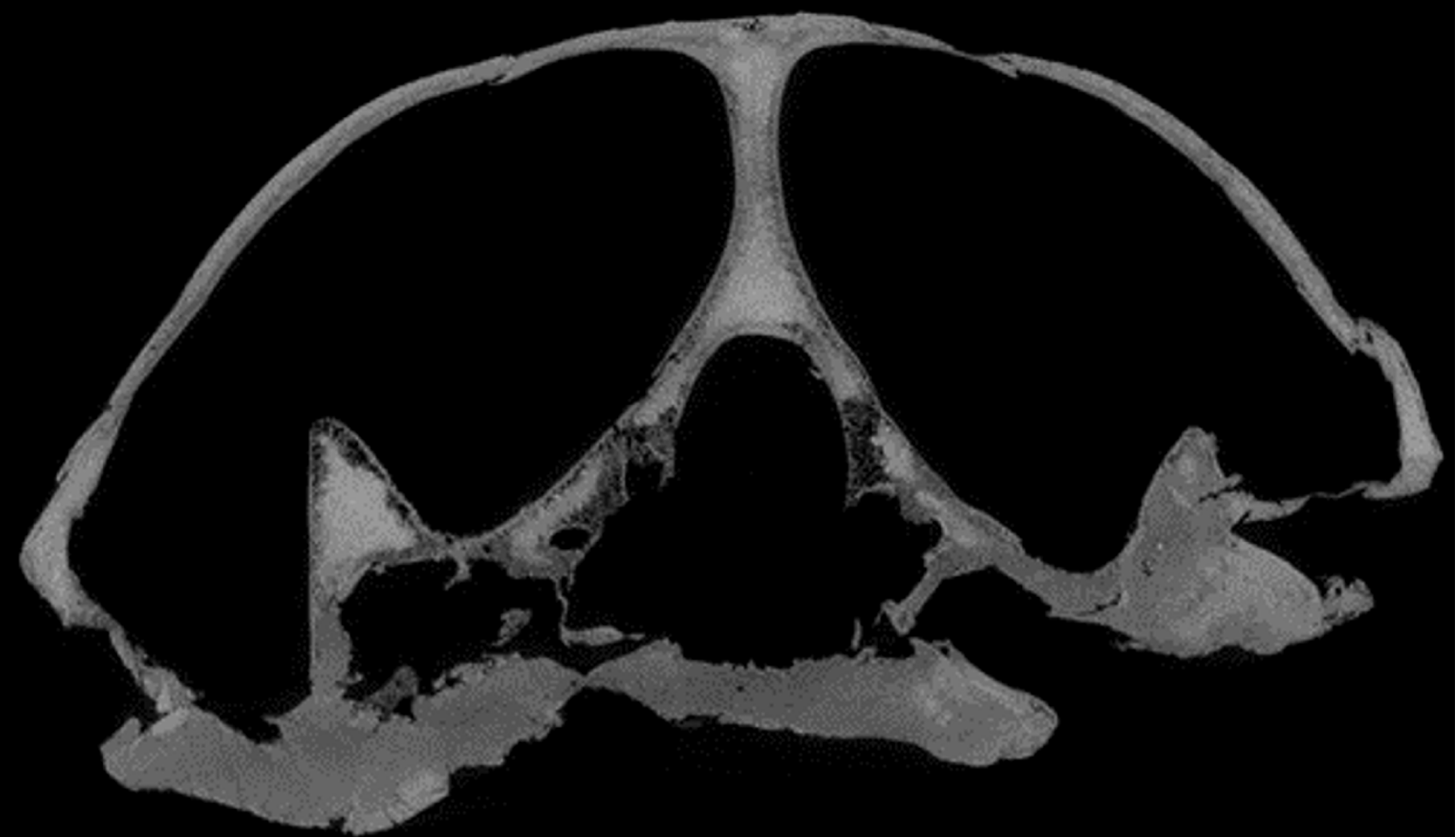
Cross section image of *D. lowii* along the coronal plane. The lower half of the skull is damaged and obscured by matrix.

*Desmatochelys lowii—*Although the relatively large skull of *D. lowii* was scanned using a X-ray microtomography (μCT) scanner and therefore has a relatively high resolution, many details are obscured by crushing, fractures, and, more importantly, a lacking contrast between matrix and bones, especially toward the back of the skull (Fig. 4 and Fig. S1.2).

*Eretmochelys imbricata—*The resolution and contrast of the CT scans of *E. imbricata* (and *C. serpentina*) were the best in this study, as they are based on recent material and were scanned using a μCT scanner (see Material and Methods).

*Dermochelys coriacea—*The CT scans of *Dermochelys coriacea* were produced using a medical CT scanner and the voxel are therefore disproportionally large relative to the skull size, which obscures the details of some structures (see Fig. S1.6).

*Chelydra serpentina—*The skull of *C. serpentina*, is characterized by a strong ornamentation on the skull roof and most contacts are tight sutured (see Figs. S1.7 and S1.8), which lead to difficulties in determining the exact limits of the bones in some parts of the skull.

### Skull shape

**Figure 5:**
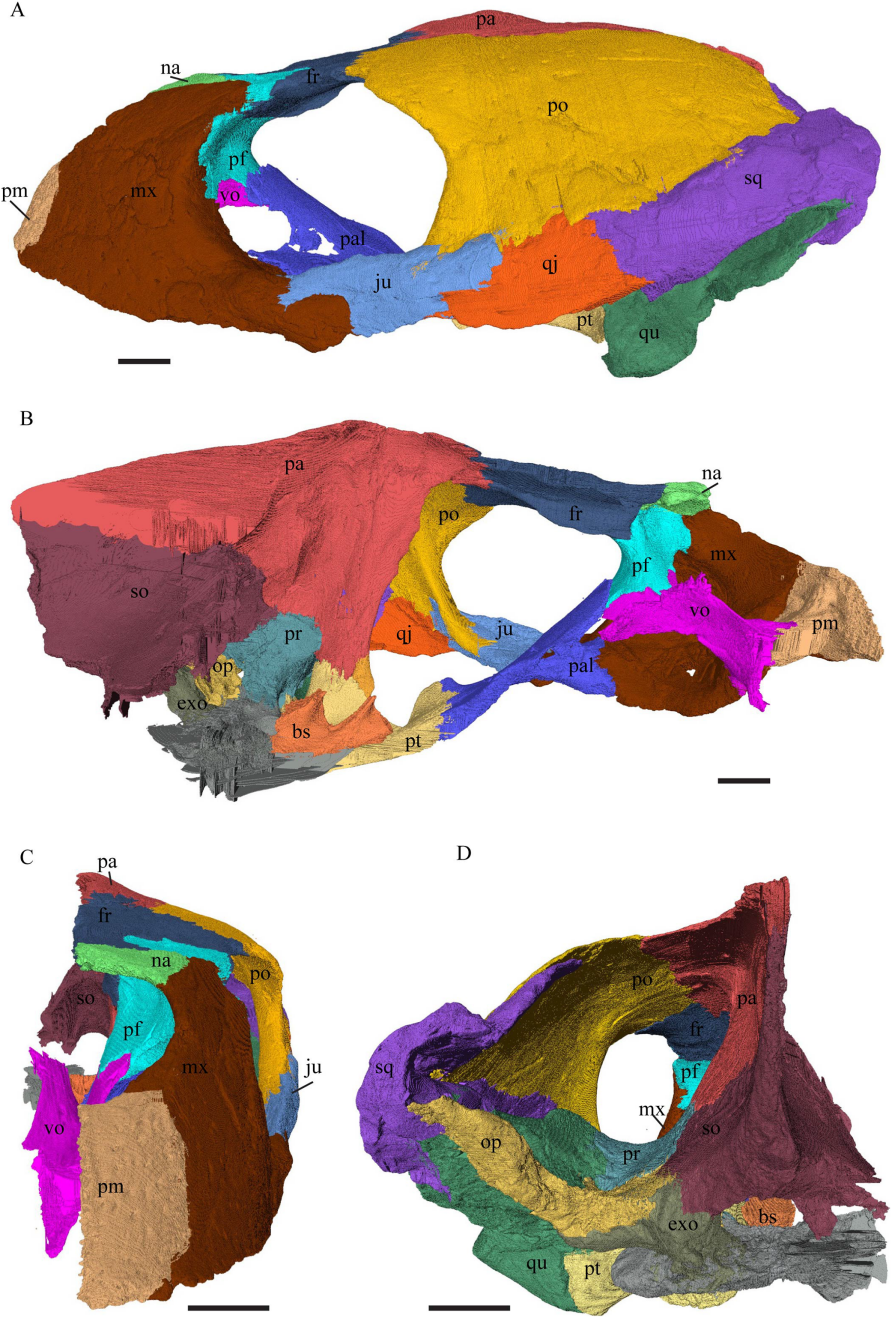
The segmented skull of *D. lowii*. (A) Lateral; (B) medial; (C) anterior and (D) posterior views.

**Figure 6:**
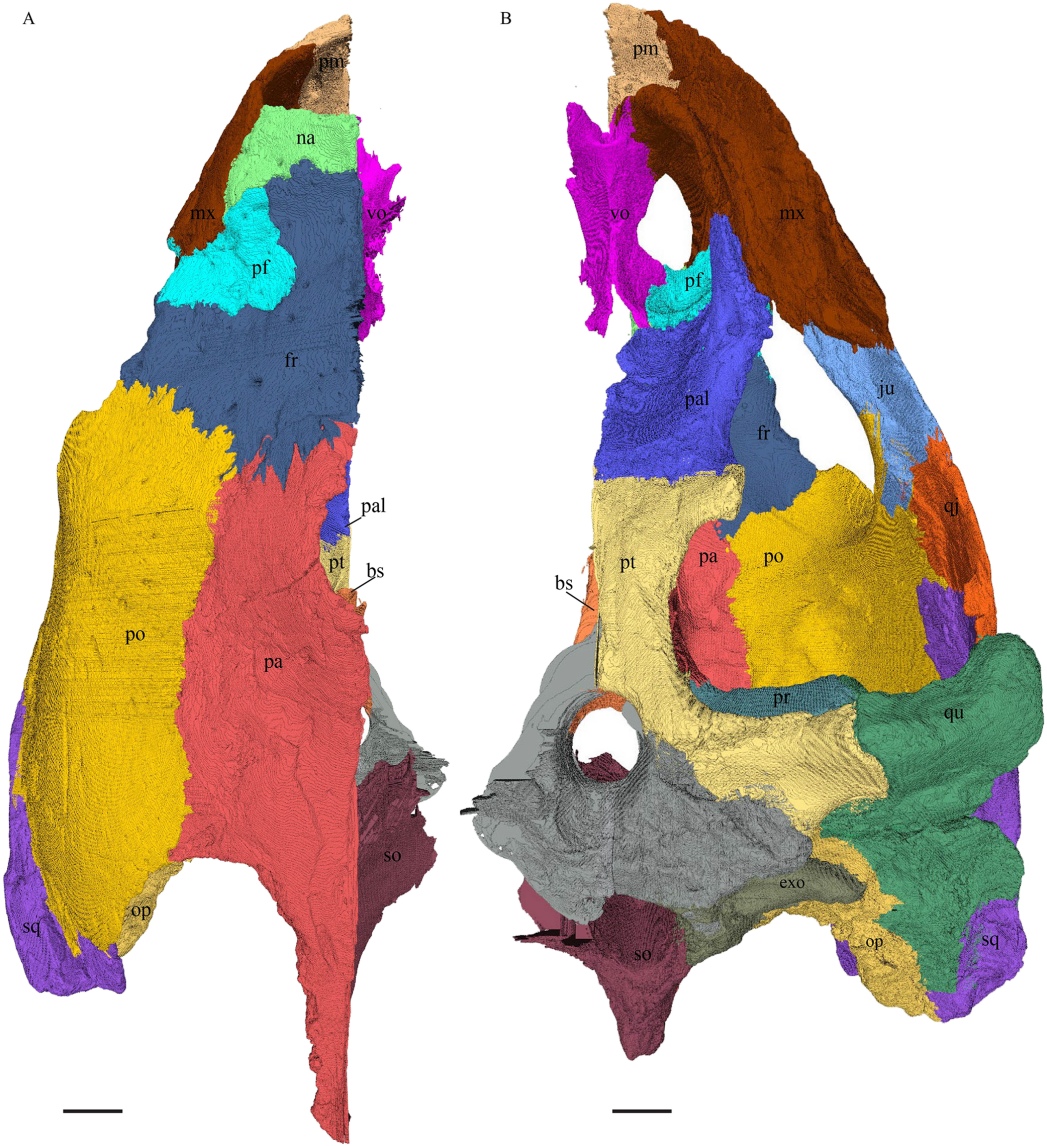
The segmented skull of *D. lowii*. (A) Dorsal and (B) ventral views. Gray area represents unknown anatomy. The bar marks 10 mm.

*Desmatochelys lowii—*The skull of *D. lowii* has a rather narrow rostrum, large, laterally facing orbits, a prominent lateral protuberance that spans from the jugal to the squamosal, and a median bulge formed by the parietals (Figs. 5 and 6). The cavum tympani is notably elongate, but the antrum postoticum is not developed. The palate is relatively narrow, but nevertheless shows well-developed lingual ridges.

**Figure 7:**
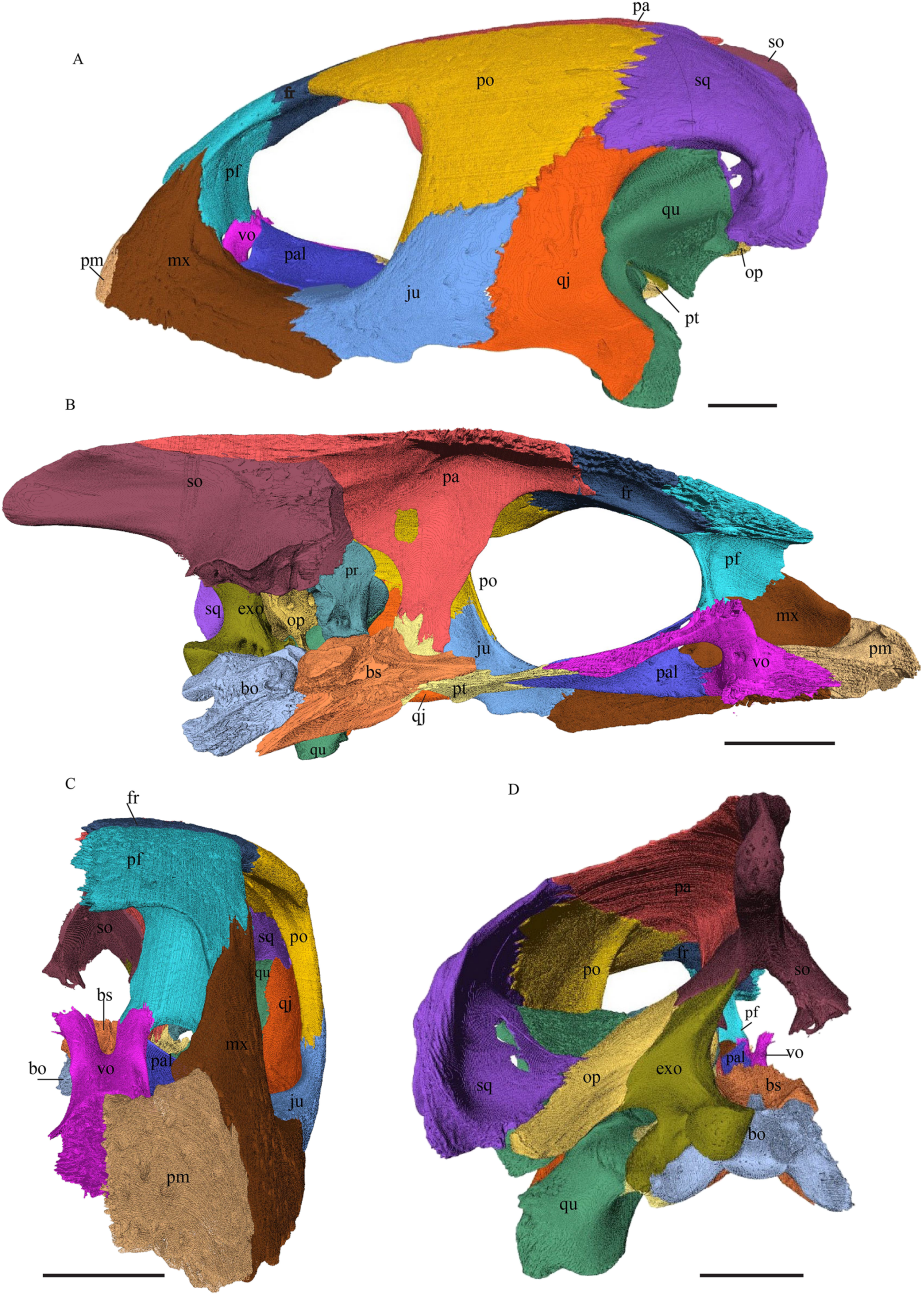
The segmented skull of *Eretmochelys imbricata*. (A) Lateral; (B) medial; (C) anterior; and (D) posterior views. The bar marks 10 mm.

**Figure 8:**
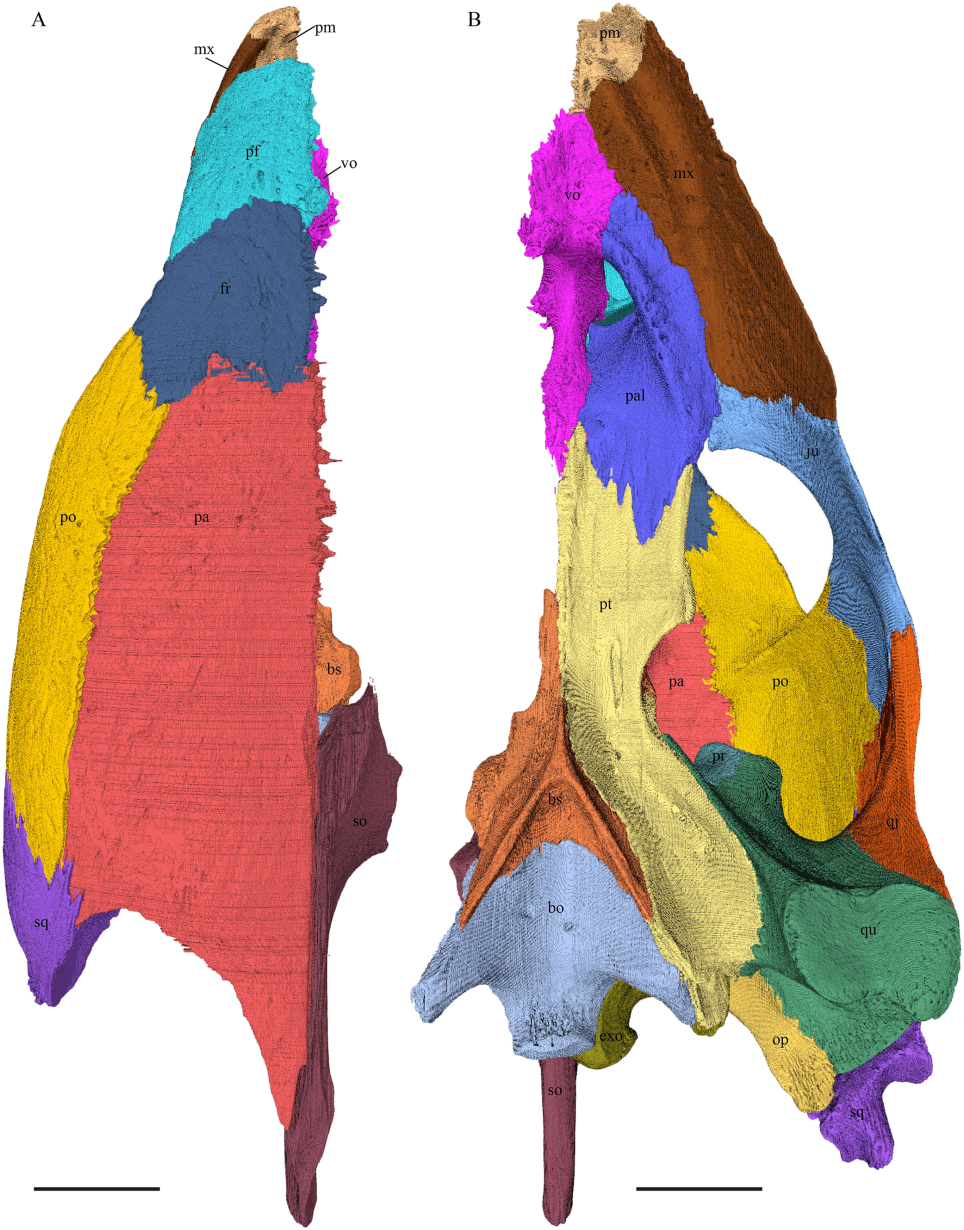
The segmented skull of *Eretmochelys imbricata*. (A) Dorsal and (B) ventral views. The bar marks 10 mm.

*Eretmochelys imbricata—*The skull of *E. imbricata* has a narrow rostrum, the orbits are large and facing laterally, and the width of the skull is rather constant (Figs. 7 and 8). The cavum tympani is rounded, but the antrum postoticum is not developed. A modest secondary palate is developed that includes low lingual ridges.

**Figure 9:**
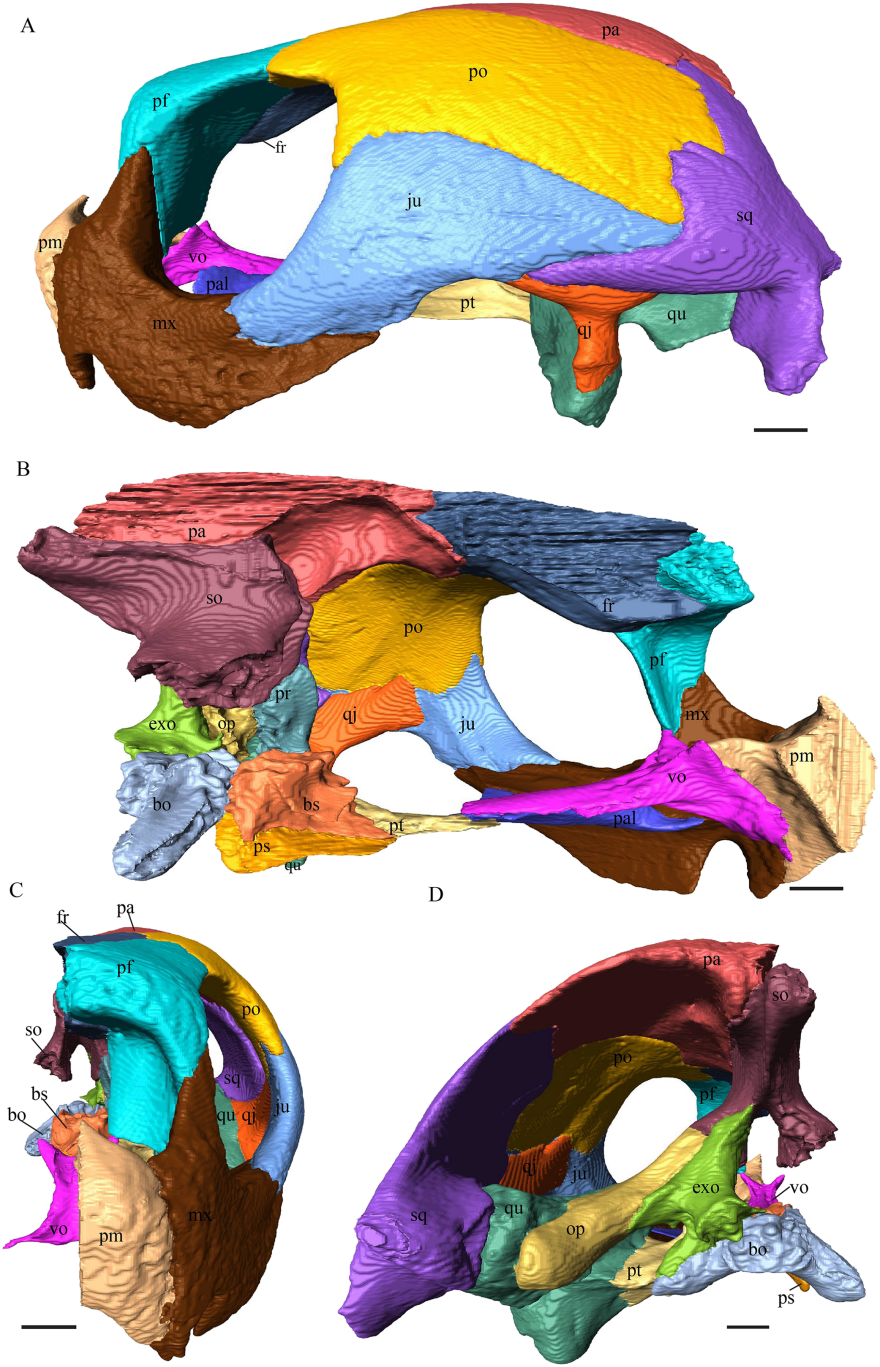
The segmented skull of *D. coriacea*. (A) Lateral; (B) medial; (C) anterior and (D) posterior views. The bar marks 10 mm.

**Figure 10:**
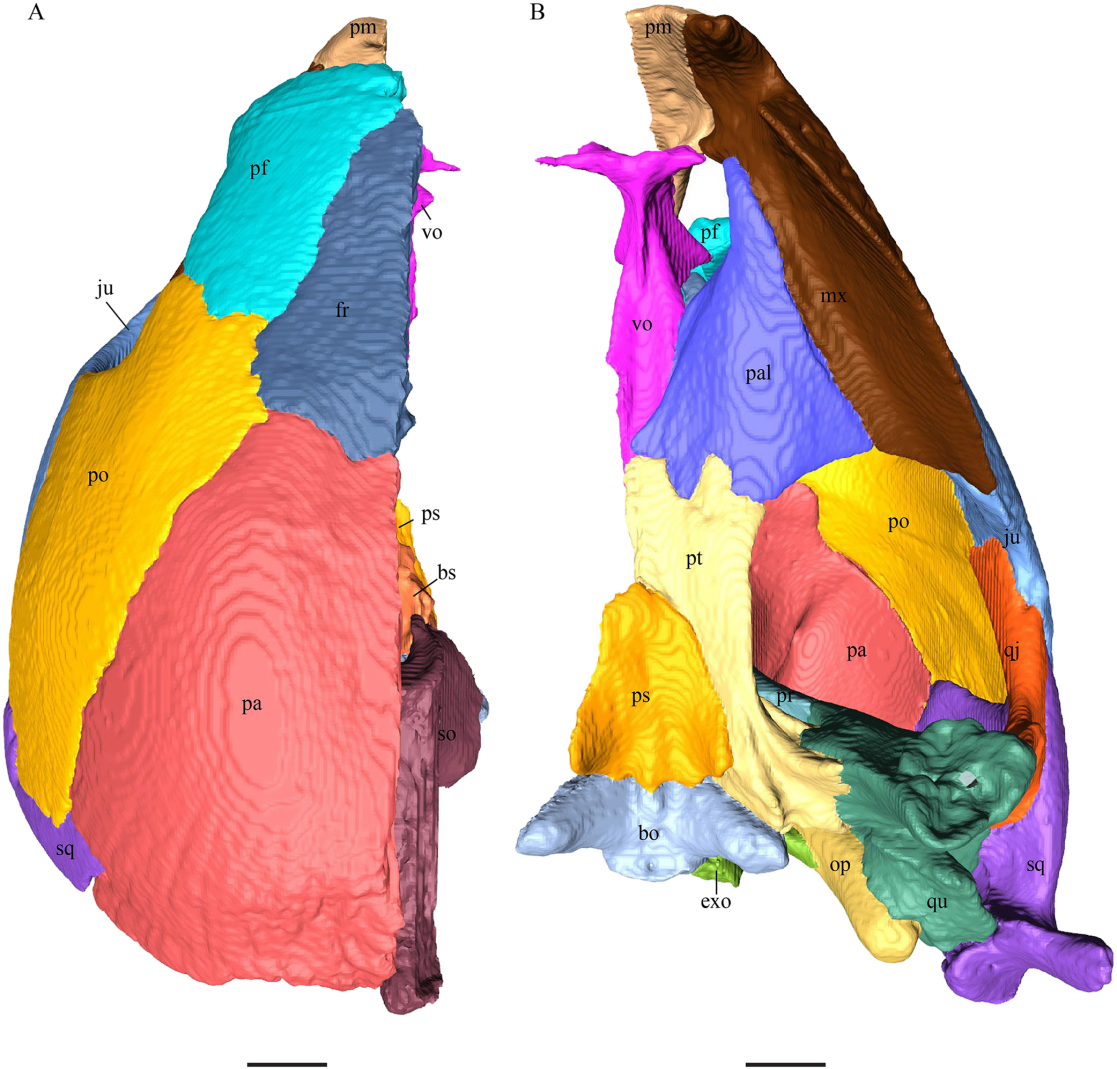
The segmented skull of *D. coriacea*. (A) Dorsal and (B) ventral views. The bar marks 10 mm.

*Dermochelys coriacea—*The skull of *Dermochelys coriacea* is rather short relative to its width and has laterally facing orbits (Figs. 9 and 10). The middle ear lacks either a well-defined cavum tympani or an antrum postoticum. The anterior part of the labial ridge is marked by a notch and tooth on each side. The crista supraoccipitalis is short. A broad median depression on the skull roof is formed by the parietals.

**Figure 11:**
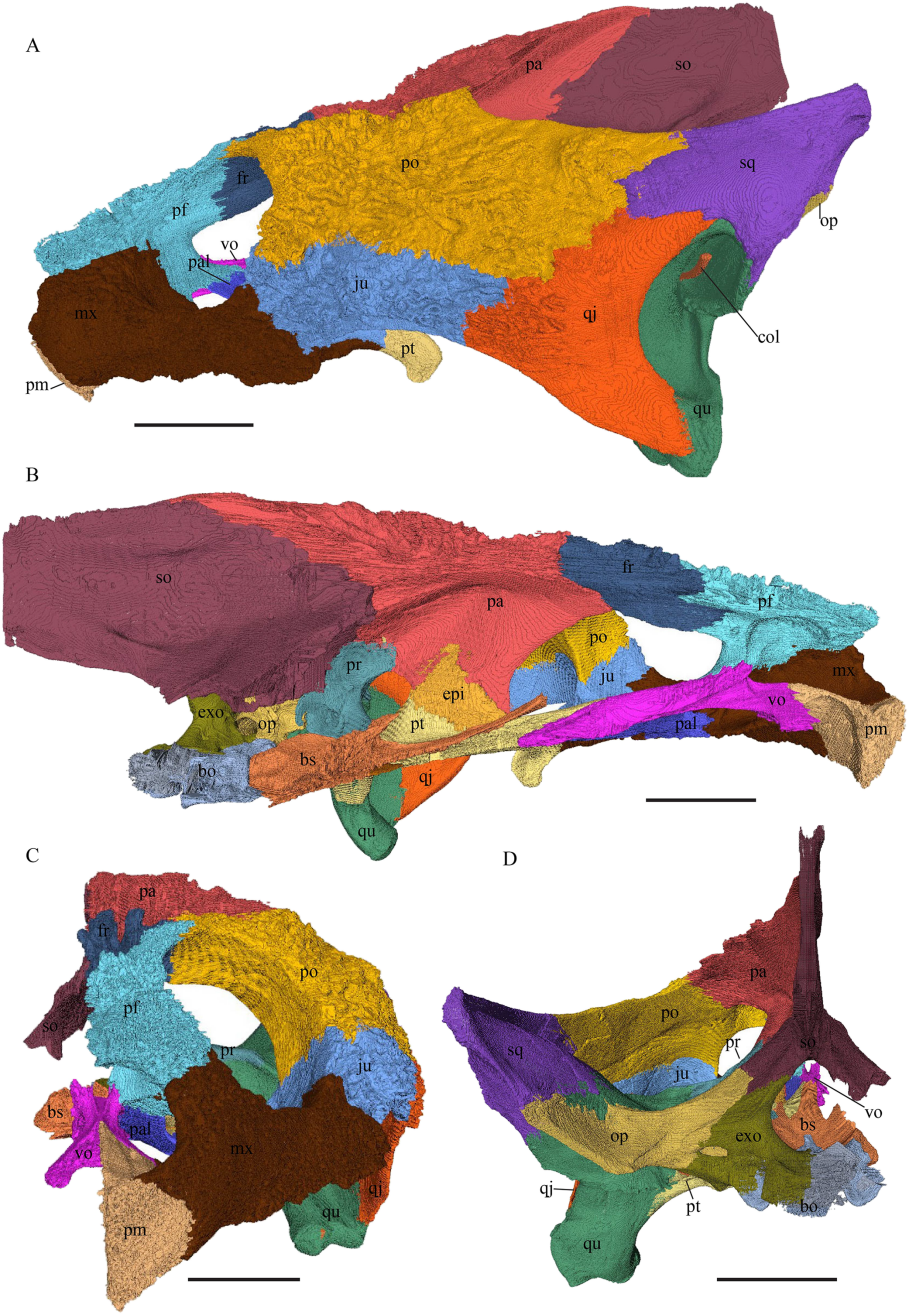
The segmented skull of *Chelydra serpentina*. (A) Lateral; (B) medial; (C) anterior and (D) posterior views. The bar marks 10 mm.

**Figure 12:**
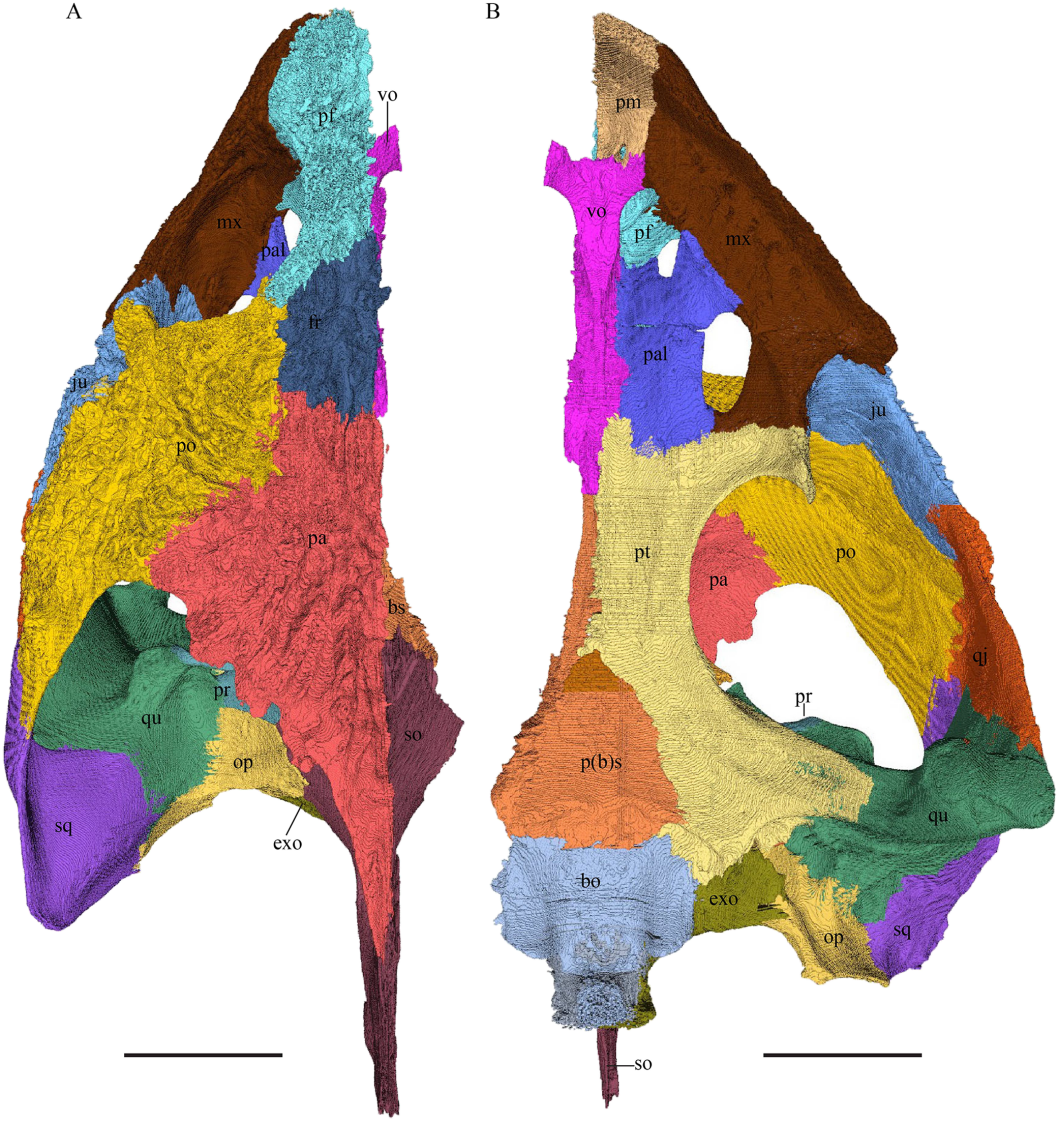
The segmented skull of *Chelydra serpentina*. (A) Dorsal and (B) ventral views. The bar marks 10 mm.

*Chelydra serpentina—*The skull of *C. serpentina* is characterized by a V-shaped outline and strong ornamentation of the skull roof (Figs. 11 and 12). The orbits face laterodorsally and the crista supraoccipitalis is long. This skull is further marked by a deep upper temporal emargination that exposes the prootic and opisthotic in dorsal view. The jaws are narrow and lack lingual ridges.

### Dermal roofing elements (Figs. 13–21)

Nasal (Fig. 13)

**Figure 13:**
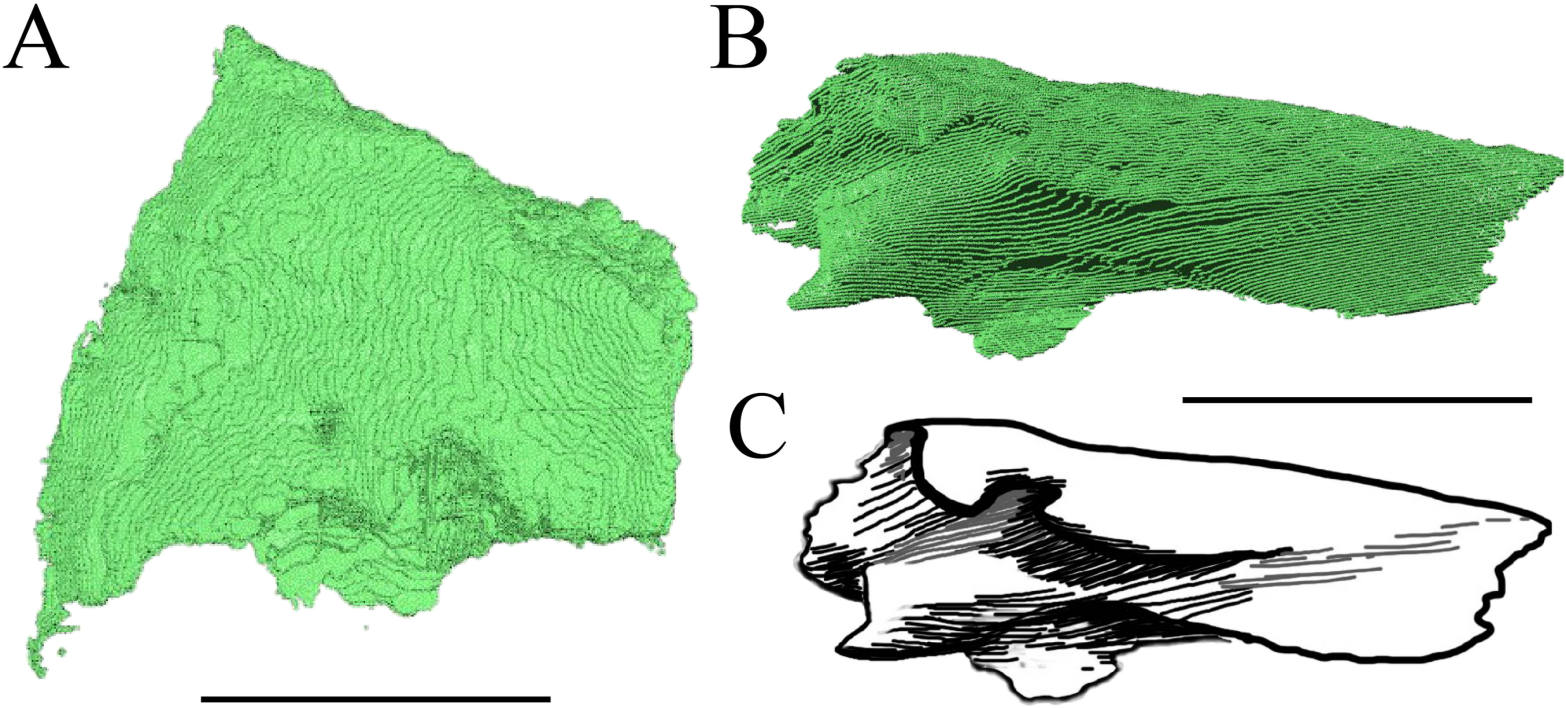
The nasal of *D. lowii*. (A) 3D model in dorsal view; (B) 3D model in anteroventral view; and (C) illustration in anteromedial view, highlighting the parasagittal processes. The scale marks 10 mm.

*Desmatochelys lowii—*In *D. lowii*, the nasal bone is well preserved, except for its anterior margin, which is slightly broken. The nasals contact each other medially in a parallel, slightly interfingering, short suture. The posterior limit of the nasal contacts to two-thirds the frontal and to one-third the prefrontal. The frontal slightly overlaps the nasal dorsally, whereas the nasal overlaps the anterior half of the prefrontal ventrally. The lateral suture of the nasal contacts entirely the maxilla in a slightly interfingering, parallel, posteriorly transverse suture that overlaps the maxilla. The suture between the nasal and maxilla is twice as long as the suture at the midline contact of the nasals. Toward the midline, the nasal bone thins out. An anteromedioventral, hook shaped process and a posteromedioventral, wedge-shaped process form the anteriormost part of the sulcus olfactorius that possibly once supported the septum nasalis (Gaffney, 1979). The nasals participate in the formation of the roof of the fossa nasalis and their anterior edges constitute the upper part of the apertura narium externa.

In *E. imbricata, Dermochelys coriacea*, and *C. serpentina*, the nasal bone is not present.

**Figure 14:**
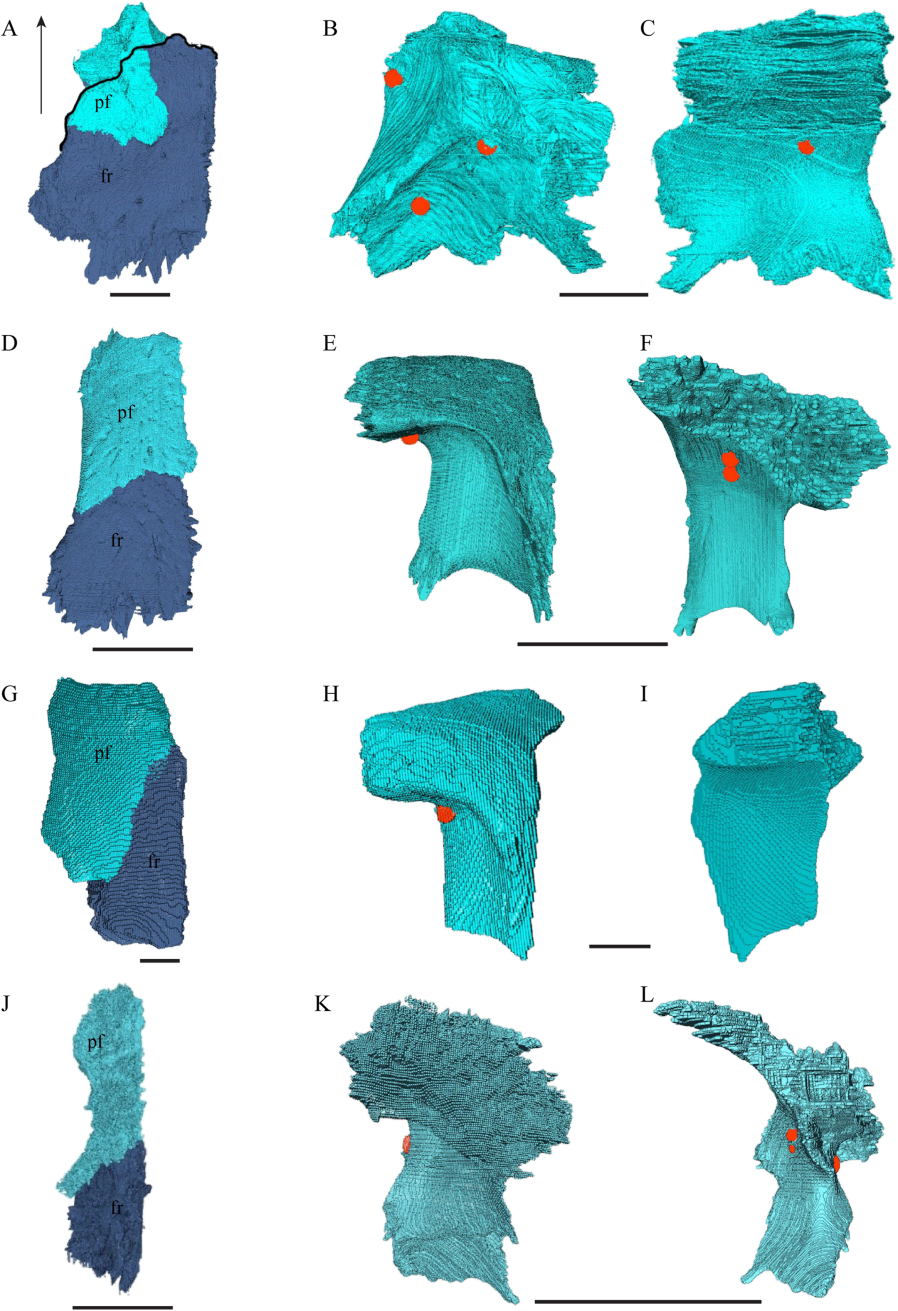
The prefrontal and frontal in dorsal, anterior and posterior views. (A) *Desmatochelys lowii*, dorsal view; (B) *Desmatochelys lowii*, anterior view; (C) *Desmatochelys lowii*, posterior view; (D) *Eretmochelys imbricata*, dorsal view; (E) *Eretmochelys imbricata*, anterior view; (F) *Eretmochelys imbricata*, posterior view; (G) *Dermochelys coriacea*, dorsal view; (H) *Dermochelys coriacea*, anterior view; (I) *Dermochelys coriacea*, posterior view; (J) *Chelydra serpentina*, dorsal view; (K) *Chelydra serpentina*, anterior view; and (L) *Chelydra serpentina*, posterior view. The bar marks 10 mm.

Prefrontal (Figs. 14 and 17)

*Desmatochelys lowii—*The prefrontal of *D. lowii* is heavily sculptured. In anterior view, the prominences and bosses of the bone form a shape that is reminiscent of a lower-case lambda. The posterior, medial, and ventral sides of the prefrontal are dominated by concavities, as the bone participates in the formation of the fossa orbitalis posteriorly, the fossa nasalis medially and ventrally, and the foramen orbito-nasale lateroventrally. The lateral side of the prefrontal is marked by a concavity, of which the vast majority serves as the sutural articulation surface with the maxilla. The posterior process descends toward the palatine and has a tricuspid lower margin. The dorsal exposure of the prefrontal is rather large and appears to be a flat, triangular bone, which is mainly surrounded by the frontal and partly surrounded by the nasal and maxilla. The frontal underlies the prefrontal posteriorly. Medially, the anterior extension of the frontal almost reaches the middle of the height of the prefrontal and overlaps this bone in a rather steep angle. The two prefrontals do not contact each other, as they are separated by the long anterior processes of the frontals in a moderately interfingering suture. The nasal overlaps the anterodorsal half of the prefrontal. Posteroventrally, the prefrontal meets the palatine in a presumably transverse contact at which the palatine seems to overlap the prefrontal. This, however, is not certain as the bones are slightly detached and somewhat displaced along this suture. The anteromedially descending process of the prefrontal contacts the ascending lamellar process of the vomer. The contact changes from anterior to posterior. In the anterior part, the vomerine process overlaps the prefrontal with a relatively smooth contact surface, while along the rest of the contact the two bones are strongly interfingering. However, for most of the anterior half of the contact, the prefrontal overlaps the vomer. In the middle part, the contact between the two bones is parallel, while in the posterior part the vomer once again overlaps the prefrontal. The posterolateral part of the prefrontal participates in the border of the orbit.

*Eretmochelys imbricata—*Contrary to the prefrontals of *D. lowii*, the prefrontals of *E. imbricata* contact each other medially, as the anterior processes of the frontals fully underlie the prefrontals instead of separating them. The contact between the two prefrontals is parallel and moderately interfingering. Each prefrontal participates to a large extent in the margin of the orbit, in the formation of the apertura narium externa, and its rectangular dorsal part is entirely exposed along the skull roof. The prefrontal shows rather simple, angular structures. It does not contact with the palatine and the suture with the maxilla takes up less than a half of the prefrontal’s lateral side. The contact between the prefrontal and the maxilla interfingers strongly and the prefrontal vertically underlies the maxilla’s medial side. The prefrontal meets the vomer in a parallel and strongly interfingering contact. Similar to *D. lowii*, the prefrontal of *E. imbricata* contacts the frontal in a transvers manner posteriorly. Its medially descending process strongly interlocks with the ascending process of the vomer and laterally meets the maxilla. The contact between the prefrontal and the frontal is moderately interfingering and transverse and the frontal underlies the prefrontal. The prefrontal partly forms the fossa nasalis, the fossa orbitalis, and the foramen orbito-nasalis.

*Dermochelys coriacea—*The prefrontals of *Dermochelys coriacea* contact each other anteromedially because the frontals partly separate them. The contact between the two prefrontals is parallel and slightly S-shaped and faintly interfingering. The prefrontal forms the dorsal edge of the apertura narium externa. The subtriangular dorsal surface is fully exposed. The frontal clasps the prefrontal along the anterior half of the contact. The prefrontal does not contact the palatine. The suture with the vomer is extremely short, rather smooth, and vertically transverse, and the prefrontal faintly overlaps the vomer. Only approximately one-third of the prefrontal’s lateral side takes part in the narrow suture with the maxilla, while the rest of it forms the anterodorsal margin of the orbit. Posterolaterally, the prefrontal overlaps the postorbital in a rather strongly interfingering suture. Similar to the prefrontal of *D. lowii*, the prefrontal of *Dermochelys coriacea* connects posteromedially with the frontal by overlapping it. Lateroventrally, the prefrontal meets the maxilla in a faintly interfingering and vertically transverse suture. As in all other here analyzed specimens, the prefrontal takes part in the formation of the fossa nasalis, the fossa orbitalis, and the foramen orbito-nasalis.

*Chelydra serpentina—*As in the other modern species herein examined, the prefrontals of *C. serpentina* contact each other medially in a parallel, moderately interfingering suture. The prefrontal forms the dorsal edge of the apertura narium externa, which is different from the condition in *D. lowii*. The dorsal part is entirely exposed and elongate. Posterolaterally, the prefrontal overlaps the postorbital in a short, faintly interfingering suture. The ascending process of the vomer is clasped by the descending process of the prefrontal in a rather strongly interfingering suture. Laterally, the prefrontal meets the maxilla in a broad suture. In the anterior part of the latter suture, the two bones meet in a strongly interfingering, vertically transverse suture, by which the prefrontal overlaps the maxilla’s medial side. In the posterior part of this suture, the bones meet in a parallel, moderately interfingering suture. Near the midline, the prefrontal clasps the frontal posteriorly in a moderately interfingering suture, while further to the outside, the two bones meet each other in a parallel, moderately interfingering suture. Similar to *D. lowii*, the prefrontal contacts the palatine. The contact between these two bones is transverse and the prefrontal overlaps the palatine in a moderately interfingering suture. The prefrontal is rather strongly sculptured and the ventral concavity is remarkably pronounced.

**Figure 15:**
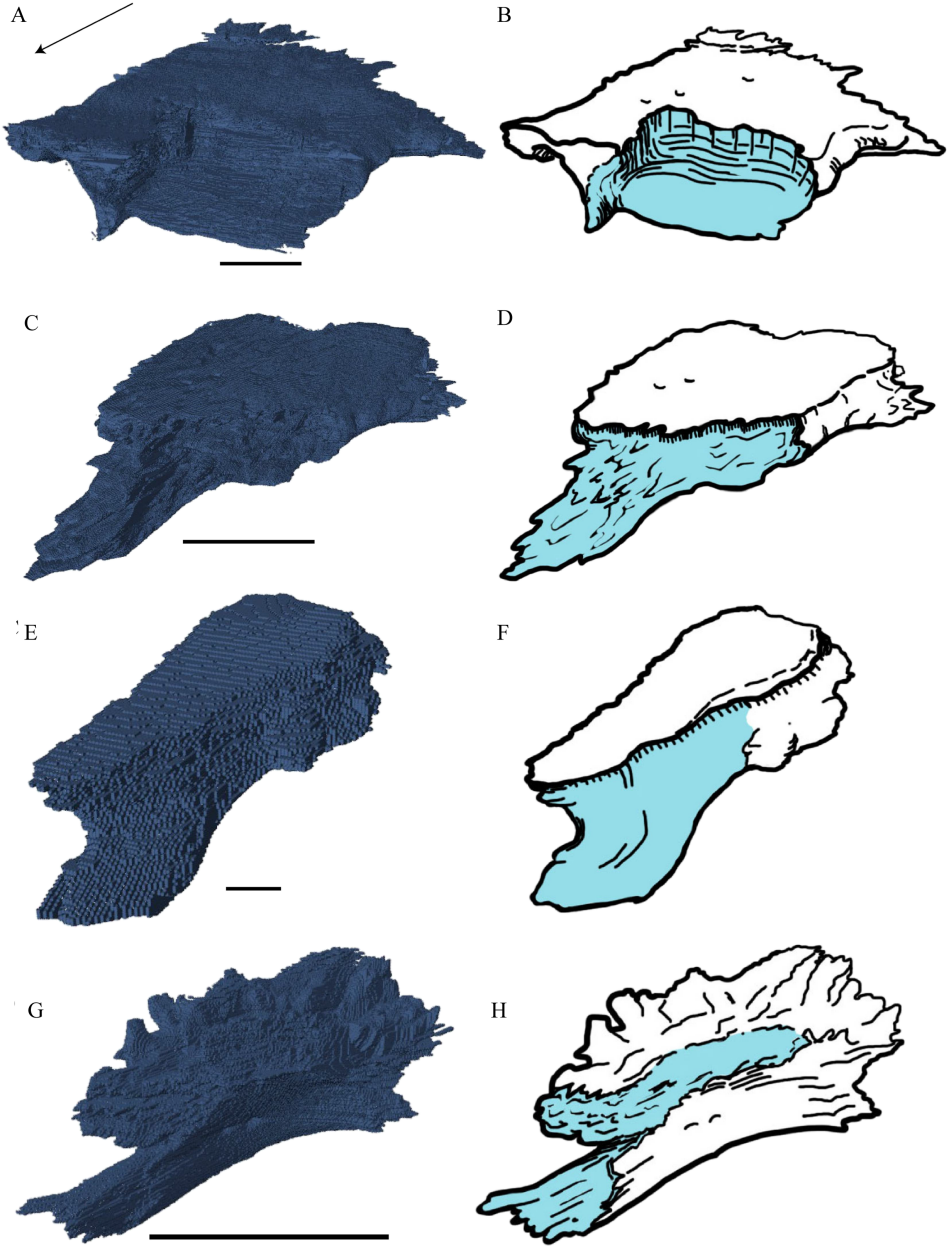
The frontal in anterolateral view of the 3D model and illustration. (A) *Desmatochelys lowii*, 3D model; (B) *Desmatochelys lowii*, illustration; (C) *Eretmochelys imbricata*, 3D model; (D) *Eretmochelys imbricata*, illustration; (E) *Dermochelys coriacea*, 3D model; (F) *Dermochelys coriacea*, illustration; (G) *Chelydra serpentina*, 3D model; and (H) *Chelydra serpentina*, illustration. Light blue surface indicates the suture area with the prefrontal. The bar marks 10 mm.

Frontal (Figs. 14, 15 and 17)

*Desmatochelys lowii—*In *D. lowii*, the frontal is well preserved, except for a small posterodorsal part that appears taphonomically deformed. The dorsally exposed part of the bone, the dorsal plate, is flat and its geometry resembles the letter “P”. The dorsal plate of the frontal is approximately four times larger than the dorsal plate of the prefrontal. The lateral extension of the posterior half of the dorsal plate is approximately three times wider than the dorsal part of the anterior process. The ventral part of the anterior process is as long as the anteriormost edge of the dorsal plate. A particularly notable structure is the suture of the frontal with the prefrontal which forms a shallow, bay-like depression in the anterolateral part of the frontal. Ventromedially, a parasagittal ridge is visible. Along the transverse plane, this ridge forms a step between the roof of the sulcus olfactorius and the lower situated bottom of the suture with the prefrontal. The frontal bones contact each other medially in a parallel, moderately interfingering suture. Posteromedially, the frontal overlaps the parietal in a moderately interfingering suture. Posterolaterally, the frontal underlies the postorbital in a moderately interfingering suture. Anterolaterally, the frontals underliy the prefrontals and separate them from one another with the anterior process. Anteriorly, the frontal meets the nasal in a smooth, parallel suture, and further laterally in a transverse suture, along which the frontal overlaps the nasal. The frontal contributes to the dorsal margin of the orbit, the sulcus olfactorius, the fossa orbitalis, and the fossa nasalis.

*Eretmochelys imbricata—*The dorsal plate of the frontal of *E. imbricata* shows a very rounded, subtriangular shape, which is different from *D. lowii*. The dorsal plate of the frontal is approximately the same size as the dorsal plate of the prefrontal. The posterior half of the dorsal plate of the frontal is slightly broader than the anterior half. The anterior process of the frontal is not exposed on the skull roof, shows a tongue-like shape, and does not separate the prefrontals. The ventral part of the anterior process is much longer than the anteriormost edge of the dorsal plate. Further, the medial flank of the process is steeper than the lateral its lateral flank, but unlike *D. lowii* does not form a step on the ventral surface of the frontal. Similar to the frontal in *D. lowii*, the frontals contact each other medially in a parallel, moderately interfingering suture. The frontal contacts the parietal medially in a transverse, moderately interfingering suture, while in the more lateral part of the contact, the frontal is clasped by the parietal, while the suture is strongly interfingering. Posterolaterally, the frontal is overlapped by the postorbital in a moderately interfingering suture. However, the sutures between these three bones are somewhat steeper than in *D. lowii*. Anteriorly, the frontal underlies the prefrontal in a moderately interfingering suture. The frontal participates in the orbit, the sulcus olfactorius, the fossa orbitalis, and the fossa nasalis.

*Dermochelys coriacea—*The dorsal plate of the frontal of *Dermochelys coriacea* is elongate and subtriangular. The dorsal plate of the frontal is approximately the same size as that of the prefrontal. The posterior half of the dorsal plate of the frontal is slightly broader than the anterior half. Anteriorly, the frontal clasps the prefrontal. Therefore, the frontal separates the prefrontals only partially. The frontal is clasped by the parietal posteriorly and by the postorbital posterolaterally in faintly interfingering sutures. As the prefrontal contacts the postorbital, the frontal is excluded from the margin of the orbit. As in *D. lowii*, the two frontal bones contact each other medially in a parallel and moderately interfingering suture. The ventral part of the anterior process is as long as the anteriormost edge of the dorsal plate.

*Chelydra serpentina—*The frontal bones contact each other medially in a parallel, faintly interfingering suture. The dorsal plate of the frontal resembles the letter “D”. The posterior half of the dorsal plate of the frontal is almost as broad as the anterior half. The dorsal plate of the frontal is half the size of that of the prefrontal. The frontal clasps the prefrontal in a moderately interfingering suture and they further laterally meet in a parallel, moderately interfingering suture. The ventral part of the anterior process is much longer than the anteriormost edge of the dorsal plate. The tips of the anterior processes of the frontals are strongly curved and together form a tunnel through which the olfactory (I) nerve passes. Posteriorly, the frontal meets the parietal. Near the midline, the frontal overlaps the parietal in a strongly interfingering suture, while in the further lateral part, the two bones meet in a parallel, moderately interfingering suture. Laterally, the frontal meets the postorbital in a parallel, moderately interfingering suture. As the prefrontal contacts the postorbital, the frontal is excluded from the margin of the orbit.

**Figure 16:**
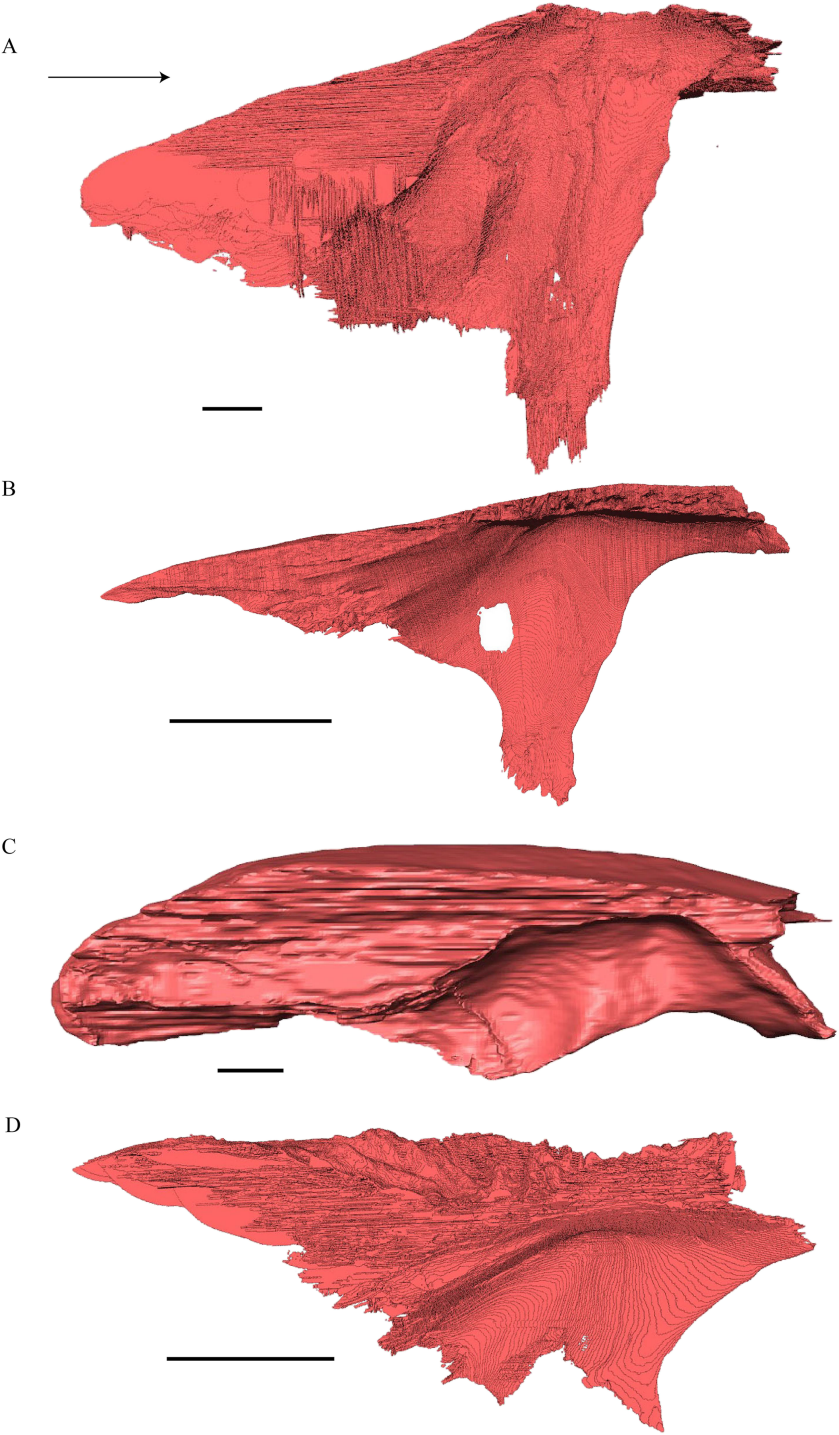
Medial view of the parietal. (A) *Desmatochelys lowii*; (B) *Eretmochelys imbricata*; (C) *Dermochelys coriacea*; and (D) *Chelydra serpentina*. The bar marks 10 mm.

**Figure 17:**
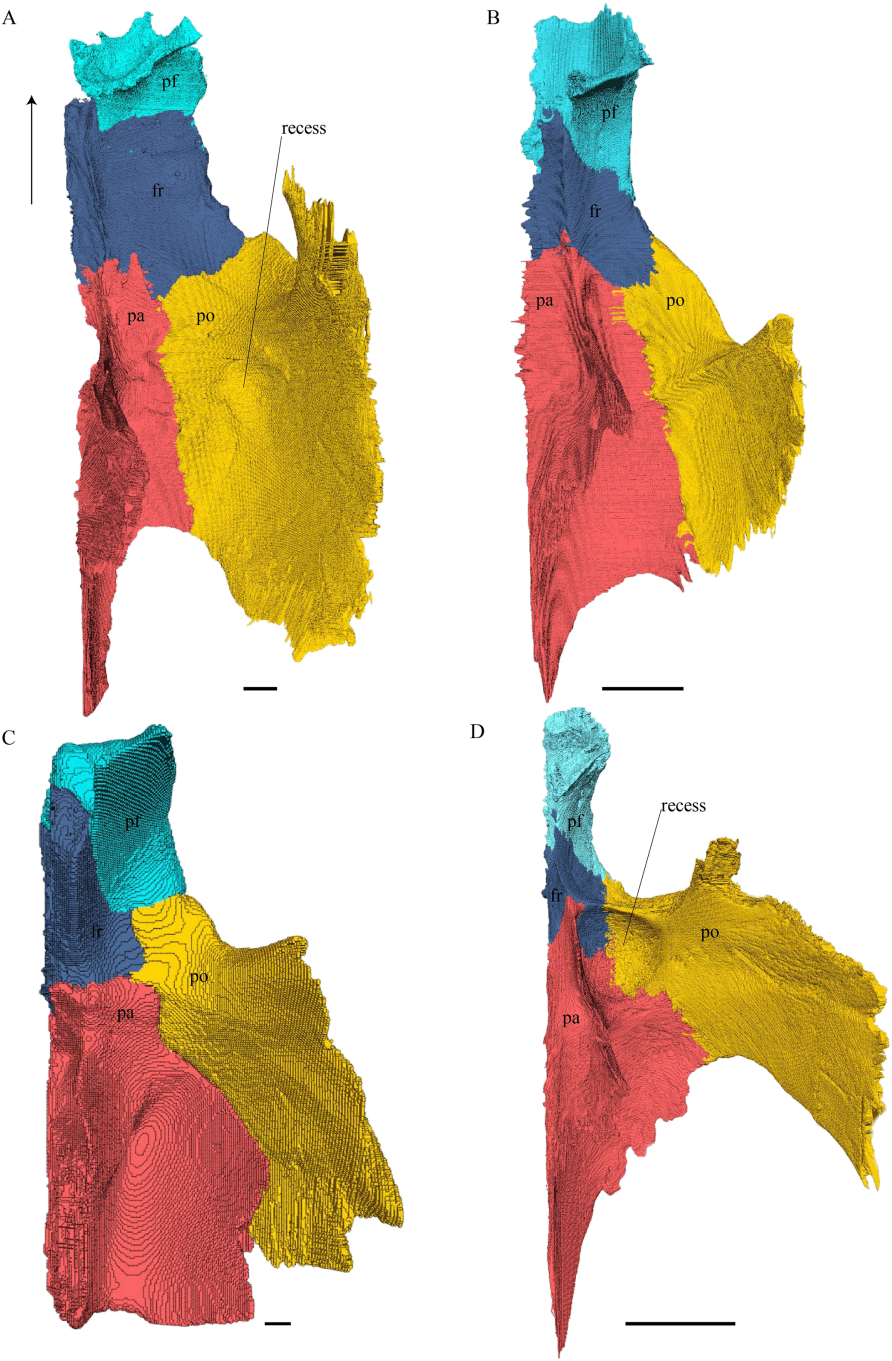
Ventral view of the prefrontal, frontal, parietal, and postorbital. (A) *Desmatochelys lowii*; (B) *Eretmochelys imbricata*; (C) *Dermochelys coriacea*; and (D) *Chelydra serpentina*. The bar marks 10 mm.

Parietal (Figs. 16 and 17)

*Desmatochelys lowii—*The parietal bone of *D. lowii* is slightly damaged. The anterior part of the dorsal plate is considerably domed. The top of this doming has an oval hole, from which a few fractures radiate outward. This hole represents damage on the skull roof and hereby the interpretation of Cadena & Parham (2015) is discarded, who identified this hole as a pineal foramen (see Discussion below). The tip of the posterior process of the parietal seems to be broken, which stands to reason as the posterior part of the crista supraoccipitalis is damaged as well. The posterior suture with the supraoccipital is somewhat indistinct but still vaguely perceptible. The processus inferior parietalis is perforated by some small holes, which appears to be damage to this thinly laminar part of this bone. The two anterior thirds of the dorsal plate of the parietal are as broad as the dorsal exposure of the postorbital. The posterior third of the dorsal plate is much narrower than its anterior part, as it contributes to the formation of the upper temporal emargination. The processus inferior parietalis forms a lateral bulge in the middle and thins out toward its posterior end. On the opposite site of the bulge, on the medial part of the processus inferior parietalis, is a tongue-shaped concavity with a transverse crest that form the lateral wall of the cavum cranii. The dorsal plate of the parietal contacts the other parietal medially in a parallel, moderately interfingering suture. Further, it meets the postorbital laterally in a faintly transverse contact by which, especially in the most anterior and posterior parts of the suture, the postorbital overlaps the parietal in a faintly interfingering suture. Anteriorly, the dorsal plate of the parietal underlies the frontal in a rather flat angle. The extensive anterior part of the processus inferior parietalis meets the epipterygoid at its anteroventral edge in a transverse vertical contact. The rest of the extensive anterior part of the processus inferior parietalis meets the crista pterygoidea of the pterygoid on the lateral part of the margin in a vertically transverse, strongly interfingering suture. The posterior edge of the extensive anterior part of the processus inferior parietalis forms the anterodorsal margin of the foramen nervi trigemini. The lateral bulge of the processus inferior parietalis meets the prootic in a vertically transverse suture at which the parietal slightly underlies the medial part of the prootic in a moderately interfingering suture. In the anterior half of the suture with the supraoccipital, the parietal overlaps the lateral processes of the supraoccipital, while in the posterior half the parietal overlaps the ridge of the crista supraoccipitalis. The parietal contributes to the formation of the cavum cranii medially, the fossa temporalis laterally, the foramen nervi trigemini ventrally, and the foramen interorbitale anteriorly. The parietal is not connected to the squamosal.

*Eretmochelys imbricata—*The parietals of *E. imbricata* both show damage to the processus inferior parietalis where this bone is thinnest. The parietals do not form any hole or pineal foramen at the midline, nor does the dorsal plate form any remarkable elevation. The three anterior quarters of the dorsal plate are broader than the dorsal exposure of the postorbital. The processus inferior parietalis is straight. Laterally, the dorsal plate of the parietal contacts the postorbital in a parallel and moderately interfingering suture. The parietal does not contact the prootic directly. Instead, the two bones are separated from each other by an approximately one mm wide gap. The parietal contacts the squamosal posterolaterally in a very short, parallel, but strongly interfingering suture. The two parietals meet each other medially in a parallel and moderately interfingering suture. Anteriorly, the dorsal plate of the parietal underlies the frontal in a strongly interfingering suture with a rather flat angle. Epipterygoids are not apparent on either side of the skull in the examine specimen, but it is unclear if they never developed or fused with the surrounding bones. The extensive anterior part of the processus inferior parietalis therefore meets the pterygoid in a vertically transverse contact, by which the parietal overlaps the pterygoid’s crista laterally and forms the anterodorsal edge of the foramen nervi trigemini. The posterior half of the parietal contacts the supraoccipital ventrally. Along the anterior part of this suture, the two bones meet each other in a smooth contact, where the parietal overlaps a part of the supraoccipital’s lateral side. In the rest of the suture, the parietal overlaps the supraoccipital, but the two bones meet in a rather strongly interfingering suture.

*Dermochelys coriacea—*The dorsal plate of the parietal is generally twice as broad as the dorsal exposure of the postorbital. The posterior margin of the parietal is not as strongly curved as in the other analyzed taxa, but forms a rather straight border. The processus inferior parietalis is very short and straight. The medial part of the processus inferior parietalis forms a strong concavity in its posterior part and a less pronounced concavity in its anterior part. The processus inferior parietalis only contacts the supraoccipital in a vertically transverse suture and the foramen nervi trigemini is therefore not developed. The postorbital overlaps the parietal in a flat angle along most of their contact, with the exception of the most anterior part of the suture where both bones meet each other in a parallel suture. Anteriorly, the dorsal plate of the parietal underlies the frontal in a steep angle. Posterolaterally, the parietal meets the squamosal. In the anterior part of this suture, the parietal and the squamosal meet each other in a blunt contact, while they both underlie the postorbital. Halfway along the suture, the parietal clasps the squamosal. Toward the posterior end of the suture, the connection between these two bones develops into a transverse contact, along which the squamosal overlaps the parietal. The dorsal plate of the parietal contacts the other parietal medially. The parietal bone contributes medially to the formation of the cavum cranii, laterally the fossa temporalis, and anteriorly the foramen interorbitale. The parietal ventrally overlaps the supraoccipital in a somewhat transverse, smooth to faintly interfingering suture.

*Chelydra serpentina—*The average width of the dorsal plate of the parietal is somewhat broader than the dorsal exposure of the postorbital. The anterior part of the processus inferior parietalis shows a negligible extension. On the opposite side of the lateral bulge, on the medial part of the processus inferior parietalis, is a tongue-shaped concavity forming a part of the cavum cranii, without any crest. Laterally, the parietal overlaps the postorbital in a moderately interfingering suture with a rather steep angle. Anteriorly, the parietal underlies the frontal with a rod-like elongation. The ventral part of the processus inferior parietalis meets the epipterygoid in a vertically transverse, smooth contact, along which the parietal somewhat overlaps the epipterygoid’s medial side. The parietal does not contact the pterygoid. The lateral bulge of the processus inferior parietalis meets the prootic. The parietal bones meet each other medially in a parallel, moderately interfingering suture. The posterior part of the dorsal plate is much narrower, as it contributes to the formation of the upper temporal emargination. The posteroventral edge of the processus inferior parietalis forms the dorsal margin of the foramen nervi trigemini. The processus inferior parietalis forms a lateral bulge in the middle and thins out toward its posterior end. In the anterior part of the suture with the supraoccipital, the parietal partly overlaps the lateral processes of the supraoccipital, while in the posterior half, the parietal shortly overlaps the ridge of the crista supraoccipitalis. In either case, the suture is developed in a moderately interfingering manner. The parietal bone contributes medially to the formation of the cavum cranii, laterally the fossa temporalis, ventrally to the foramen nervi trigemini, and anteriorly the foramen interorbitale. The parietal does not contact the squamosal.

**Figure 18:**
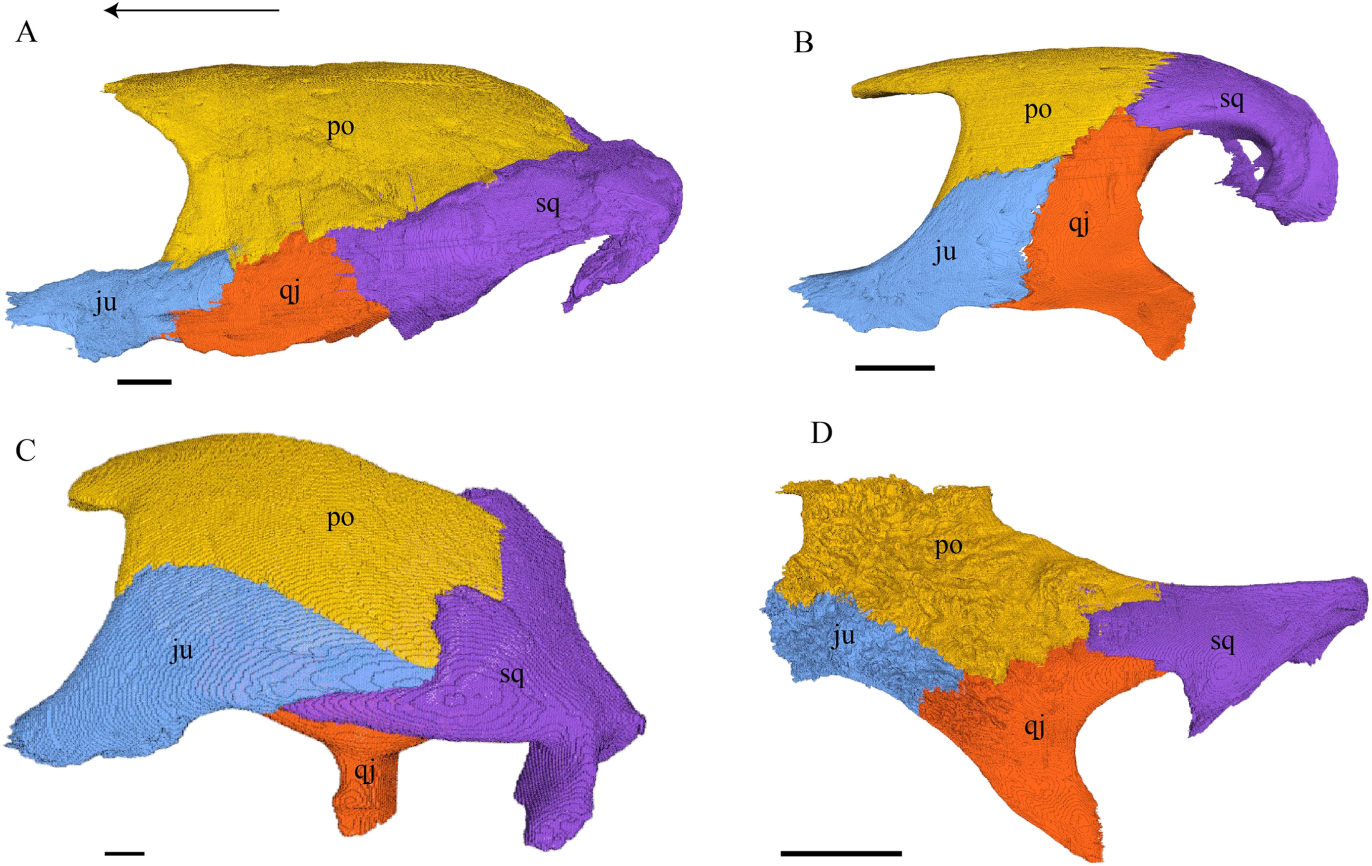
Lateral view of the postorbital, squamosal, quadratojugal, and jugal. (A) *Desmatochelys lowii*; (B) *Eretmochelys imbricata*; (C) *Dermochelys coriacea*; and (D) *Chelydra serpentina*. The bar marks 10 mm.

Postorbital (Figs. 17 and 18)

*Desmatochelys lowii—*The left postorbital of KU VP1200 is damaged, but the right one is mostly intact. The description therefore mostly refers to the right side, although the left is shown in the figures. The postorbital of *D. lowii* is a slightly convex to subangular bone. The posterior part of the bone faintly bends downward and participates in the margin of the upper temporal emargination. An arch-shaped, faintly angular process is developed along the anterior margin of the bone, which forms the posterior border of the orbit. A notch is situated halfway up the orbit. On the medial side of the postorbital, a rounded ridge follows the course of the anterior process. The dorsal part of the postorbital meets the parietal medially, by which especially on the posterior and anterior part of the suture, the postorbital overlaps the parietal in a faintly interfingering suture. Anteriorly, the postorbital overlaps the frontal in a moderately interfingering suture in a flat angle. The bended, posterior part of the dorsal part of the postorbital partly covers the posteromedial ascending part of the squamosal. The suture between the postorbital and the squamosal varies along the sagittal plane. In the anterior part of the suture between those two bones, the postorbital overlaps the squamosal in a rather smooth contact. In the middle of this contact, the postorbital is clasped by the squamosal. Further posteriorly, the postorbital underlies the squamosal in a smooth contact but is clasped by the squamosal again toward the posterior. The anterior quarter of the lateral part overlaps the quadratojugal in a slightly interfingering suture with a rather flat angle. The anterior process of the postorbital clasps the posterior part of the jugal. The postorbital does not contact the palatine nor the prefrontal. The postorbital participates in the formation of the fossa orbitalis and the fossa temporalis.

*Eretmochelys imbricata—*The posterior part of the dorsal part is straight and does not participate in the rim of the temporal roof, as the parietal connects with the squamosal. The anterior margin of the lateral part is angled toward the outside. This protrusion is located at the top level of the orbit. The descending ridge that demarcates the posterior margin of the orbit is more pronounced than *D. lowii*. The suture between the postorbital and the parietal is mostly parallel and interfingering, with parts where the parietal slightly overlaps the postorbital. The posterior part of the postorbital meets the squamosal in a parallel and strongly interfingering suture. The minority of the lateral part contacts the squamosal, while the rest of the lateral part posteriorly meets the quadratojugal in a parallel and strongly interfingering suture. Anteroventrally, the postorbital vertically overlaps the laterodorsal side of the jugal in a moderately interfingering suture. Medially, a ridge follows the course of the anterior process. Anteriorly, the dorsal part of the postorbital overlaps the frontal in moderately interfingering suture with a flat angle. The postorbital does not contact the prefrontal. The postorbital participates in the formation of the fossa orbitalis and the fossa temporalis.

*Dermochelys coriacea—*The postorbital of *Dermochelys coriacea* does not participate in the rim of the upper temporal emargination, as the parietal connects with the squamosal. The notch in the posterior margin of the orbit is somewhat pointy to angled and located at the top level of the orbit. The anteromedial part of the postorbital contacts the frontal in a short and butting suture. Anteriorly, the postorbital underlies the prefrontal in a steep angle. In dorsal view, the suture between the postorbital and the squamosal is zigzagged. The postorbital overlaps the squamosal in a faintly interfingering suture. Medioventrally, the postorbital meets the quadratojugal in an extremely short, vertically transverse contact, along which the postorbital overlaps the quadratojugal’s dorsolateral rim. The jugal bone contacts the entire ventral margin of the postorbital’s lateral part. The postorbital clasps the jugal, except for the middle part of the suture, where the postorbital slightly underlies the jugal. The contact between those two bones is faintly interfingering. The posterior part of the bone (approximately two-thirds) is bent downward. On the medial side of the postorbital, a rounded, weakly noticeable ridge somewhat follows the course of the anterior process. The dorsal part of the postorbital overlaps the parietal especially posteriorly in a flat angle. Posteriorly the postorbital overlaps a considerable part of the posteromedial ascending part of the squamosal. The postorbital participates in the formation of the fossa orbitalis and the fossa temporalis.

*Chelydra serpentina—*The medial ridge of the postorbital of *C. serpentina* is more pronounced than that of *D. lowii*, has a broad base, and follows the course of the orbit. The postorbital meets the parietal medially in a blunt and strongly interfingering suture. Anteriorly, the postorbital underlies the prefrontal. The postorbital meets the squamosal and quadratojugal posteriorly in parallel, moderately interfingering sutures. The anterior part of the postorbital contacts the jugal. In the anterior part of the suture between the postorbital and the jugal, the two bones contact each other in a vertically transverse, faintly interfingering suture, while in the posterior part, the two bones meet each other in a parallel, moderately interfingering suture. The postorbital contributes to the upper temporal emargination. The posterior margin of the orbit is faintly angled slightly above the midpoint of the orbit. The postorbital participates in the formation of the fossa orbitalis and the fossa temporalis.

**Figure 19:**
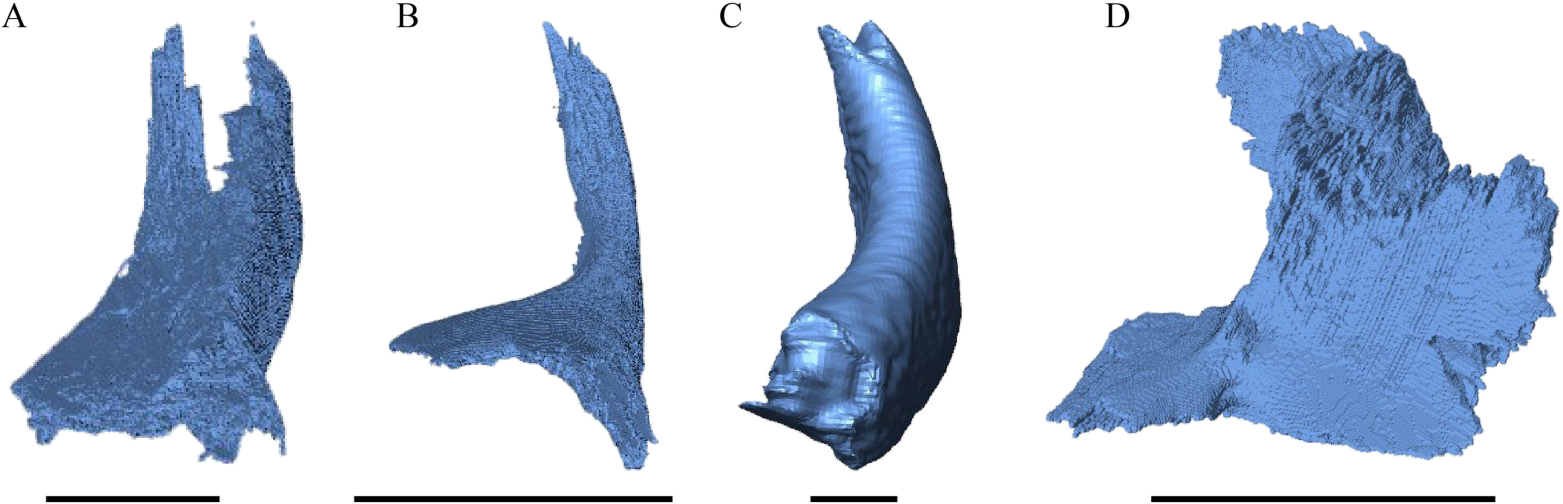
Frontal view of the jugal. (A) *Desmatochelys lowii*; (B) *Eretmochelys imbricata*; (C) *Dermochelys coriacea*; and (D) *Chelydra serpentina*. The bar marks 10 mm.

Jugal (Figs. 18, 19 and 22)

*Desmatochelys lowii—*The posterior limits of the jugal of *D. lowii* are a bit speculative, because of a lack of contrast in the scans. The bone consists of the evenly high, slightly convex lateral wall and the rather short, horizontal medial process. Posteriorly, the jugal probably contacts the quadratojugal in a transverse contact where the jugal overlaps the anterolateral part of the quadratojugal. Posterodorsally, the jugal seems to clasp the lower part of the anterior process of the postorbital. Anteriorly, the jugal overlaps the maxilla in a presumably moderately interfingering suture. The jugal neither contacts the squamosal nor the palatine or pterygoid.

*Eretmochelys imbricata—*The posterior half of the lateral wall of the jugal is strongly elevated and contributes to the margins of the light embayed lower temporal emargination and of the fenestra subtemporalis. Posterodorsally, the lateral wall of the jugal meets the postorbital in a moderately interfingering suture, where the postorbital mostly overlaps the jugal laterally. Posteriorly, the jugal slightly overlaps the anterolateral part of the quadratojugal in a strongly interfingering suture. The medially thinning medial process of the jugal is broad and clasps the palatine. The medial process overlaps the maxilla in a moderately interfingering suture. The jugal neither contacts the squamosal nor the pterygoid.

*Dermochelys coriacea—*The jugal of *Dermochelys coriacea* is rather large and does not possess a medial process. While the upper margin of the lateral wall has a sinusoidal shape, the lower margin is noticeably curved. The jugal laterally covers the anterior half of the quadratojugal’s lateral wall. The suture between the jugal and the postorbital is transverse and overlaps the ventrolateral part of the postorbital. Posteriorly, the jugal overlaps the anterior process of the squamosal. Ventrally, the jugal overlaps the maxilla in a strongly interfingering suture. The jugal neither contacts the palatine nor the pterygoid.

*Chelydra serpentina—*Posteriorly, the jugal meets the quadratojugal in a parallel, faintly interfingering suture. The entire dorsal margin of the jugal contacts the postorbital. The contact is mainly slightly interfingering, except for a short anterior part of the suture where the postorbital somewhat overlaps the medial side of the jugal. The medial process of the jugal overlaps the anterior part of the processus pterygoideus externus. The tip of the medial process of the jugal connects with the palatine in a very short, blunt suture. Posteriorly, the jugal is somewhat clasped by the processus pterygoideus externus in a strongly interfingering suture. The jugal neither contacts the squamosal nor the quadrate. Anteriorly, the jugal overlaps the maxilla in a moderately interfingering suture.

**Figure 20:**
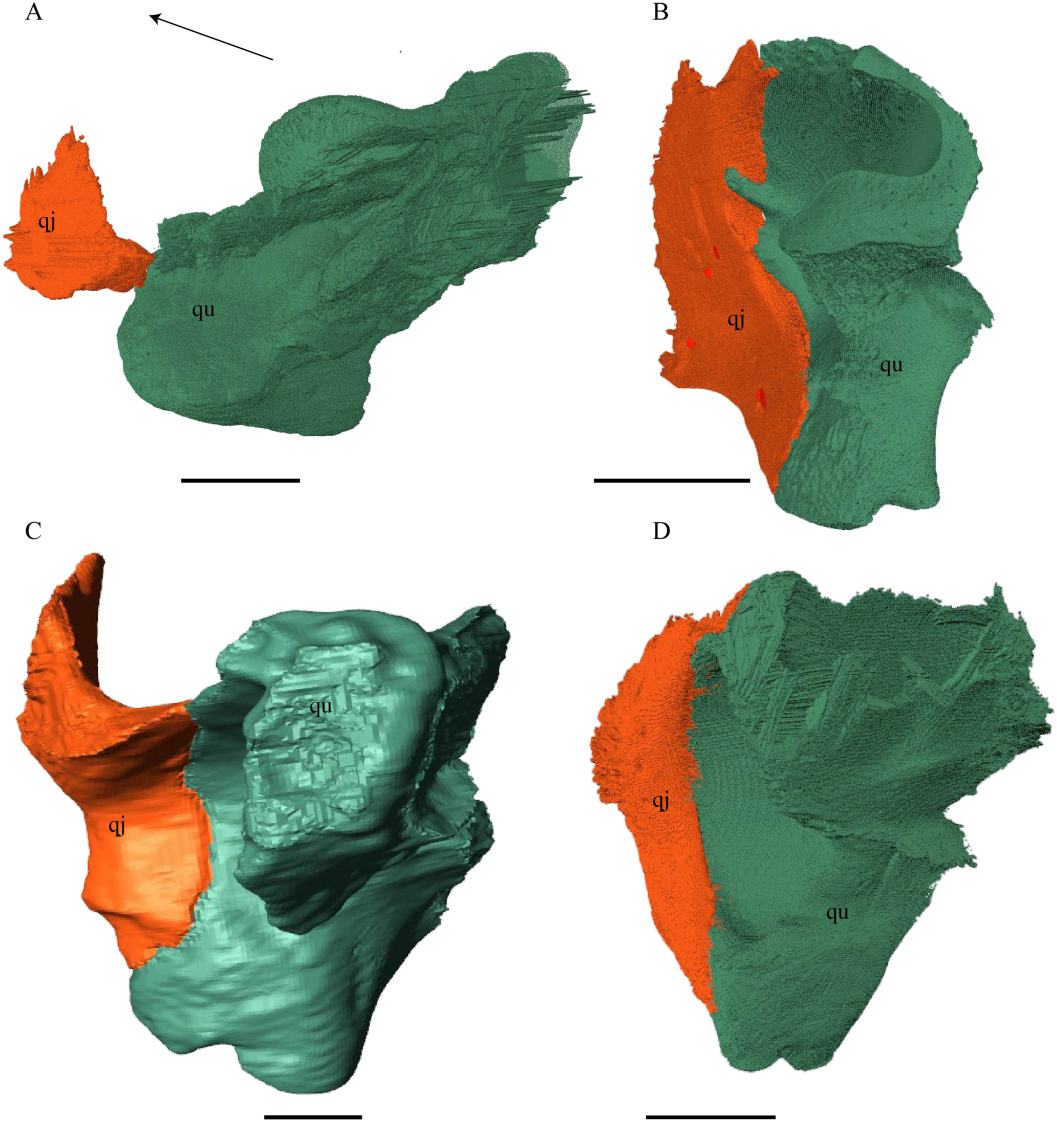
Quadrate and quadratojugal in posterolateral view. (A) *Desmatochelys lowii*; (B) *Eretmochelys imbricata*; (C) *Dermochelys coriacea*; and (D) *Chelydra serpentina*. The bar marks 10 mm.

Quadratojugal (Figs. 18 and 20)

*Desmatochelys lowii—*The contact of the quadratojugal with the jugal is somewhat obscure in the available material. As far as can be discerned, the quadratojugal in this specimen is a parasagittal, flat bone, which is approximately as long as the jugal. The posteroventral margin of the quadratojugal is infolded, but it is unclear if this is natural or caused by damage. The posterior half of the bone is a bit higher than the anterior half, as the dorsal outline of the bone is faintly ascending posteriorly. The lower border of the bone is slightly convex, ventrally. Posteriorly, the quadratojugal covers the anterolateral part of the squamosal and at the very end, the ventral infolding contacts the ventral part of the squamosal. The contact between the quadratojugal and the squamosal is approximately at the same level as the cavum tympani. Dorsally, the quadratojugal meets the postorbital in a transverse, slightly interfingering suture, by which the postorbital overlaps the lateral part of the dorsal border of the quadratojugal bone. Anterolaterally, the quadratojugal is overlapped by the jugal. The quadratojugal forms a part of the lateral wall of the fossa temporalis inferior. Posteroventrally, the quadratojugal meets the quadrate in a short, angulated contact with a smooth suture. The quadratojugal does not participate in the formation of the cavum tympani.

*Eretmochelys imbricata—*The quadratojugal of *E. imbricata* is extended vertically. The bone forms a lateral concavity in its lower third. The concavity somewhat affects the shape of the posterior margin of the bone, which is C–shaped, with a small, tricuspid bump in the middle. On the medial side of the bone, the curvature forms a bulge. The margin of this bulge contacts the anterolateral rim of the quadrate. The two bones meet in a moderately interfingering suture. The posteroventral part of the lateral wall of the quadratojugal forms a flat and rather broad process that descends obliquely. This process covers the anterolateral part of the processus articularis of the quadrate. Along the dorsal margin, the quadratojugal’s lateral side is vertically overlapped by the squamosal in a moderately interfingering suture. The contact between the two latter bones is at the level of the upper margin of the cavum tympani. The lower border of the quadratojugal is strongly curved, as it constitutes the posterior half of the lower temporal emargination. At the ventral part of the anterior border of this bone, there is a pointy process. The upper third of the anterior border of the quadratojugal contacts the postorbital in a parallel and strongly interfingering suture. Two-thirds of the anterior border of the quadratojugal are in a transverse contact with the jugal, by which the jugal overlaps the quadratojugal’s lateral side in a strongly interfingering suture. Noticeable foramina, especially on the lateral but also at the medial side of the bone, indicate canal systems within the quadratojugal. The quadratojugal forms the lateral wall of the fossa temporalis inferior.

*Dermochelys coriacea—*The shape of the quadratojugal of *Dermochelys coriacea* resembles the letter T. It consists of a flat, elongated, lateral wall that thins out dorsally and a posteroventral processus, which is directed ventrally. Medially, the anteroventral part of the quadratojugal overlaps the quadrate in a moderately interfingering suture. The posteriormost part of the quadratojugal is bent inward and connects the quadrate in a parallel, horizontal manner in a faintly interfingering suture. Through this contact, the quadratojugal participate in the formation of the cavum tympani. The lateral wall of the quadratojugal thins out posteriorly and forms a dorsolateral sutural surface. The lower border of the bone is concave to subangular, as it constitutes half of the lower temporal emargination. The posterior half of the lateral wall is clasped by the anterior process of the squamosal in a faintly interfingering suture. The contact between these two bones is above the level of the upper margin of the cavum tympani. The anterior half of the dorsolateral suture surface is covered by the jugal in a faintly interfingering suture, while only the medial edge of the surface connects with the postorbital. The quadratojugal forms the lateral wall of the fossa temporalis inferior.

*Chelydra serpentina—*The quadratojugal of *C. serpentina* consists of a subtriangular lateral. The posterior margin is C-shaped. The ventral border of the quadratojugal forms a minor, sigmoidal curve, as the anterior half of this margin forms the posterior half of the lower temporal emargination. The posterior third of the dorsal margin of the quadratojugal clasps the squamosal in a faintly interfingering suture. The contact between the two latter bones is situated above the cavum tympani. The middle part of the dorsal margin contacts the postorbital in a parallel, moderately interfingering contact. Anterodorsally, the quadratojugal meets the jugal in a faintly interfingering and mostly parallel, slightly transverse contact, by which the jugal partly overlaps the medial side of the dorsal margin of the quadratojugal. The posterior margin of the quadratojugal meets the quadrate along the anterior margin of the cavum tympani. The two bones contact in a moderately interfingering suture. The quadratojugal builds the lateral wall of the fossa temporalis inferior.

**Figure 21:**
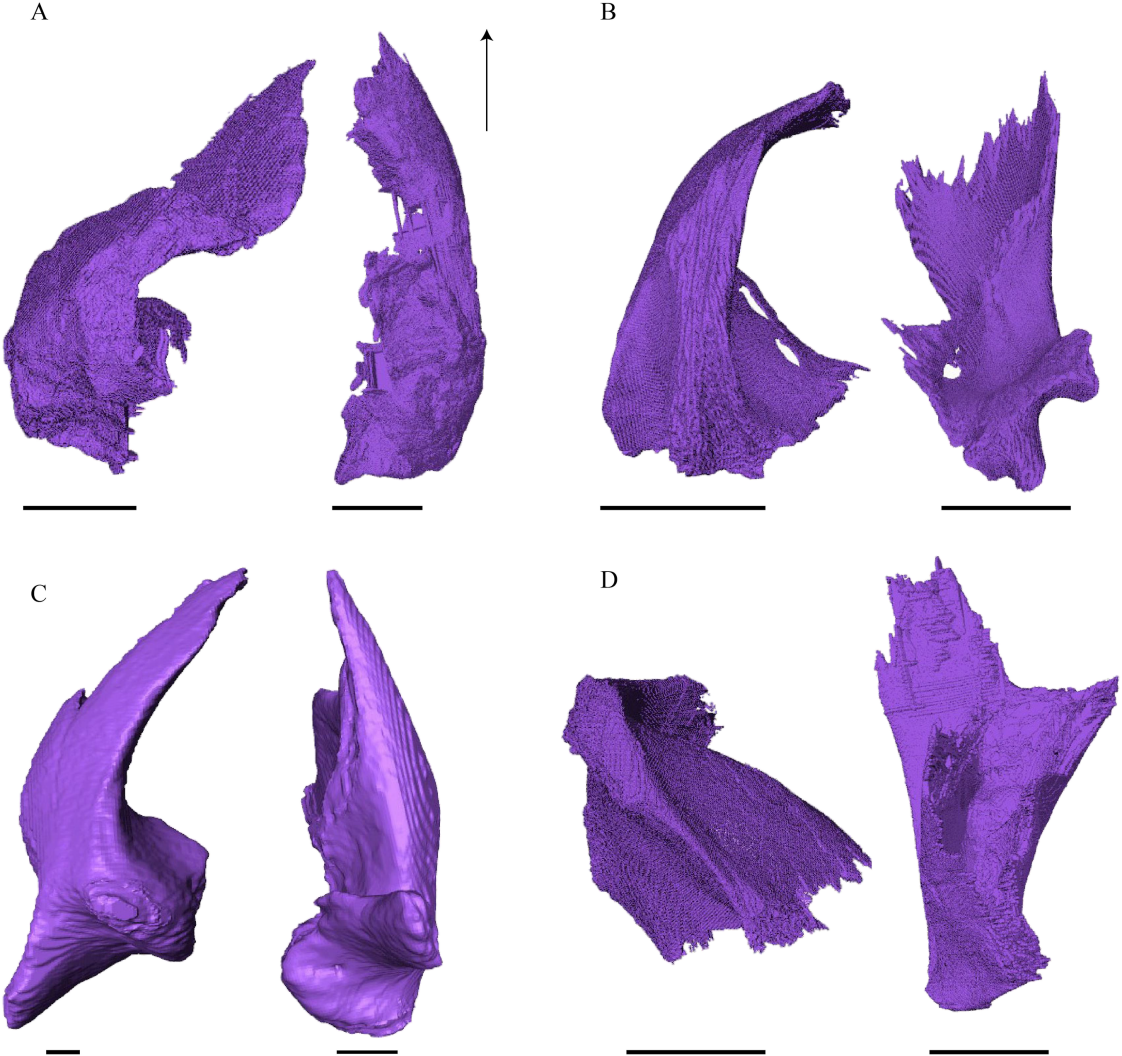
Posterior (left) and ventral (right) view of the squamosal. (A) *Desmatochelys lowii*; (B) *Eretmochelys imbricata*; (C) *Dermochelys coriacea*; and (D) *Chelydra serpentina*. The bar marks 10 mm.

Squamosal (Figs. 18 and 21)

*Desmatochelys lowii—*The squamosal of the available specimen is a bit deformed, especially the posterodorsal part. The course of the sutures between the squamosal and the quadrate and postorbital are somewhat obscure, but nevertheless perceptible. The squamosal consists of two posterior processes and an elongated, convex lateral wall, which is C-shaped in cross section. The squamosal forms an ascending medial process that is covered by the postorbital. The lower process of the squamosal descends anteroventrally and underlies the posterodorsal part of the quadrate in a faintly interfingering suture in an angle of approximately 45°. As a consequence of this contact, the squamosal participates in the formation of the cavum tympani. However, at the very posterior end of the quadrate, the squamosal clasps the quadrate. The entire squamosal is slightly bent anteriorly and posteriorly toward the midline. In addition to the ascending medial process, the squamosal bone meets the postorbital also along the dorsal margin of its lateral wall. This suture varies from anterior to posterior. Anteriorly, the postorbital seems to overlap the squamosal, while in the middle of the suture the squamosal clasps the ventral margin of the postorbital. In the posterior part, the squamosal appears to overlap the postorbital. The lower edge of the posteriormost part of the squamosal contacts the opisthotic in a short and parallel suture. The anteriormost part of the squamosals is laterally covered by the quadratojugal. The squamosal does not contact the parietal.

*Eretmochelys imbricata—*The posteromedial part of the squamosal in the available specimen has two holes caused by damaged to this fragile bone. Differently from the squamosal of *D. lowii*, the squamosal of *E. imbricata* consists of a tilted, laterodorsal plate, whose posterior part is strongly curved downward and bent inward at the same time, which results in a subhemispherical structure. Exteriorly, the squamosal forms two main and one minor parasagittal protuberances with remarkable indentations between them. In medial view, this subhemispherical structure appears as a medial bulge. The ventral margin of the medial bulge for contact with the opisthotic is strongly serrated, but the two bones are slightly detached from each other. It is unclear if this separation is natural or postmortem. Anteriorly, the squamosal meets the postorbital in a strongly interfingering suture. The anterior margin of the medial bulge connects the quadrate in a narrow suture. Posteroventrally, the squamosal is detached from the quadrate by approximately two mm, but both bones show matching surfaces in the area of the corresponding contact. Anteroventrally, the squamosal vertically overlaps the lateral part of the quadratojugal’s dorsal margin. The uppermost, medial part of the squamosal is somewhat elongated and contacts the parietal in a short, parallel, and strongly interfingering suture.

*Dermochelys coriacea—*The squamosal bone of *Dermochelys coriacea* consists of a vertically extended lateral wall and a posteroventral descending process, which is oriented nearly transverse with an outward twist. The anterior half of the lateral wall shows a lowered surface which is the medial suture area for the following bones: the jugal ventrally, the intermediate postorbital, and the parietal dorsally. From medial view, the root of the process forms a medial bulge, which forms an anteriorly thinning, sigmoidal margin along the medial side of the lateral wall. The squamosal contacts the quadrate with the edge of the medial bulge in a rather loose contact. In the anterior part of this contact, the squamosal overlaps the quadrate in a faintly interfingering suture. In the middle part of this suture, the two bones meet in a parallel, moderately interfingering contact. In the posterior part, the quadrate clasps the squamosal. The squamosal in this specimen does not contact the opisthotic. The lowermost part of the anteromedial half of the lateral wall overlaps the quadratojugal. The posteroventrally descending process and the vertical extension of the lateral wall in this specimen are somewhat similar to the two posterior processes of the squamosal of *D. lowii*.

*Chelydra serpentina—*The suture between the squamosal and the quadrate is a bit obscure as the connecting parts of the bones are very thin and tightly sutured. The squamosal is prominently cone-shaped and forms the apex of the antrum postoticum. The cone is directed medioventrally with its apex pointing toward the opposite direction. The interior lateral wall of the cone has a middle crest that reaches the midheight of the chamber at its highest point. Further, the squamosal consists of a subtriangular lateral wall with a flat dorsal margin and a downward-pointing process. The vast majority of the dorsal margin forms the upper temporal emargination. Anterodorsally, the squamosal contacts the postorbital in an approximately parallel and strongly interfingering suture. The cone clasps the upper part of the quadrate in a remarkably tight, faintly interfingering suture. The anteroventral margin of the lateral wall of the squamosal meets the quadratojugal in a predominantly parallel suture, while only in a minor posterior part of the suture, the squamosal laterally overlaps the posterior extension of the quadratojugal. The lower edge of the posteriormost part of the squamosal contacts the opisthotic in a short and parallel, moderately interfingering suture. The squamosal participates in the formation of the antrum postoticum.

**Figure 22:**
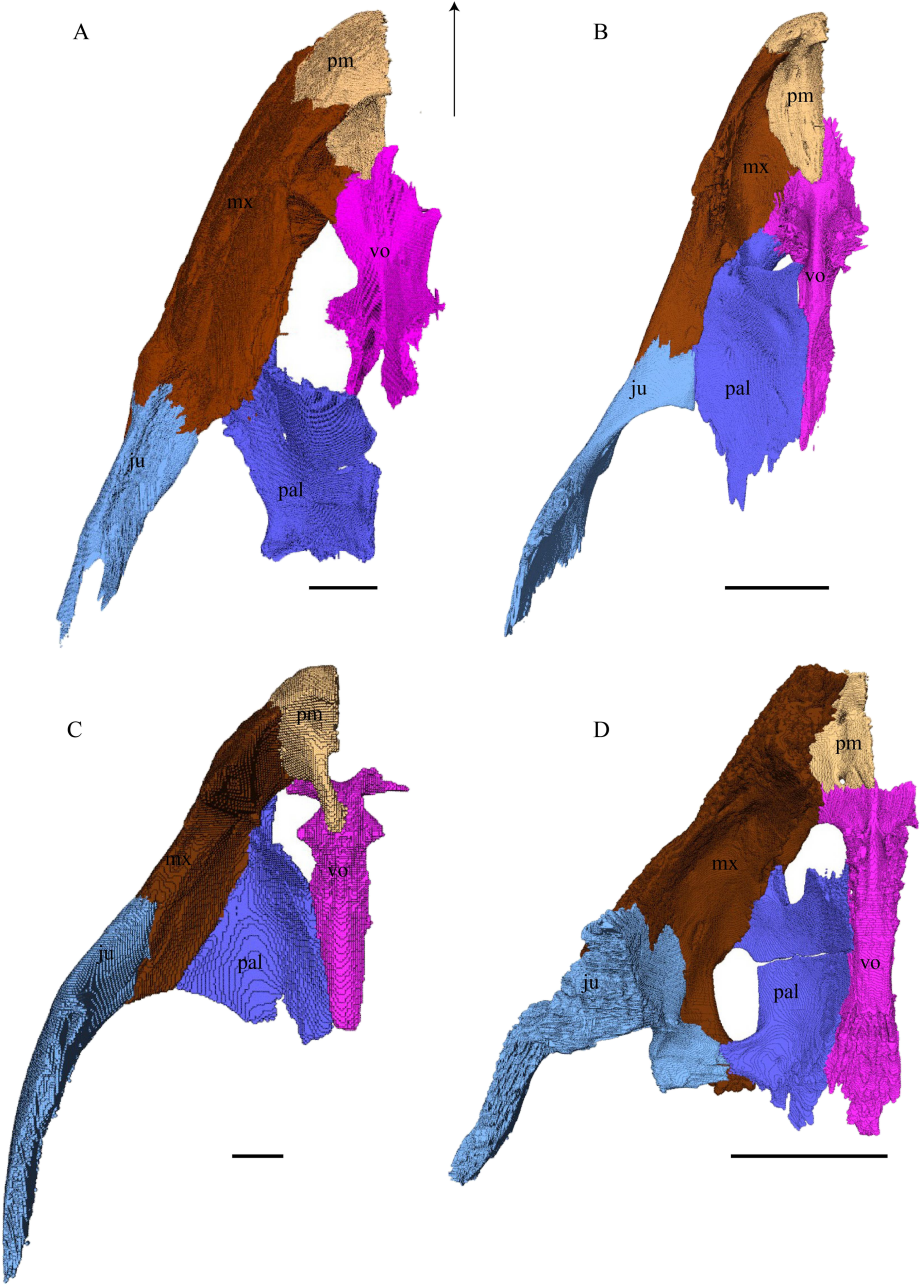
Premaxilla, maxilla, vomer, palatine, and jugal in dorsal view. (A) *Desmatochelys lowii*; (B) *Eretmochelys imbricata*; (C) *Dermochelys coriacea*; and (D) *Chelydra serpentina*. The bar marks 10 mm.

**Figure 23:**
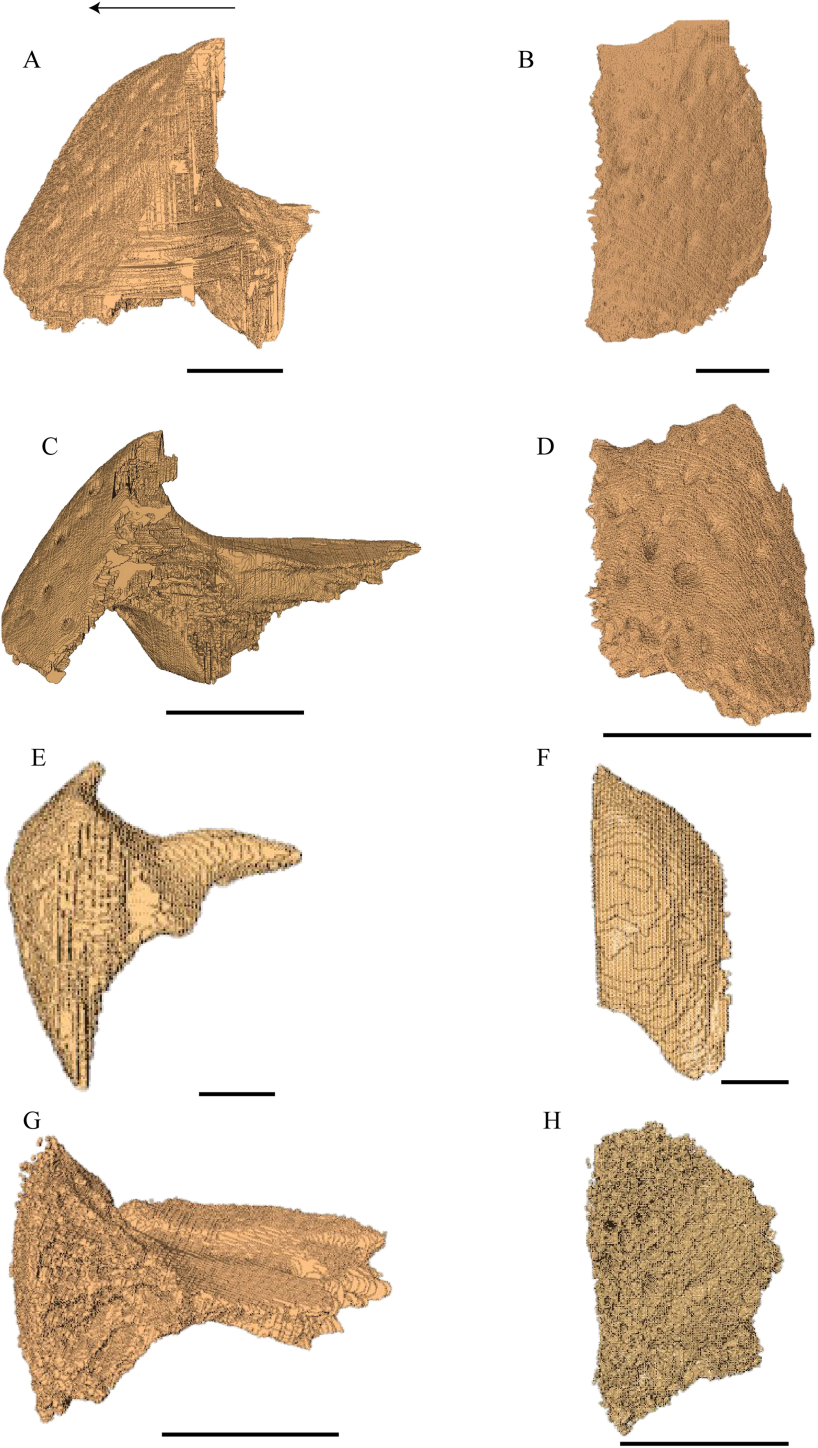
Lateral and anterior view of thepremaxilla. (A) *Desmatochelys lowii*, lateral view; (B) *Desmatochelys lowii*, anterior view; (C) *Eretmochelys imbricata*, lateral view; (D) *Eretmochelys imbricata*, anterior view; (E) *Dermochelys coriacea*, lateral view; (F) *Dermochelys coriacea*, anterior view; (G) *Chelydra serpentina*, lateral view; and (H) *Chelydra serpentina*, anterior view. The bar marks 10 mm.

**Figure 24:**
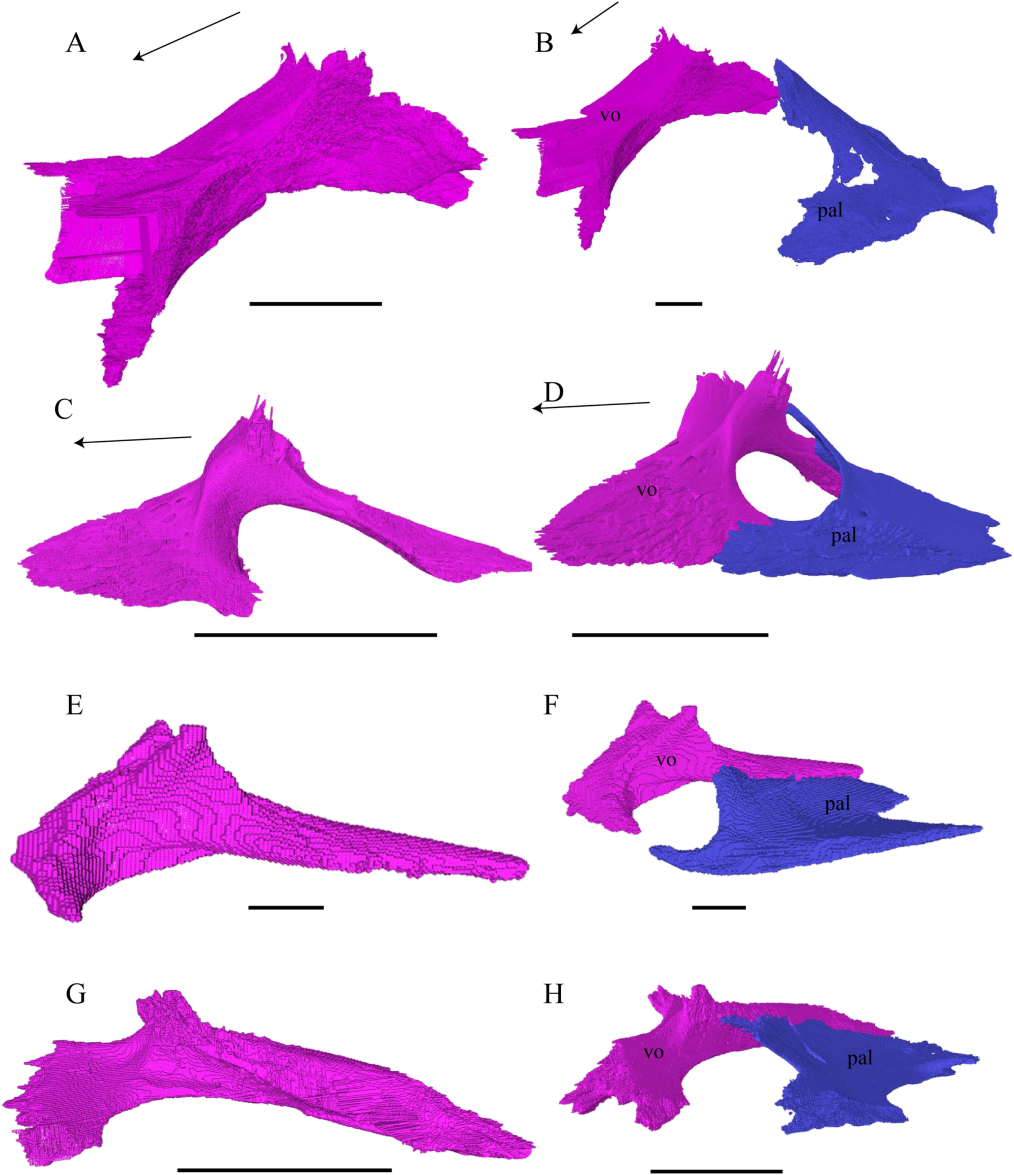
Vomer and vomer with palatine in lateralview. (A) *Desmatochelys lowii*, lateral view of vomer; (B) *Desmatochelys lowii*, anterolateral view of vomer and palatine; (C) *Eretmochelys imbricata*, lateral view of vomer; (D) *Eretmochelys imbricata*, anterolateral view of vomer and palatine; (E) *Dermochelys coriacea*, lateral view of vomer; (F) *Dermochelys coriacea*, anterolateral view of vomer and palatine; (G) *Chelydra serpentina*, lateral view of vomer; and (H) *Chelydra serpentina*, anterolateral view of vomer and palatine. The bar marks 10 mm.

**Figure 25:**
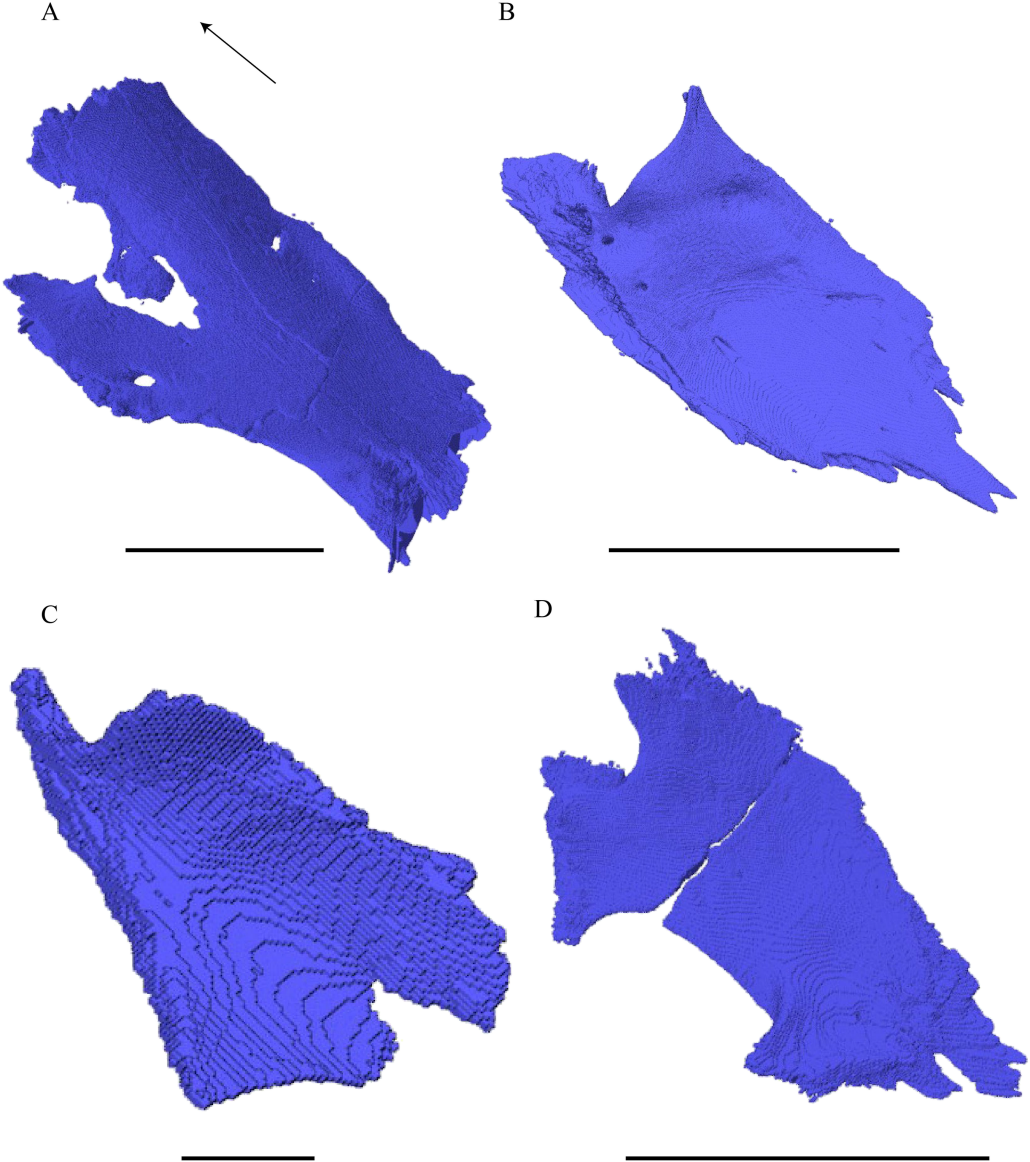
Palatine in dorsolateral view. (A) *Desmatochelys lowii*; (B) *Eretmochelys imbricata*; (C) *Dermochelys coriacea*; and (D) *Chelydra serpentina*. The bar marks 10 mm.

**Palatal Elements (Figs. 22–25)**
Premaxilla (Figs. 22 and 23)

*Desmatochelys lowii—*The premaxilla of the available specimen is relatively well preserved. The anterior lower margin of the bone is lightly convex in lateral view. The suture between the premaxilla and the vomer is a difficult to locate precisely, as the contrast in the scans is low. The same issue applies to the suture between the premaxilla and the maxilla. The premaxilla consists of a slightly backward tilted, frontal plate, which dorsally contributes to the lower edge of the apertura narium externa, and a short, posterior, subhorizontal process, which forms the bottom of the fossa nasalis together with the vomer. In ventral view, the premaxilla forms the anterior margin of the labial ridge, and a small, distinct depression for receipt of the lower jaw. The premaxilla contributes to the anterior margin of the lingual ridge, which is higher than the labial ridge and therefore visible in lateral view. Medially, the two premaxillae contact each other in a parallel, moderately interfingering suture. On the interior part of the frontal plate, there is a foramen, which is connected through a canal to a ventromedial foramen. Laterally, the premaxilla meets the maxilla in a broad, parallel suture. Posteriorly, the premaxilla apparently contacts the vomer in a parallel suture.

*Eretmochelys imbricata—*The premaxilla has a prominent ventral bulge, which is pierced by many canals. The anteroventral curvature of the premaxilla contributes to the labial ridge and the triturating surface. The ventral bulge is at the same level as the labial ridge. The height of the premaxilla measures half of the length of the bone. An unidentified canal exits the premaxilla anteroventrally at the posterior base of the anterior plate of the premaxilla. The upper margin of the anterior plate of the premaxilla contributes to the formation of the apertura narium externa. The lower margin ascends toward the midline, while the upper margin descends. Medially, the premaxillary bones meet each other in a parallel, strongly interfingering suture. Posteroventrally, the premaxilla overlaps the vomer in a strongly interfingering suture. Laterally, the premaxilla overlaps the maxilla in a moderately interfingering, broad suture.

*Dermochelys coriacea—*The premaxilla is as high as its length. The upper margin of the premaxilla contributes to the formation of the apertura narium externa. This margin rises medially and the two premaxillae therefore jointly form a median apex in the middle of the aperture. The ventral margin, which participates in the labial ridge, descends laterally, following the course of the rather deep maxillary part of the labial ridge, where it forms a notch. The posterior process of the premaxilla is rather short, bend upward and slightly lateral, which results in an oval vacuity between the two premaxillary processes. These processes contact each other shortly with their posterior ends, laying on the highest point of the sulcus vomeri. Further, the two premaxillae contact each other medially with their anterior halves, in a parallel, somewhat interfingering suture. The entire lateral surface of the premaxilla connects the maxilla in a parallel, faintly interfingering suture. The premaxilla overlaps the vomer in a rather smooth suture with a low angle.

*Chelydra serpentina—*The height of the premaxilla in this specimen measures half of the length of the bone. The foramen praepalatinum is located in the posterior part of the posterior plate of the premaxilla. At the very front, the lower margins of the premaxillae jointly form a two-cusped median hook. The premaxilla of *C. serpentina* shows a flat, plate-like posterior process. This posterior plate is slightly oblique, tilted lateroventrally, and thins out medially, while its posteriormost part is rather thick and pierced by the vertical foramen praepalatinum. The two premaxillae contact each other in a parallel, mostly moderately interfingering suture. Laterally, the premaxilla meets the maxilla in a moderately interfingering suture. Posteriorly, the vomer slightly clasps the premaxilla in a moderately interfingering suture.

Maxilla (Fig. 22)

*Desmatochelys lowii—*The rather large maxilla of the available specimen is crossed by many fractures. The lateral plate and of the labial ridge show signs of damage and subsequent repair. Approximately in the middle of the maxilla’s triturating surface is an artificial hole. The sutures between the maxilla and the premaxilla and between the maxilla and the vomer are somewhat obscured by matrix. The maxilla consists of a lateral wall whose lower part constitutes the processus alveolaris, the upper part the processus praefrontalis, and the medial part the processus palatinus and the lingual ridge. The medial side of the processus praefrontalis is marked by a prominent vacuity. The maxilla has a rounded, strongly pronounced lingual ridge, which protrudes deeper than the labial ridge. The canalis alveolaris superior is dorsally open and appears as a deep groove at the medial base of the processus praefrontalis. The course of this canal is difficult to determine within the bone. The palatine and premaxilla partially participate in the formation of the lingual ridge, but the maxilla forms the majority. The palatine bone clasps the processus palatinus of the maxilla medially, except at the very front of the suture, where the maxilla overlaps the anterior part of the lateral process of the palatine. The maxilla’s posteroventral extension is overlapped by the jugal in a slightly interfingering suture. The contact between the premaxilla and maxilla is parallel. The dorsal edge of the processus praefrontalis is overlapped by the nasal in a slightly interfingering suture. Posterodorsomedially, the maxilla’s processus praefrontalis contacts the prefrontal in a broad suture. In this suture, the maxilla overlaps the prefrontal in a slightly interfingering contact. At the anteromedial part of the labial ridge, the maxilla meets the anterior part of the vomer, in a presumably transverse suture, by which the vomer clasps the maxilla’s anteromedial side. The maxilla is rather high and constitutes the anterior and most of the lower part of the orbit. Further, it participates in the aperturae narium externa and interna and the foramen orbito-nasale.

*Eretmochelys imbricata—*The foramen supramaxillare is situated at the medial side of the bottom of the ascending process. Dorsomedially, the maxilla covers the prefrontal’s anterolateral process in a strongly interfingering suture. The maxilla of *E. imbricata* possess a sharp labial ridge as well as a rather pronounced, crisp lingual ridge. Posteriorly, the maxilla underlies the jugal in a moderately interfingering suture. Anteromedially, the maxilla meets the vomer in a moderately interfingering suture. In the anterior part of this contact, the two bones meet each other in a parallel suture, while in the posterior part the maxilla overlaps the vomer. Posteromedially, the maxilla meets the palatine. In the anterior part of the suture, the maxilla overlaps the palatine in a moderately interfingering suture. In the middle part of this contact, the maxilla is clasped by the palatine in a moderately interfingering suture. Finally, in the posterior part, the two bones meet in a parallel and moderately interfingering suture.

*Dermochelys coriacea—*Anteromedially, the maxilla meets the premaxilla in a parallel, faintly interfingering suture. At the very anterior part of the contact between the premaxilla and the maxilla, the short processus palatinus underlies the premaxilla and nearly contacts the vomer. The maxilla does not possess a lingual ridge, but a remarkably deep processus alveolaris with a notch in the anterior part. Approximately three-third of the medial side of the maxilla contacts the palatine with its short processus palatinus in a mostly parallel, moderately interfingering suture. The prefrontal meets the maxilla at the posteromedial part of the processus praefrontalis in an interfingering and rather narrow suture. Posterodorsally and slightly laterally, the maxilla contacts the jugal in a strongly interfingering suture. At the lateral border of the foramen orbito-nasale the maxilla forms a cone, which points backward and slightly inward.

*Chelydra serpentina—*The maxilla of *C. serpentina* has a labial ridge, but no lingual ridge. The processus alveolaris is rather short and anteriorly bent inward. The processus praefrontalis is remarkably short. The posterior part of the palatine process forms a medially extending hook-shaped process, which contacts the jugal, palatine, and pterygoid. This part of the maxilla is medially clasped by the palatine in a faintly interfingering suture. Dorsally and ventrally this maxillary process is clasped by the pterygoid in a moderately interfingering suture. Posterodorsally, the maxilla’s palatine process underlies the jugal in a moderately interfingering suture. This process constitutes the lateral margin of the foramen palatinum posterius. A little further to the anterior, the maxilla forms together with the palatine and the prefrontal the lateral border of the foramen orbito-nasale. The triturating surface is rather broad. Laterally, the maxilla forms an elevated margin for the orbit. Anteromedially, the maxilla contacts the premaxilla in a moderately interfingering suture. Posterior to this contact, the maxilla overlaps the vomer’s posterolateral process in a faintly interfingering suture. Along the medial edge of the processus praefrontalis, the maxilla meets the prefrontal in a mostly broad and interfingering suture. In the posterior part of the suture, between the latter two bones, the contact thins out.

Vomer (Figs. 22 and 24)

*Desmatochelys lowii—*The unpaired vomer of *D. lowii* consist mainly of two large bent lamellae, which are fused along the midline, and an anterior subhorizontal plate. On the anterior, subhorizontal plane of the vomer, a rugose elongated bulge is situated along the midline. The right lamella is anteriorly pierced by a vertical hole. Ventrally, the vomer forms a crest along the midline along its posterior three quarters, while at the anterior quarter, a triangular structure can be observed. This structure is made of a transverse bulge, in front of which a circular depression is situated. From this triangular structure, two ventral lamellae split off laterally that contact the lingual ridge of the maxilla. The contacts between the vomer and the premaxilla, maxilla, and palatine are difficult to determine in the available specimen. The dorsal edge of each lamella meets the prefrontal in a transverse suture. In the anterior part of this suture, the vomer slightly overlaps the prefrontal in a rather parallel suture, whereas in the middle of the contact, the prefrontal overlaps the vomer in a steep and strongly interfingering suture. Finally, at the posterior part of the suture, the vomer again overlaps the prefrontal in a moderately interfingering suture. At the very posterior, lower end of the lamella, the vomer meets the palatine in a short and interfingering contact. However, this contact is a bit uncertain as the palatine seems to be detached. The vomer meets the maxilla anteriorly and the two bones constitute the anterior rim of the apertura narium interna. It is difficult to determine the type of the contact between these two bones in this specimen, but it seems that the vomer slightly overlaps or even clasps the medial margin of the maxilla’s anterodorsal part of the lingual ridge. Anteriorly, the vomer meets the premaxilla in a presumably parallel suture.

*Eretmochelys imbricata—*The anterior process of the vomer overlaps the palatine in a moderately interfingering suture with a steep angle. In the middle part of the vomer, the palatine shortly connects the vomer posteriorly on the ascending lamella in a smooth suture. Posterolaterally, the palatine overlaps the vomer’s posterior process to an extent that leaves out a dorsally exposed ridge of the vomer and does therefore not contact the other palatine. The posteriormost part of the vomer overlaps the pterygoid in a moderately interfingering suture with a rather flat angle. The anteriorly elongated cone like process of the vomer connects with the premaxilla anterodorsally in a rather flat angle and laterally it contacts the maxilla. The suture between the vomer and the maxilla is transverse whereby in the posterior part of the suture, the maxilla overlaps the vomer in a steep angle, but underlies the medial process of the maxilla anteriorly. The ascending lamella dorsally contacts the prefrontal in a parallel, strongly interfingering suture.

*Dermochelys coriacea—*The posteriormost part of the vomer overlaps the pterygoid, but the two bones only barely touch each other in the available dry specimen. The vomer contacts the palatine only with the lateral edge of its posterior process in a parallel, slightly interfingering suture, except for the posteriormost part of the contact, where the vomer slightly overlaps the palatines in a smooth suture. The premaxilla meets the vomer in a short suture, by which the hook-like posterior process of the premaxilla connects with its tip to the bottom of the sulcus vomeri, situated between the two lamellae. The anterolateral process of the vomer nearly contacts the maxilla. The posterior process of the vomer is not covered by any other bone. The dorsal process of the vomer contacts the prefrontal in a short parallel suture along paired processes. The dorsal parasagittal bulge along the vomer corresponds to the ventral parasagittal groove.

*Chelydra serpentina—*The middle part of the vomer is square-shaped in cross sections. The morphology of the anterior process of the vomer is T-shaped. The two dorsal processes are directed forward. The sulcus vomeri ends anterior to the dorsal processes in a ridge on the anterior process of the vomer. Laterally, the vomer forms a subhorizontal groove, which constitutes the medial wall of the apertura narium interna. Ventrally, the vomer has a crest along its posterior half. The lateral surface of the vomer’s anterior process contacts the maxillary portion of the processus palatinus. The type of the contact of the latter two bones is transverse, by which the maxilla slightly overlaps the vomer in a steep angle. The palatine contacts the vomer laterally along its two posterior thirds in a faintly transverse, almost parallel suture, by which the palatine somewhat overlaps the vomer in a faintly interfingering suture. The frontal surface of the vomer’s anterior process contacts the premaxilla in a strongly interfingering suture. The prefrontal meets the vomer in a strongly interfingering suture on the dorsal processes of the vomer as well as behind the dorsal processes (approximately one-third of the dorsal exposure of the vomer). The posterior process of the vomer is basically clasped by the pterygoids from both sides in a moderately interfingering suture.

Palatine (Figs. 22, 24 and 25)

*Desmatochelys lowii—*The palatine of *D. lowii* is tilted anteriorly by approximately 45° and consists of a main plate and a lateral horizontal plate. The palatine of the studied specimen is thin and therefore shows some erosional damage, including a transverse fracture. It is furthermore somewhat detached from the other palatal bones. The main plate is domed along the parasagittal plane. The lateral plate of the left palatine exhibits a vertical foramen, which is lacking on the right, an asymmetry also observed in *C. serpentina* (see below). It is unclear to me if this is a single foramen palatinum posterius, a foramen palatinum accessorium, or a foramen unrelated to the inframaxillary artery. The lateral plate forms a lateral thickening with a vertical component, which clasps the processus palatinus of the maxilla and constitutes the posterior end of the lingual ridge. Posterolaterally, a thickened part of the palatine participates in the formation of the vertical flange of the processus pterygoideus externus. The two palatine bones meet each other medially in a parallel, rather broad, moderately interfingering suture. Posteriorly, the palatine contacts the pterygoid in an interfingering, mainly transverse suture by which the pterygoid overlaps the palatine. Anteriorly, the palatine meets the vomer in a presumably transverse suture, by which the palatine probably overlaps the vomer. The palatine in this specimen does not contribute to the triturating surface.

*Eretmochelys imbricata—*The posterior margin of the palatine forms a two-pronged elongation in the middle, similar to a fish tail, which results in a strongly serrated suture between the palatine and the pterygoid. The palatine overlaps the pterygoid in a moderately interfingering suture. The palatines are separated from each other by the posterior process of the vomer. Together with the vomer, the palatine forms a sharp ridge anteriorly and laterally to the apertura narium interna. The anteromedial corner of the palatine is somewhat narrowed, elongated, and elevated. Its tip therefore contacts the base of the vomer’s dorsal processes, just underneath the suture of the vomer and the prefrontal. This contact between the palatine’s anteromedial process and the vomer results in a cavity that is less than 0.5 cm large. Ventrally, the lateral process of the palatine forms a prominent lingual ridge. Anterolaterally, the palatine contacts the maxilla in a mostly parallel and moderately interfingering suture, except for the anteriormost part, where the maxilla overlaps the palatine. The posterior quarter of the lateral edge of the palatine is slightly clasped by the jugal in a very narrow suture. The palatine does not contact the prefrontal.

*Dermochelys coriacea—*The palatine of this specimen has an overall subtriangular shape. The anteromedial margin is elevated. There is an indentation in the middle of the posterior part of the bone. This posterior margin interlocks with the pterygoid. Near the midline, the palatine overlaps the pterygoid in a smooth suture, while further to the outside the palatine underlies the pterygoid in a smooth suture. The lateral edge of the palatine contacts the maxilla in a parallel, moderately interfingering suture. The palatine therefore does not contact the jugal. Medially, the two palatine bones are separated from each other by the posterior process of the vomer. The palatine does not contact the prefrontal. The lateral half of the posterior margin of the palatine forms a part of the fossa temporalis inferior’s border.

*Chelydra serpentina—*The palatine in this specimen is broken along a transverse plane. Anteromedially, the dorsal surface of the palatine is overlapped by the prefrontal. Together with the maxilla, the three latter bones form the foramen orbito-nasale and contribute to the apertura narium interna. The middle part of the lateral border participates in the formation of the foramen palatinum posterius. Medially, the two palatine bones are separated from each other by the posterior process of the vomer. The anterolateral and the posterolateral processes of the palatine both contact the maxilla in a transverse suture. The anterolateral process of the palatine is overlapped by the maxilla, while the posterolateral process of the palatine overlaps the maxilla to an extent that the palatine meets the jugal. The palatine and jugal jointly cover the posteromedial process of the maxilla. Posteriorly, the palatine is clasped by the pterygoid in a moderately interfingering suture.

**Braincase Elements (Figs. 26–43)**
Pterygoid (Figs. 26, 27 and 30)

**Figure 26:**
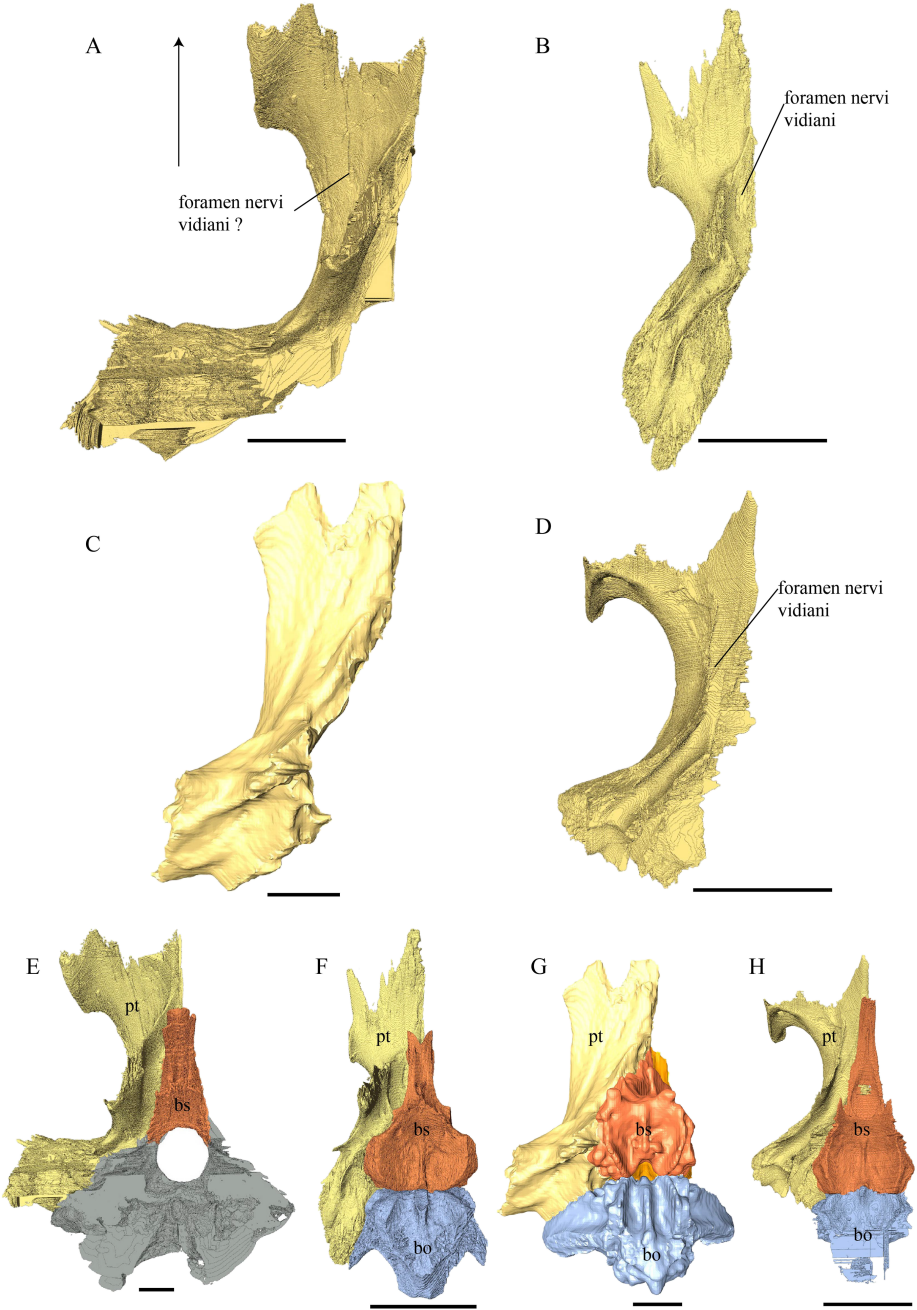
Pterygoid (A–D) and pterygoid, basisphenoid, and basioccipital (E–H) in dorsal view. (A) *Desmatochelys lowii*; (B) *Eretmochelys imbricata*; (C) *Dermochelys coriacea*; (D) *Chelydra serpentina*; (E) *Desmatochelys lowii*; (F) *Eretmochelys imbricata*; (G) *Dermochelys coriacea*; and (H) *Chelydra serpentina*. The bar marks 10 mm.

**Figure 27:**
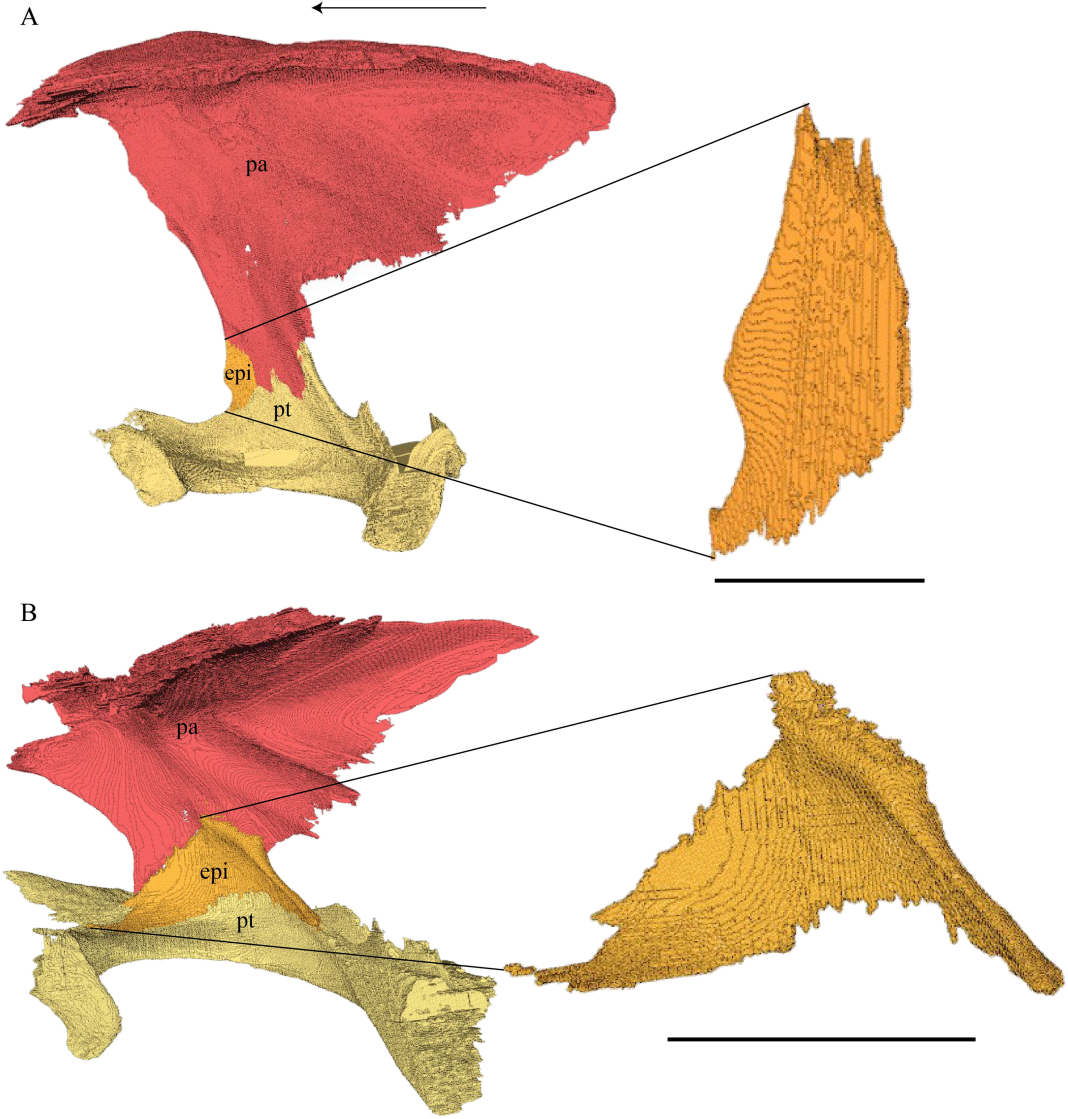
Epipterygoid, parietal, and pterygoid. (A) *Desmatochelys lowii* and (B) *Chelydra serpentina*. The bar marks 10 mm.

**Figure 28:**
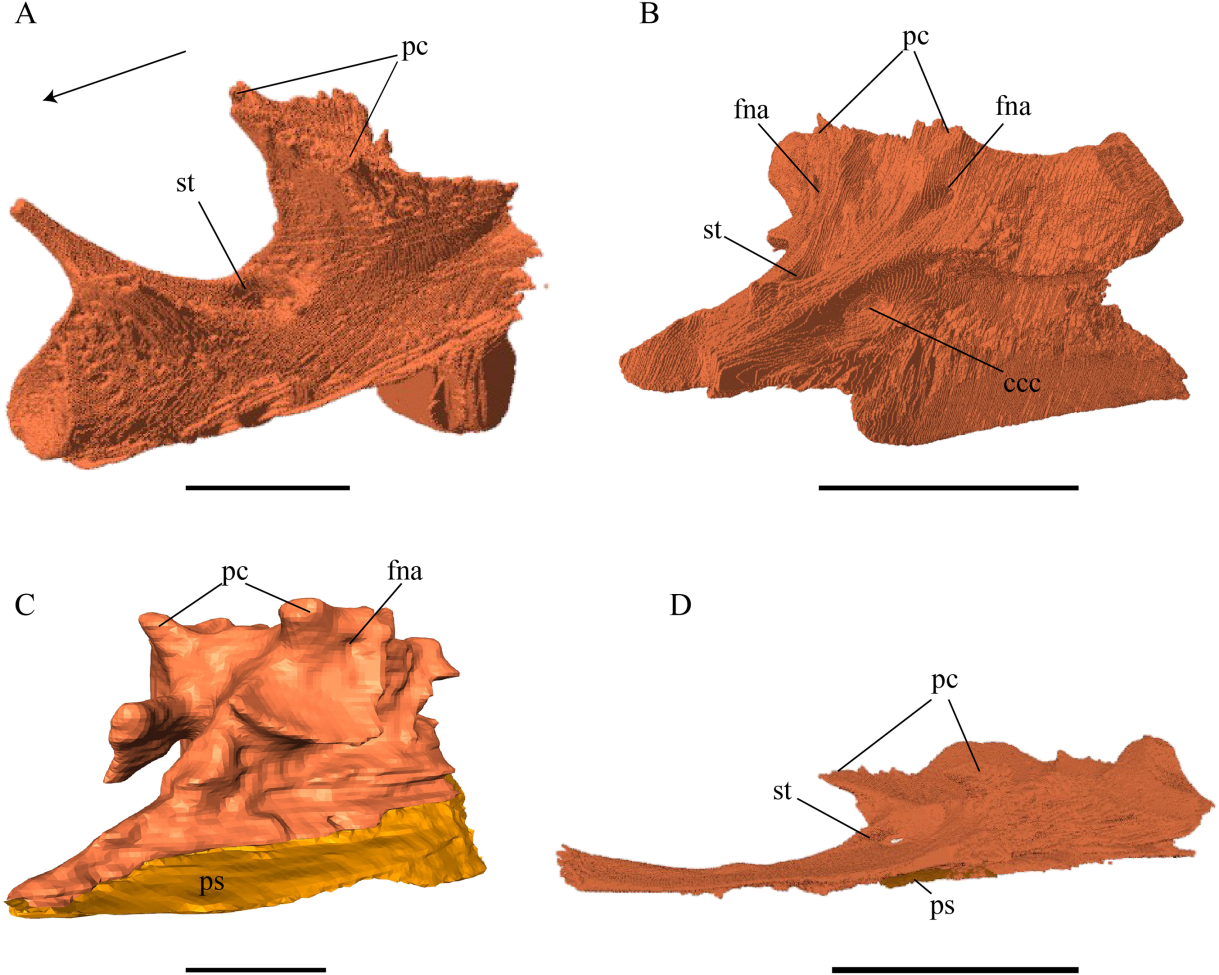
Basisphenoid and parasphenoid in anterolateral view. (A) *Desmatochelys lowii*; (B) *Eretmochelys imbricata*; (C) *Dermochelys coriacea*; and (D) *Chelydra serpentina*. The bar marks 10 mm.

**Figure 29:**
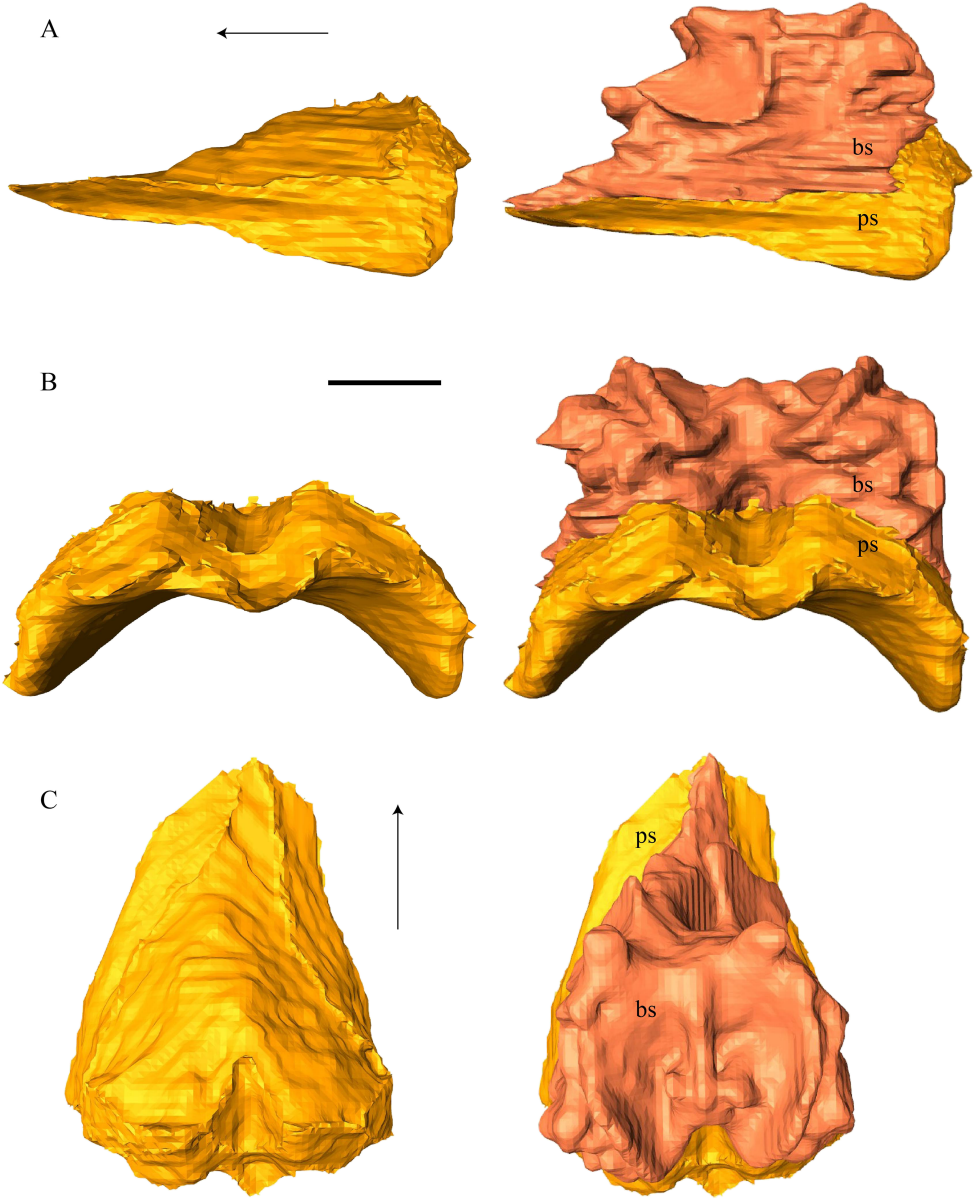
Parasphenoid of *Dermochelys coriacea*. (A) Lateral; (B) posterior; and (C) dorsal view. The bar marks 10 mm.

**Figure 30:**
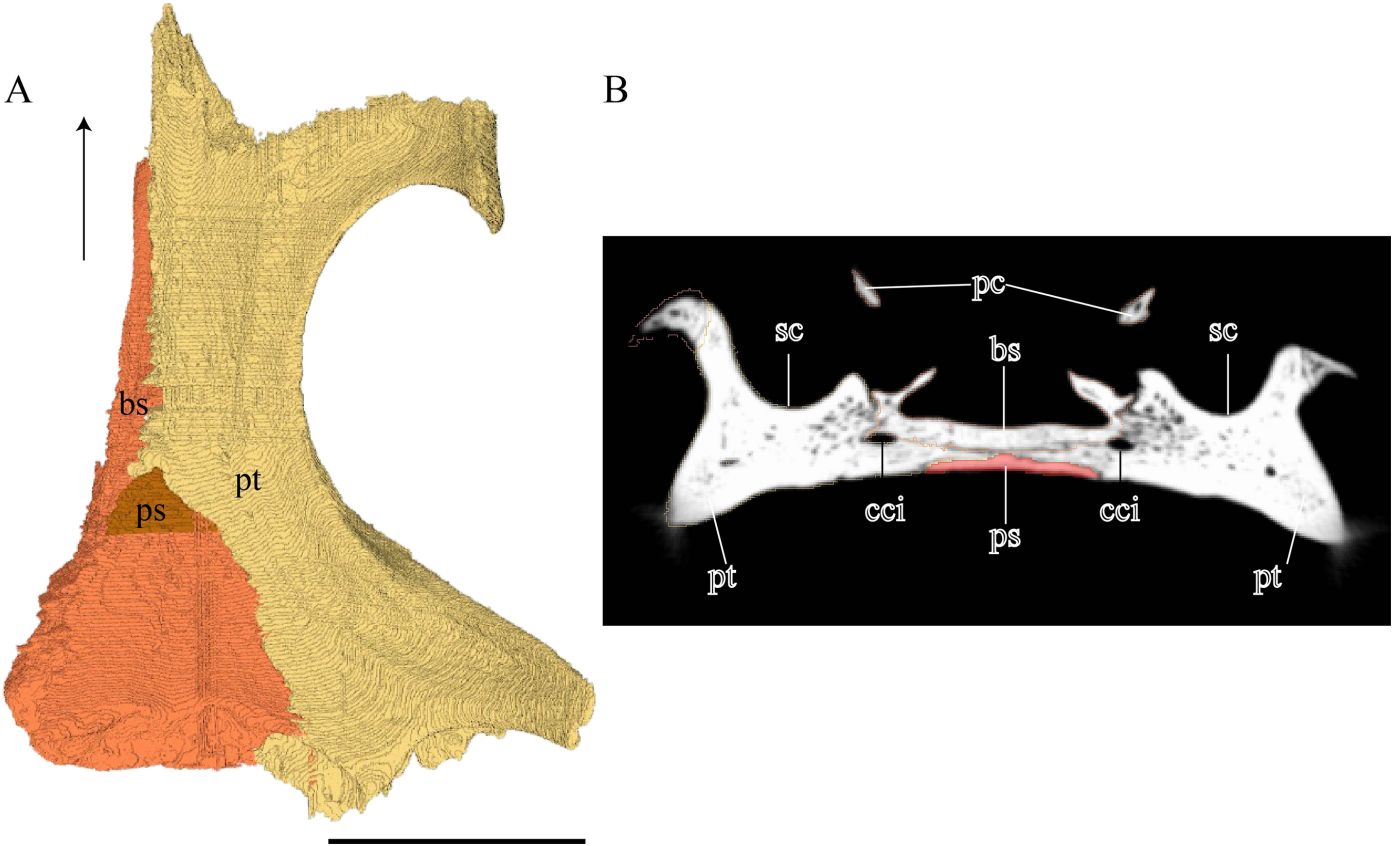
Parasphenoid of *Chelydra serpentine*. (A) Ventral view on the pterygoid, basisphenoid, and parasphenoid and (B) cross section image highlighting the parasphenoid in red. The bar marks 10 mm.

**Figure 31:**
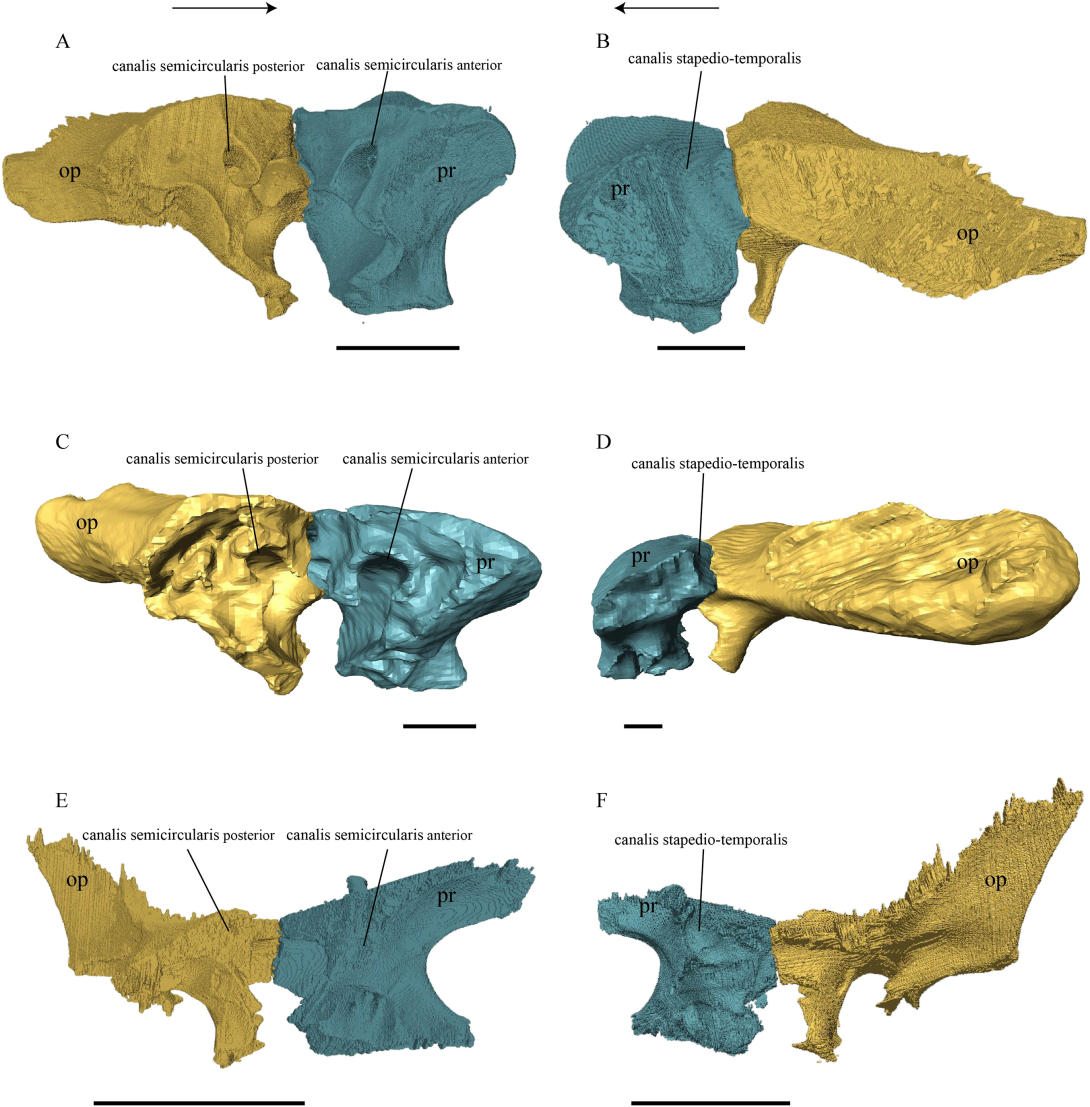
Medial and lateral view of theopisthotic and prootic. (A) *Eretmochelys imbricata*, medial view; (B) *Eretmochelys imbricata*, lateral view; (C) *Dermochelys coriacea*, medial view; (D) *Dermochelys coriacea*, lateral view; (E) *Chelydra serpentina*, medial view; and (F) *Chelydra serpentina*, lateral view. The bar marks 10 mm.

**Figure 32:**
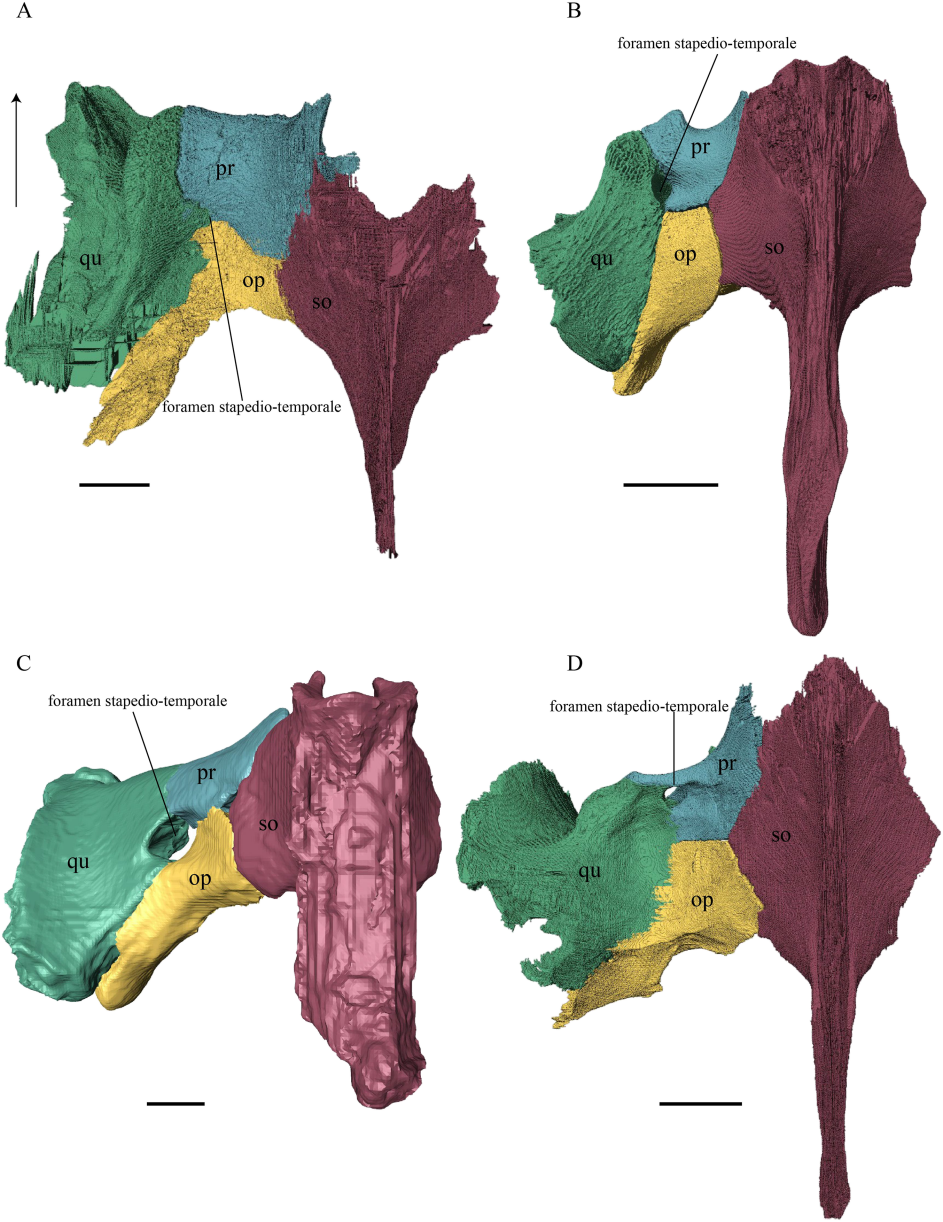
Quadrate, prootic, opisthotic, and supraoccipital in dorsal view. (A) *Desmatochelys lowii*; (B) *Eretmochelys imbricata*; (C) *Dermochelys coriacea*; and (D) *Chelydra serpentina*. The bar marks 10 mm.

**Figure 33:**
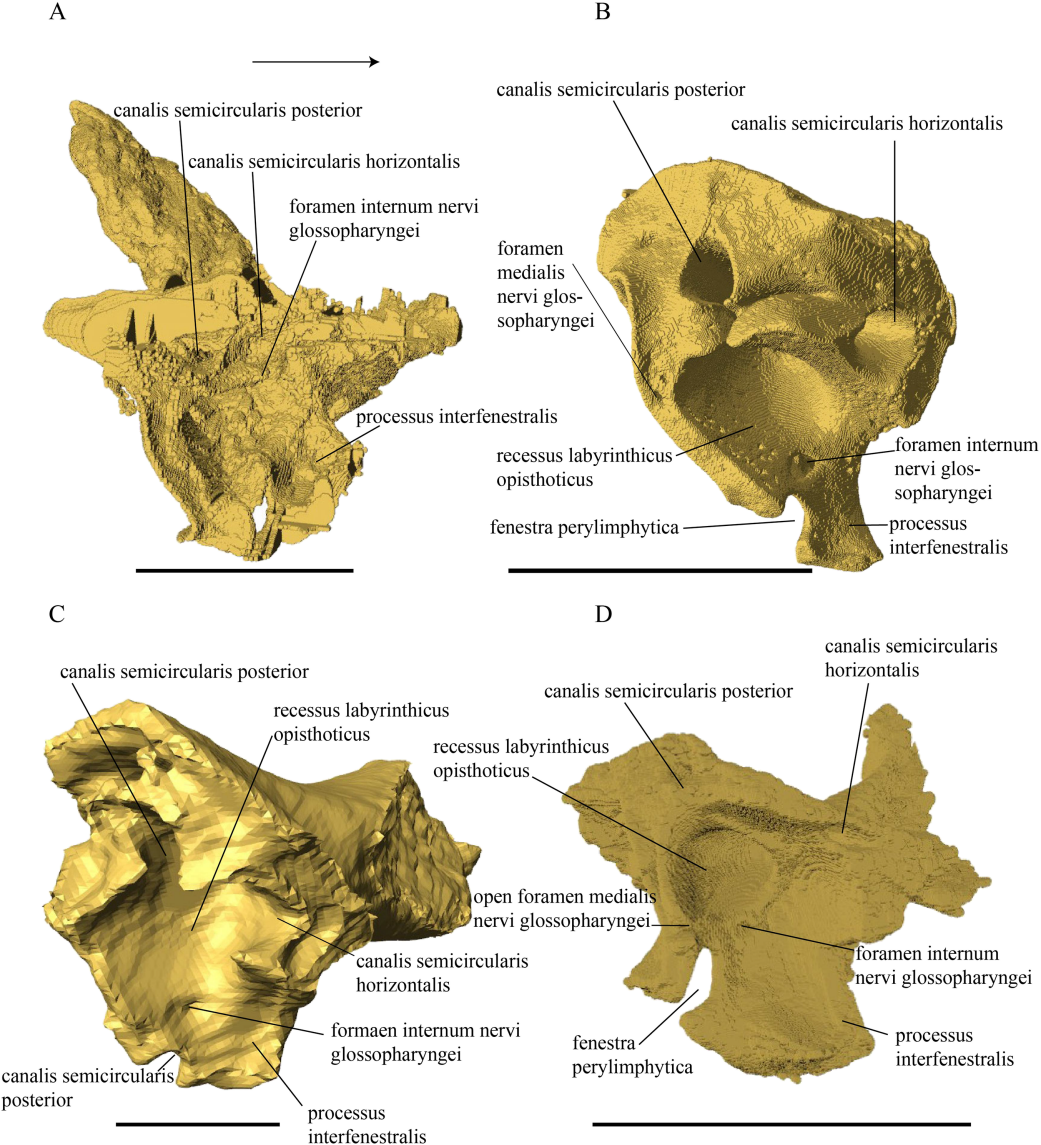
Opisthotic in medial view. (A) *Desmatochelys lowii*; (B) *Eretmochelys imbricata*; (C) *Dermochelys coriacea*; and (D) *Chelydra serpentina*. The bar marks 10 mm.

**Figure 34:**
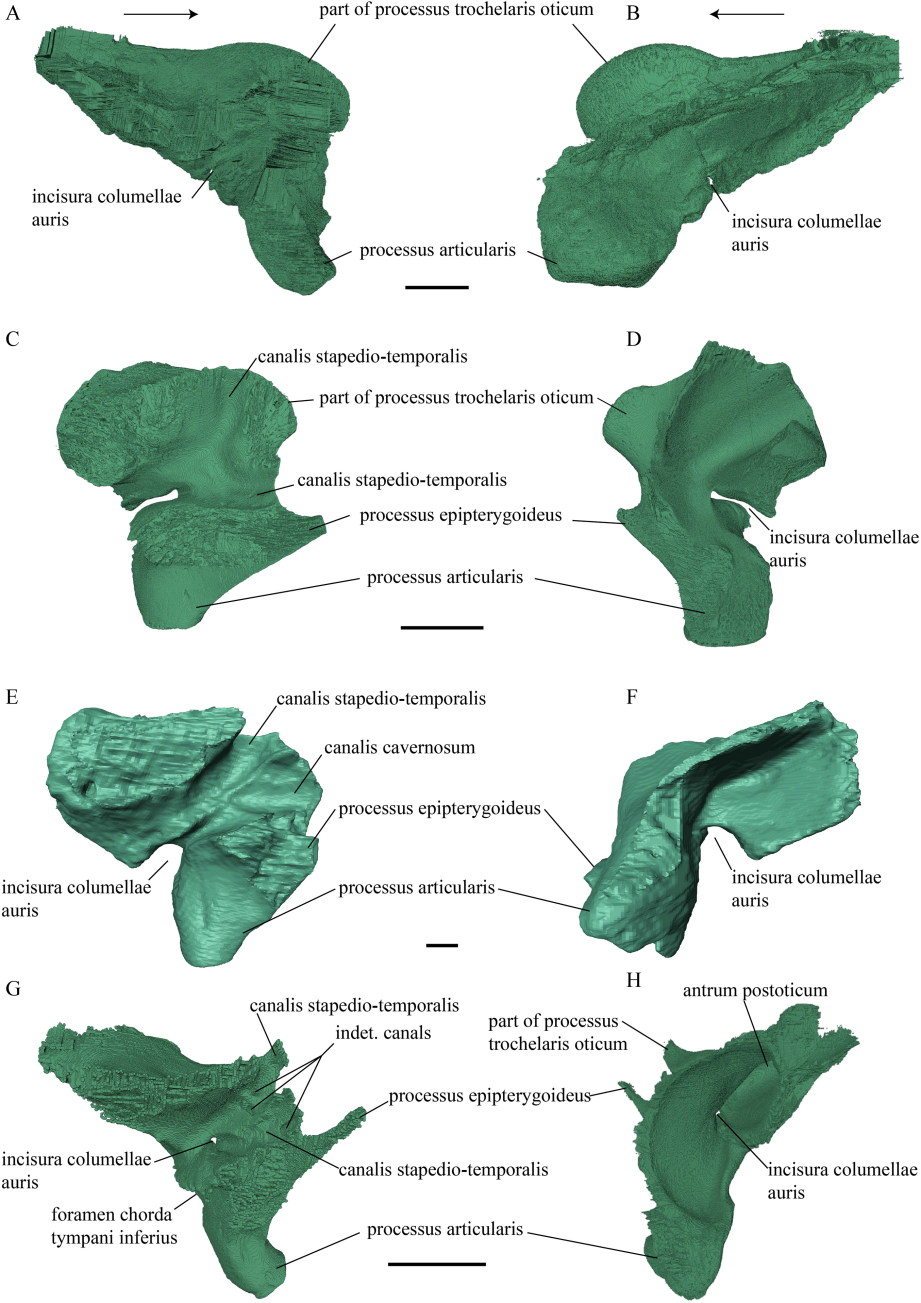
Medial and lateral view of the quadrate. (A) *Desmatochelys lowii*, medial view; (B) *Desmatochelys lowii*, lateral view; (C) *Eretmochelys imbricata*, medial view; (D) *Eretmochelys imbricata*, lateral view; (E) *Dermochelys coriacea*, medial view; (F) *Dermochelys coriacea*, lateral view; (G) *Chelydra serpentina*, medial view; and (H) *Chelydra serpentine*, lateral view. The bar marks 10 mm.

**Figure 35:**
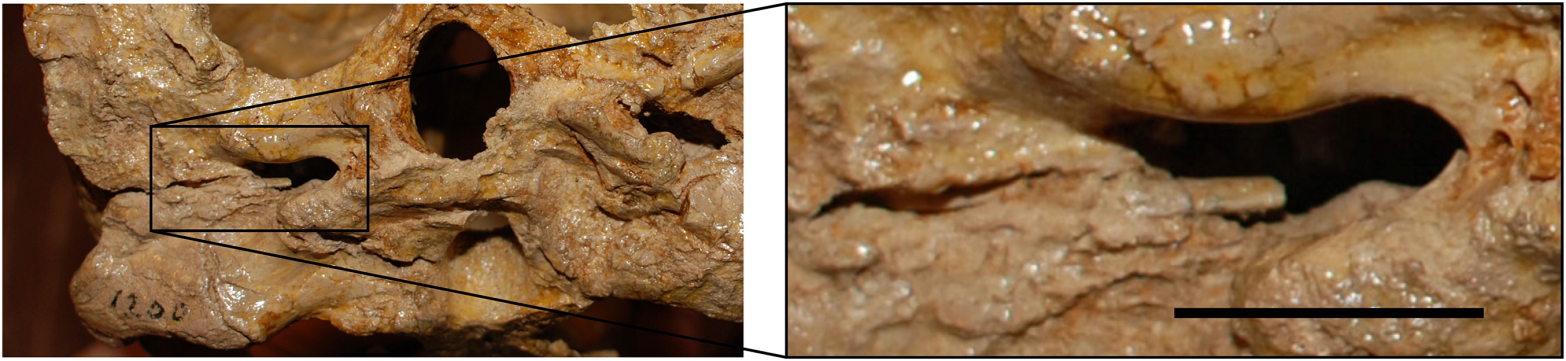
Posterior view of KUVP 1200, type specimen of *D. lowii*, highlighting the remains of the columella auris visible through the fenestra postotica. The bar marks 10 mm.

**Figure 36:**
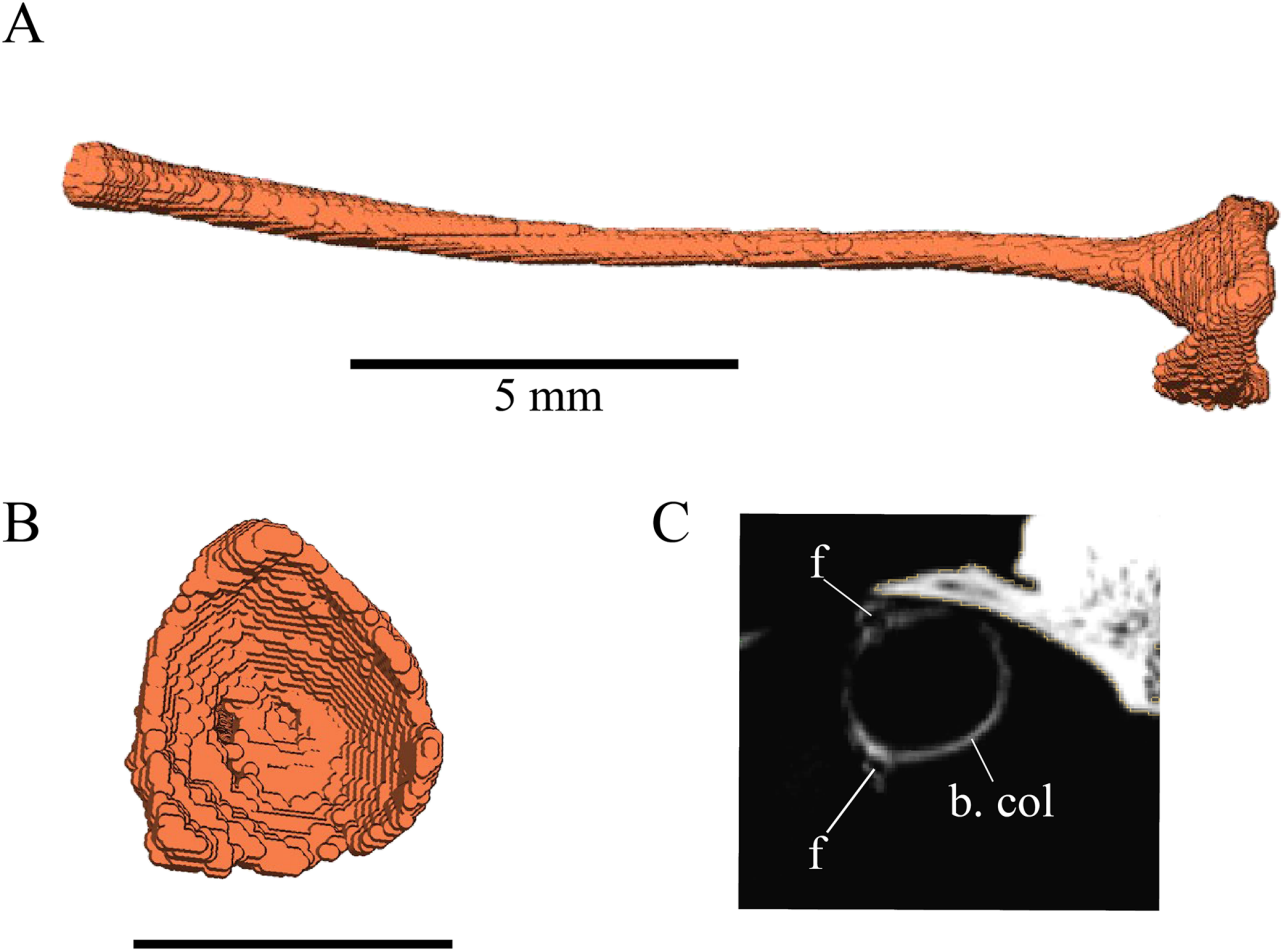
Columella of *Chelydra serpentina*. (A) “Side view”; (B) proximal view; and (C) cross section through the basis columellae in CT scan marking two foramina. The bar marks 10 mm, unless noted otherwise.

**Figure 37:**
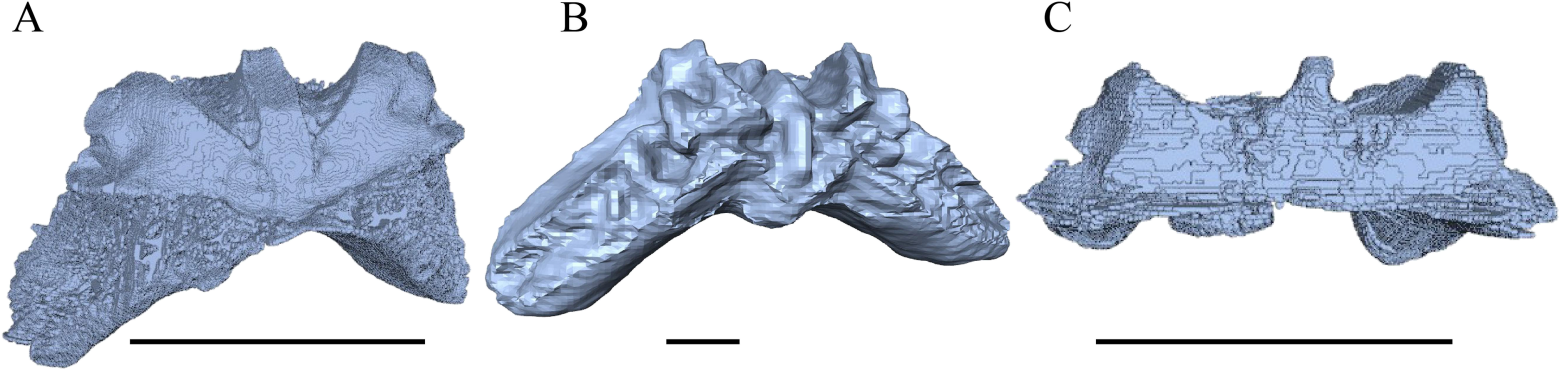
Basioccipital in anterolateral view. (A) *Eretmochelys imbricata*; (B) *Dermochelys coriacea*; and (C) *Chelydra serpentine*.

**Figure 38:**
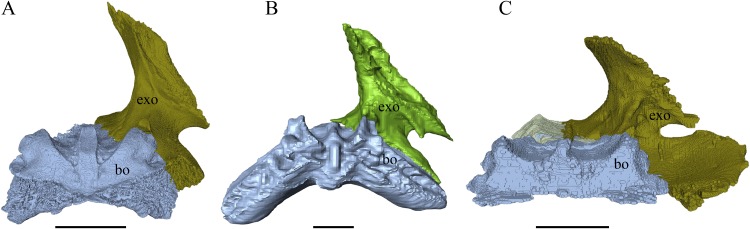
Exoccipital in medial view. (A) *Eretmochelys imbricata*; (B) *Dermochelys coriacea*; and (C) *Chelydra serpentina*.

**Figure 39:**
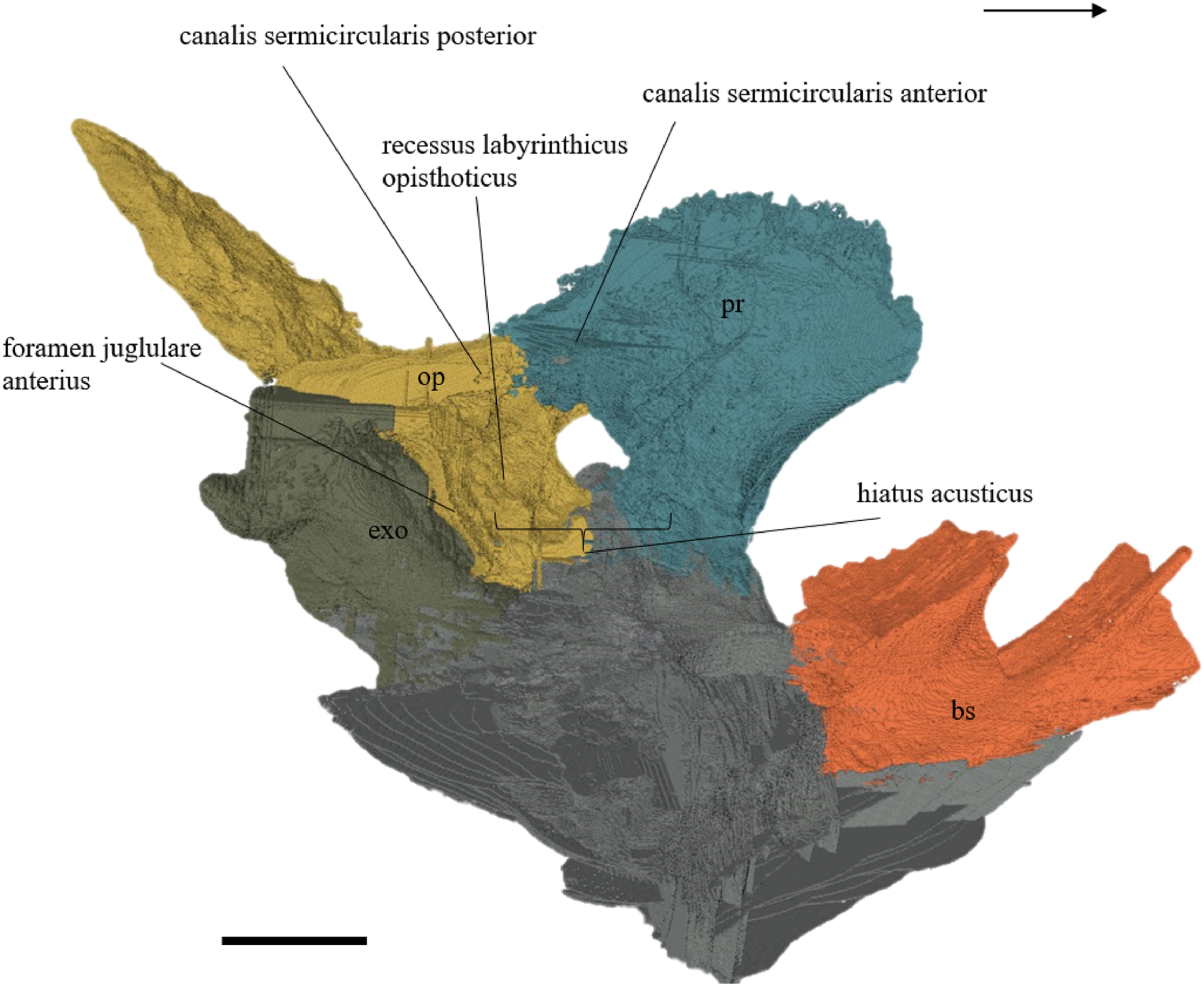
Exoccipital, opisthotic, prootic, basisphenoid, and an unidentifiable posterior portion of the basicranium of *D. lowii* in medial view. The bar marks 10 mm.

**Figure 40:**
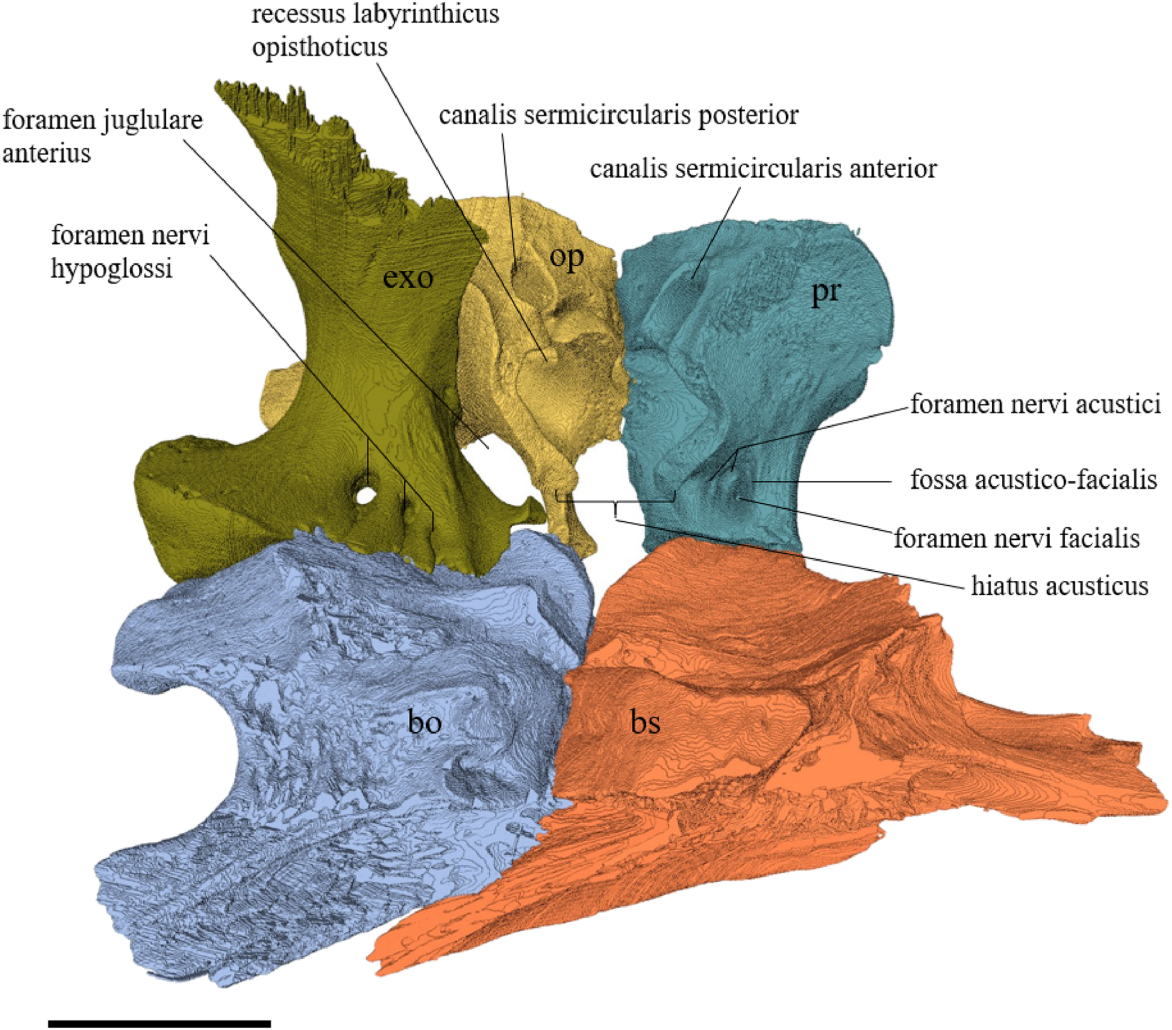
Exoccipital, opisthotic, prootic, and basisphenoid of *Eretmochelys imbricata* in medial view. The bar marks 10 mm.

**Figure 41:**
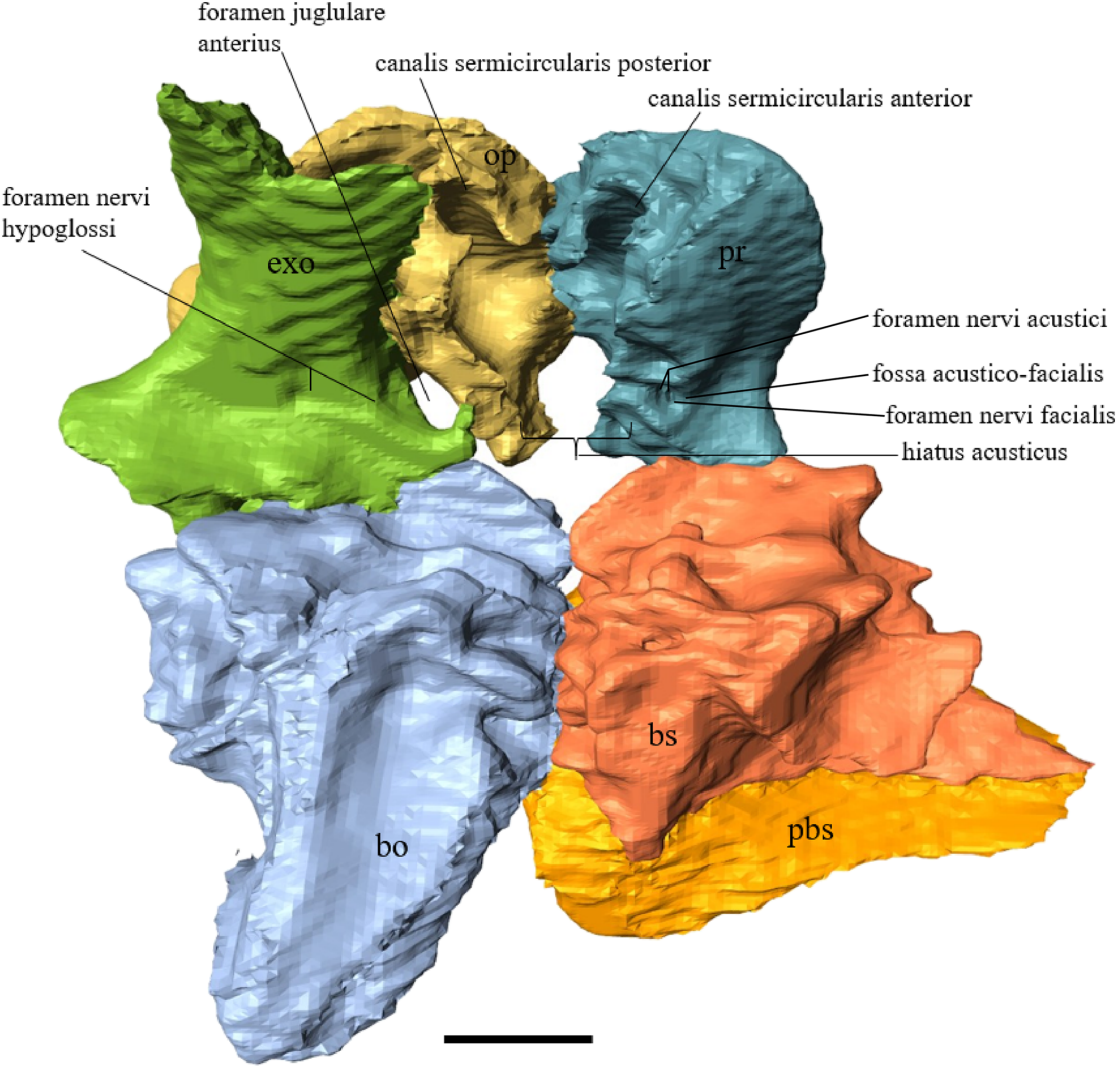
Exoccipital, opisthotic, prootic, basisphenoid, and parasphenoid of *Dermochelys coriacea* in medial view. The bar marks 10 mm.

**Figure 42:**
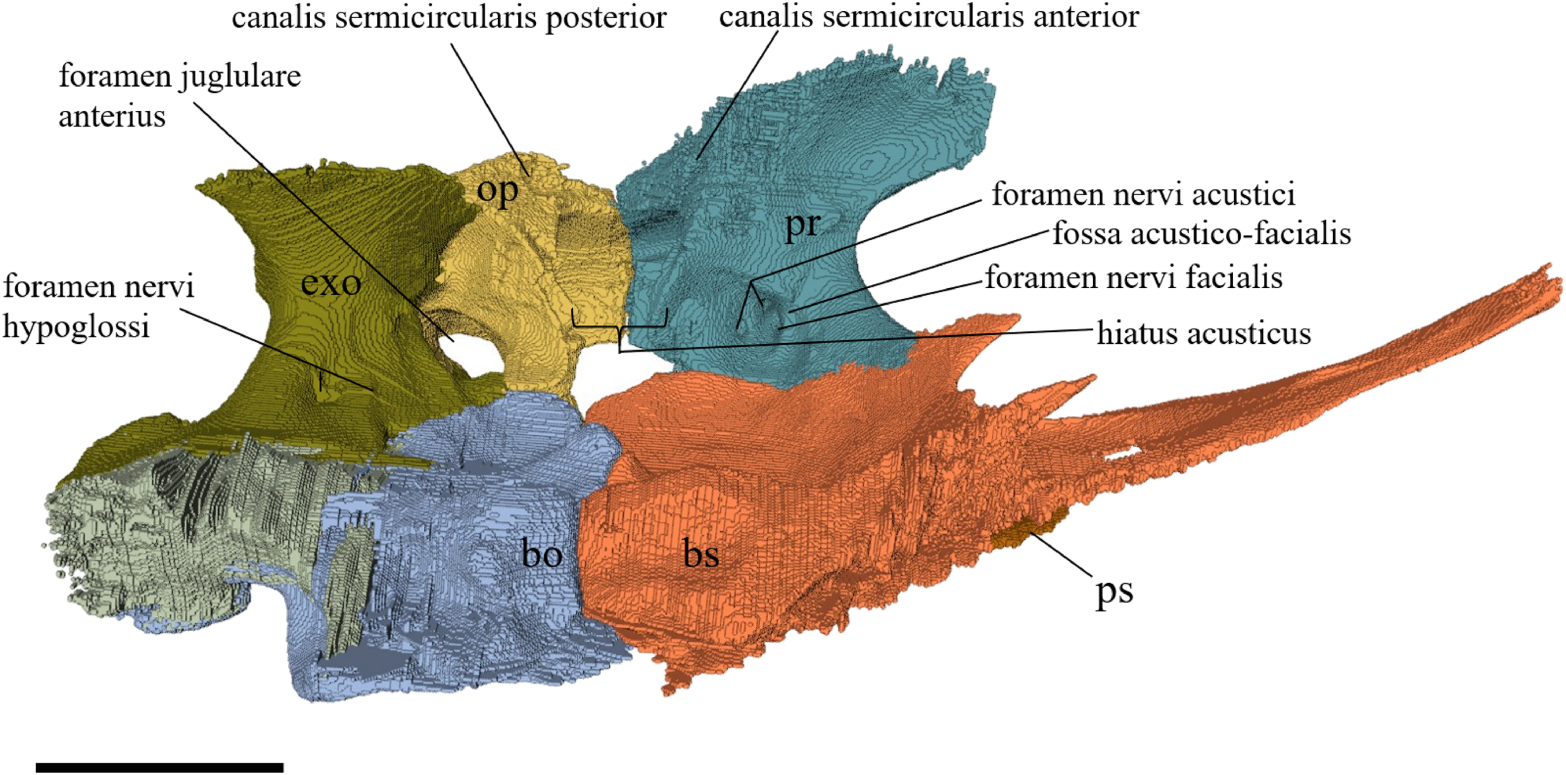
Exoccipital, opisthotic, prootic, basisphenoid, and parasphenoid of *Chelydra serpentina* in medial view. The bar marks 10 mm.

**Figure 43:**
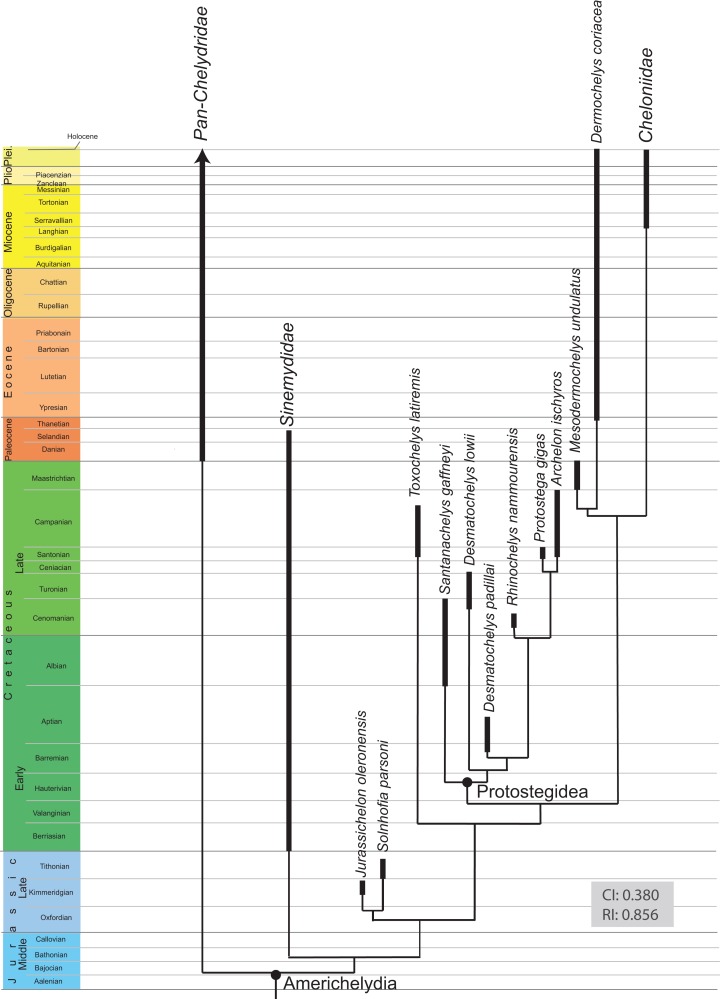
Time calibrated, most parsimonious tree (reduced version) resulting from the phylogenetic analysis. CI, consistency index; RI, retention index. The full tree is provided in Figs. S7.1–S7.3.

*Desmatochelys lowii—*The pterygoid of the available specimen of *D. lowii* is partially crushed, especially on the ventral side. In addition, it is difficult to determine the limits of the pterygoid bone. This is due to the fact that in the posteroventral part of the skull, the contacts are obscured by matrix. The posterior halves of the pterygoids are medially pierced by a vertical, 1 cm wide hole, which was produced using a mechanical drill to better mount the specimen. The two pterygoids contact each other medially in a parallel, interfingering suture. The processus pterygoideus is rather large, angular, and its lateral end is rather broad and tilted backward by 60°. The crista pterygoidea is rather high and posteriorly contributes to the foramen nervi trigemini. Anterodorsally, the crista contacts the epipterygoid in a narrow, interfingering, transverse suture, by which the epipterygoid covers the lateral side of the crista. Posterodorsally, the crista pterygoidea meets the processus inferior parietalis in a broad, strongly interfingering, vertically transverse suture, by which the parietal covers the lateral side of the crista. The crista pterygoidea participates medially in the sulcus cavernosus. On the lateral side of the crista pterygoidea, next to its anterior base, is a parasagittal groove with a posteriorly-situated foramen. This might be a foramen of the nervi vidiani, but as the canal leading to it cannot be traced, this cannot be ascertained with confidence. Another foramen can be observed behind the posterior base of the crista pterygoidea. Anteriorly, the pterygoid overlaps the palatine in an interfingering suture. The lateral side of the pterygoid’s posterolateral process meets the quadrate in a rounded, faintly interfingering suture, where the pterygoid slightly clasps the quadrate’s medial side of the processus articularis. Medially, the pterygoid meets the basisphenoid. The rostrum basisphenoidale lies on the contact between the two pterygoids. It is unclear how the rest of the basisphenoid is sutured to the pterygoid as the contact is strongly obscured by matrix. The posterolateral process of the pterygoid meets the prootic anterodorsally. I am not able to observe weather the pterygoid contacts the basioccipital or not. Unfortunately, even the likely contact between the pterygoid and the opisthotic cannot be determined with confidence.

*Eretmochelys imbricata—*The rather flat anterior part of the pterygoid forms two prominent spikes that connect with the palatine. The processus pterygoideus externus forms a small vertical flange. The bone is rutted by many grooves and pierced by many canals. Dorsally, several small crests, together with the prominent crista pterygoidea, shape the sulcus cavernosus, through which the vena capitis lateralis passes. Medially, a rather wide groove forms the lateral wall of the canalis caroticus lateralis. Ventrally, the pterygoid’s posterior half forms a trough, as its elongated edges ascend medially on the well-developed crest of the basisphenoid and laterally on the processus articularis of the quadrate. However, the pterygoid does not reach the margin of the condylus mandibularis. Laterally, beneath the crista pterygoidea, a short but distinct groove bears the processus pterygoideus of the quadrate and forms the fossa cartilaginis epipterygoidei. In the posteriormost part of the pterygoid, the foramen posterior canalis carotici interni is situated. The pterygoids meet each other medially in a parallel, moderately interfingering suture. Ventrally, their contact results in a very weak ridge. In approximately the middle of the ventral surface of the pterygoid, there are two foramina pharyngeale. Anteriorly, the pterygoid meets the palatine in a moderately interfingering suture, by which the palatine overlaps the pterygoid. The anteromedial spike of the pterygoid underlies the vomer medially. The crista pterygoidea connects the processus inferior parietalis in a vertically transverse, strongly interfingering suture, by which the parietal overlaps the lateral upper side of the crista pterygoidea. Posterolaterally, the pterygoid meets the quadrate in a moderately interfingering and slightly transverse suture by which the quadrate overlaps the pterygoid in a rather steep angle. The anteromedial wall of the pterygoid dorsally contacts the prootic in a rather loose, very short, parallel suture. The posterior end of the pterygoid is overlapped by the exoccipital in a rather strongly interfingering suture. In approximately the middle of the bone, the pterygoid meets the basisphenoid medially. In the anterior part of this suture, the rostrum basisphenoidale lies on the pterygoid. For the major, central part of the suture, the pterygoid meets the basisphenoid medially in a moderately interfingering contact, by which the pterygoid is somewhat bent toward the middle of the basisphenoid. Further anteriorly, the contact is rather straight but tilted, so the pterygoid faintly underlies the basisphenoid. In the posterior part of the suture, the basisphenoid clasps the pterygoid in a moderately interfingering suture. Behind the basisphenoid, the pterygoid is clasped by the basioccipital in a moderately interfingering suture. However, in the posterior part of the suture, the pterygoid overlaps the basisphenoid in a moderately interfingering suture. The pterygoid in this specimen does not contribute to the foramen palatinum posterius.

*Dermochelys coriacea—*The anterior margin of the anterior plate of the pterygoid is somewhat angular with an indentation in the middle. Laterally, a weak triangular structure follows the course of the margin of the fenestra subtemporalis. The dorsally dominant structures are the sulcus cavernosus and the crista pterygoidea, which has a horizontal foramen on the left side of the skull. Underneath the crista pterygoidea, on the lateral side, the fossa cartilaginis epipterygoidei is situated, which is dorsally bordered by a ridge. This ridge forms the ventral edge of the large foramen cavernosum. Ventrally, the posterolateral process forms a narrow subtriangular groove. The posterolateral process of the pterygoid does not reach the margin of the mandibular condyles. The anterior thirds of the anterior plate of the two pterygoid bones contact each other along the midline in a parallel and slightly interfingering suture. The length of this suture is shorter than the midline length of the basisphenoid. The posterior two-thirds of the pterygoid frame the basisphenoid, parasphenoid, and the basioccipital in a parallel manner. Approximately in the middle of the bone, the pterygoid faintly interfingers with the basisphenoid and ventrally overlaps the laterally extended margins of the parasphenoid in a faintly interfingering suture. The basioccipital contacts the pterygoid at the medioposterior surface of its posterior process in a moderately interfingering suture, by which the basioccipital somewhat clasps the pterygoid. Ventrally, the contact of the two pterygoids forms a fine groove. The pterygoid does not contact the exoccipital or the parietal. Instead, the top of the crista pterygoidea of the left pterygoid is connected to the ventral process of the prootic, in a somewhat vertically transverse, faintly interfingering suture. However, the latter contact does not occur on the right pterygoid. Anteriorly, the pterygoid meets the palatine in a transverse parallel contact. In the medial half of this suture the palatine notably overlaps the pterygoid, whereas in the lateral half the pterygoid faintly overlaps the palatine. Anteromedially, the pterygoid has a small contact with the ventral part of the posterior process of the vomer. Laterally, the pterygoid meets the quadrate’s medial surface in a slightly transverse, moderately interfingering suture, by which the quadrate overlaps the pterygoid in a steep angle. Additionally, the processus pterygoideus of the quadrate lies in the fossa cartilaginis epipterygoidei.

*Chelydra serpentina—*A striking feature is the generally flat ventral plate of the pterygoid of *C. serpentina*. A slight bending of the ventral surface only occurs at the processus pterygoideus externus and the posterior process. This results in the formation of a weak groove that contacts the condylus mandibularis. The anterior margin of the pterygoid medially forms a process, from which it forms a more or less transverse line, following the shape of the fishhook-like processus pterygoideus externus. The crista pterygoidea is triangular, just like the epipterygoid. From the anterolateral part of the crista pterygoidea springs a groove along the processus pterygoideus. This groove is an imprint of a presumably cartilaginous extension of the anterior process of the epipterygoid (see Discussion). The anterior process lies horizontally on the pterygoid and is sutured with it in a moderately interfingering manner. In the rest of the contact of these two bones, they meet in a smooth, vertically transverse suture, by which the epipterygoid overlaps the lateral side of the pterygoid’s crest. The sulcus cavernosus bears the foramen nervi vidiani in its anterior part and posteriorly forms a downward step, which constitutes the floor of the fenestra postotica. The two pterygoids meet each other at the midline in a parallel, strongly interfingering suture. Anteromedially, the pterygoid meets the vomer in a relatively broad and interfingering suture by which the pterygoid clasps the lateral side of the vomer’s posterior process. In the anteriomedial part of same indentation, the pterygoid clasps the palatine. Further laterally, the maxilla sneaks under the palatine into the indentation of the pterygoid. At this very point, the contact with the jugal on the dorsal surface begins and continuous until the end of the processus. Parallel to that, at the ventral side the maxilla connects the pterygoid until the end of the latter process. The type of suture of the pterygoid with the maxilla and the jugal is in both cases interfingering. The posterior halve of the pterygoid meets the basisphenoid. In the anterior part of this suture, the basisphenoid’s rostrum and its base lie on the pterygoid in a smooth suture, while ventrally to it, the parasphenoid meets the pterygoid in a smooth contact as well. Further posteriorly, the basisphenoid and the pterygoid meet in a parallel and moderately interfingering suture, while together they clasp the internal carotid canal. Posterodorsally, the pterygoid meets the prootic in a smooth to faintly interfingering suture. In the anterior part of the contact, the prootic overlaps the medial rim of the sulcus cavernosus, while further posteriorly the prootic connects both rims of the sulcus. In the posterior part, the prootic clasps the pterygoid’s crest, which separates the canalis cavernosus from the internal carotid canal. The type of the latter suture is rather smooth. Posterolaterally, the pterygoid is overlapped by the quadrate in a steep, faintly interfingering suture. Posteromedially, the pterygoid is clasped by the basioccipital’s ventrolaterally protruding parts in a faintly interfingering suture. In its posteriormost part, the pterygoid is overlapped by the exoccipital in a moderately interfingering suture. The pterygoid does not contact the opisthotic. The pterygoid in this specimen does not contribute to the foramen palatinum posterius.

Epipterygoid (Fig. 27)

*Desmatochelys lowii—*The epipterygoid of *D. lowii* is well preserved. It is a parasagittal, flat bone and approximately D-shaped. The epipterygoid participates to a minor part in the anterolateral wall of the sulcus cavernosus. The anterior margin of the epipterygoid is somewhat thicker than the rest of the bone and forms a bulge at the middle. The entire anterior margin is exposed. Posterodorsally, it contacts the processus inferior parietalis in a vertically transverse contact, by which the parietal slightly overlaps the lateral side of the epipterygoid’s posterodorsal margin. The posteroventral part of the epipterygoid meets the crista pterygoidea in a vertically transverse, moderately interfingering suture, by which the epipterygoid overlaps the lateral side of the pterygoid’s crista. The epipterygoid in this specimen does not participate in the formation of the foramen nervi trigemini.

*Eretmochelys imbricata—*A distinct epipterygoid in not preserved in the available specimen of *E. imbricata*. A slight thickening at the base of the descending process of the parietal, however, likely represents the fused remnants of this bone. In general, *E. imbricata* is known to possess an epipterygoid (Gaffney, 1979).

*Dermochelys coriacea—*The epipterygoid of *Dermochelys coriacea* is generally known to be absent (Nick, 1912).

*Chelydra serpentina—*The shape of the thin epipterygoid of *C. serpentina* is subtriangular and, as in *D. lowii*, directed parasagittally. The epipterygoid forms three processes that are somewhat curved. The vast majority of the anterior margin of the epipterygoid is in contact with the parietal and is exposed only for a short part at the base of its anterior process. The anterior process constitutes the anterolateral wall of the sulcus cavernosus and a part of it is bent anterolaterally and therefore lays on the dorsal surface of the processus pterygoideus externus. This anterolateral extension has an angular, flat ending. Anterior to this ending, a groove is formed by the pterygoid and jugal that may have held an embryonic precursor of the epipterygoid (see Discussion below). The part between the dorsal processus and the posterior processus of the epipterygoid shows a thickening, which forms a ridge. The posterior margin of the epipterygoid bone in this specimen participates in the formation of the foramen nervi trigemini. Dorsally, the epipterygoid meets the parietal in a slightly transverse suture, by which the processus inferior parietalis contacts the medial side of the epipterygoid. In the posterior part of the latter suture, where the epipterygoid’s thickened part is, the two bones strongly interfinger with each other. The epipterygoid connects with the entire dorsal margin of the crista pterygoidea in a transverse suture, by which the pterygoid connects the medial side of the epipterygoid.

Basisphenoid (Figs. 26, 28–30 and 39–42)

*Desmatochelys lowii—*In *D. lowii* only the anterior part can be discerned. The central and posterior parts of the basisphenoid were damaged when the skull was drilled for mounting. The ventral limits of this bone are uncertain, as the basicranium is partly crushed and its contacts are obscured by the matrix. The rostrum basisphenoidale is 1.5 cm long and consists of a backward-tilted dorsal plate and a ventrally attached, broad, rod-like structure. The rostrum ascends anteriorly by approximately 30° from the circular sella turcica, forms a sagittal groove, and ends on both sides of the lateral margin in a narrow rod. The left rod is much shorter and was presumably broken off or not fully ossified, whereas the right rod is almost four mm long. The two rods are connected by a transverse, rounded crest. From the middle of this crest, a short sagittal crest contacts the anterior end of the rostrum. The anterior end of the rostrum is drop-shaped and concave, with a prominent margin. Further, the surface of the rostrum is porous. The dorsum sellae of the basisphenoid is rather high and narrow. Anteroventrally, the basisphenoid contacts the pterygoids. The lateral side of the basisphenoid constitutes the medial wall of the sulcus cavernosus. Unfortunately, I cannot observe any carotid and nerve canals in this specimen as these internal structures are obscured by matrix.

*Eretmochelys imbricata—*The rostrum basisphenoidale is rod-like, rounded, and rather thick and has a two-cusped end. On the right side of the rostrum’s dorsal surface is a bony peak. The dorsum sellae is rater high and pierced by two rather prominent foramina nervi abducentis. On both sides of the rostrum is a groove that bears the lateral carotid artery and connects the sella turcica trough the short canalis caroticus interni. A canalis caroticus lateralis is not present in this specimen. The dorsal surface of the basisphenoid is concave with a crest in the middle. The rostrum basisphenoidale lies on the, along the midline elevated, suture of the two pterygoids. The rest of the basisphenoid meets the pterygoid on both lateral sides in a moderately interfingering, slightly transverse suture, by which the basisphenoid somewhat overlaps the pterygoid. Between these two bones the canalis caroticus internus is trapped. In the posterior part of the suture, the basisphenoid clasps the pterygoid. The ventral surface of the bone forms a prominent Y-shaped crest. The posteroventrolateral processes underlies the anterior part of the contact between the pterygoid and basioccipital. Posteriorly, the basisphenoid meets the basioccipital on two levels, each in a different type of suture. On the upper part, the basisphenoid meets the basioccipital in a blunt suture. In the lower part, the two bones meet in a strongly interfingering suture. The basisphenoid does not directly contact the prootic, but those two bones come each other very near in blunt surfaces, detached from each other by less than 0.5 mm.

*Dermochelys coriacea—*The basisphenoid of the *Dermochelys coriacea* is marked by its asymmetric morphology in the anterior part. The dorsum sellae is rather low. The right foramen abducentis is not fully ossified, respectively open. The rostrum basisphenoidale does not seem to be fully ossified as the bony parts end in the middle of the sella turcica, where basically two oblige walls descend from the left to the right, forming the canals of the internal carotid artery. The left oblique wall of the sella turcica is anteriorly elongated by 1.5 cm. This extension is dorsally covered by the pterygoids, while ventrally it meets the parasphenoid. At the dorsal part of the basisphenoid, two pits are separated by a crest along the midline, next to which two short processes ascend vertically. On the lateral sides of the dorsal part, the parallel lateral rims are bent upward. On their anterior halves, each of these rims contacts the prootic in a vertically transverse suture with a hollow center, which during lifetime presumably was filled with cartilage. Laterally and anteriorly, the basisphenoid contacts the pterygoid in an interfingering suture. Together the two bones form the canalis caroticus interni. The basisphenoid contacts the basioccipital posteriorly in a rather loose, parallel, and rough suture. Between the two bones, in the middle of the suture, a cavity is situated. Ventrally, the basisphenoid meets the parasphenoid in a concave, faintly interfingering suture.

*Chelydra serpentina—*The basisphenoid of *C. serpentina* is characterized by a rostrum basisphenoidale that is very flat, oval and remarkably long. In the middle of the rostrum is a short parasagittal crest, anteriorly to which two very thin canals pass the rostrum vertically. The two clinoid processes are very pointy and directed anteriorly. A cusp is formed medially to each clinoid process. The dorsum sellae is rather low. The foramen caroticum laterale is situated underneath the rostrum basisphenoidale and is smaller than the foramen anterius canalis carotici interni. The sella turcica is drop-shaped and rather wide. The widely spaced foramina anterius canalis carotici interni are situated at the posterolateral corners of the sella turcica. The ventral side of the basisphenoid is flat and shows two slightly concave structures in the posterior part. The dorsal part of the basisphenoid is rather deep, forming two concavities. Ventrally, the basisphenoid meets the parasphenoid. Ventrolaterally, it overlaps the pterygoid in a faintly interfingering suture. These two bones form the canalis caroticus internus. The cerebral carotid canal is situated within the basisphenoid. The rostrum basisphenoidale fully lies on the suture of the two pterygoids. Posteriorly, the basisphenoid meets the basioccipital in a mostly blunt, loose suture, with the exception of the lateroventral area. Laterodorsally, the basisphenoid meets the prootic in a blunt suture in the posterior two-thirds, while in the anterior third the two bones meet each other in a moderately interfingering suture.

Parasphenoid (Figs. 28–30 and 41)

*Desmatochelys lowii—*It is not possible to ascertain the presence of a formed or fused parasphenoid in the available specimen.

*Eretmochelys imbricata—*In this specimen the parasphenoid is absent.

*Dermochelys coriacea—*Among the analyzed specimens, the parasphenoid can only be clearly distinguished in the available specimen of *Dermochelys coriacea*. The parasphenoid in this specimen is subtriangular and floors the basisphenoid. The parasphenoid is larger than the basisphenoid and therefore covers it completely in ventral view. The anterior half of the bone is flat, whereas the posterior half forms is rounded M-shape in posterior view. Posteriorly, at the midline, the bone forms a concavity that forms a cone-shaped vacuity together with the overlying basisphenoid. This vacuity presumably forms a remnant of the notochord (Sheil, 2005). The lateral margins of the parasphenoid are overlapped by approximately one cm by the pterygoid, in a rather smooth suture. This suture thins out anteriorly, where the lower part of the short rostrum basisphenoidale lies on the tip of the parasphenoid. Posteriorly, the parasphenoid meets the basioccipital in a faintly interfingering transverse suture, by which the basioccipital overlaps the parasphenoid in a steep angle. This contact occurs only at the center part of the two bones, while the lateral processes of the parasphenoid and the tuberculum of the basioccipital are excluded from this contact. The posteroventral crest on the parasphenoid continues in the anteroventral part of the basioccipital.

*Chelydra serpentina—*In *C. serpentina* only the anterior part of what may constitute remnants of the parasphenoid can be distinguished. The anterior parts of these remnants form a flat, median slightly elevated, dense bony lamina, which at the front is not fused with the basisphenoid but covered by the pterygoids. The contact between the putative parasphenoid and the pterygoid is smooth. Toward the posterior end of the parasphenoid it is difficult to track the dorsal outline of the bone, as it seems to be fused with the basisphenoid.

Prootic (Figs. 31, 32 and 39–42)

*Desmatochelys lowii—*The prootic of the available specimen of *D. lowii* is rather well preserved. However, the limits of this bone are obscured by matrix. The dorsal exposure of the prootic is rather large. The prootic is situated remarkably elevated relative to the opisthotic. The foramen stapedio-temporale is situated above the level of the opisthotic. The prootic participates to about one-third to the margin of the foramen stapedio–temporale. Medially, the prootic meets the parietal in a vertically-transverse and moderately interfingering suture. The contact between the prootic and the parietal is rather long. Laterally, the prootic meets the quadrate in a parallel and faintly interfingering suture. Together the two bones form the processus trochlearis oticum, of which the majority is formed by the quadrate. Posteriorly, the prootic overlaps the opisthotic. Ventrally, the prootic meets the pterygoid. Posterodorsally, the prootic meets the supraoccipital in a parallel to vertically transverse suture, by which the prootic overlaps the lateral side of the supraoccipital’s lateral processes. However, no more details about the contact between the prootic and the opisthotic, pterygoid, and supraoccipital can be observed. Anteromedially, the prootic constitutes the posterior margin of the foramen nervi trigemini.

*Eretmochelys imbricata—*The dorsal exposure of the prootic is rather large. The lateral surface of the prootic is convex. The prootic forms half of the processus trochlearis oticum. The participating part forms a slightly forward leaning bulge with a rugose surface. Laterally, the prootic meets the quadrate in a moderately interfingering suture. In the anterior part this suture is parallel while the posterior part quadrate clasps the prootic in a curved suture. With its round posterodorsal margin, the anterior part the prootic overlaps the supraoccipital anterolateral margin. In the upper half of the latter contact, the suture is somewhat loose, while the lower half is marked by a moderate denticulation. In the posterior part of this suture, the two bones are separated from each other by less than one mm, a gap that was likely filled by cartilage. Posteriorly, the prootic contacts the opisthotic in a few spots in parallel, moderately interfingering, sutures, but these two bones are otherwise once again separated from each other by a modest gap.

*Dermochelys coriacea—*The dorsal exposure of the prootic in this specimen is rather large. With its dorsal margin the prootic connects the supraoccipital in a parallel, partly faintly interfingering, but mostly loose suture with a hollow center. Posteriorly, the prootic contacts the opisthotic in a parallel, moderately interfingering but rather loose suture. These two bones together form approximately 60% of the foramen stapedio-temporale. Laterally, the prootic meets the quadrate in a slightly interfingering, transverse suture, by which the prootic overlaps the quadrate’s medial margin in an angle of around 45°. The processus trochlearis oticum is not clearly recognizable in this specimen. With its ventromedial process, the prootic meets the pterygoid laterally and the basisphenoid medially. The contact between the prootic and the crista pterygoidea of the pterygoid is interfingering. The prootic and the basisphenoid only contact each other along their surfaces. A direct contact is absence for the cancellous portions of these bones. There is no triple junction where the prootic, opisthotic, and supraoccipital meet. Instead, this area is marked by a gap that was likely filled by cartilage in life. The prootic does not contact the parietal.

*Chelydra serpentina—*The dorsal exposure of the prootic in this specimen is rather large. The dorsolateral part of the prootic participates in the processus trochlearis oticum and forms a rather thin, rod-like process, which borders the anterior part of the foramen stapedio-temporale. From this foramen, a curved groove crosses the dorsal surface of the prootic toward the midline, with a bend to the anterodorsal part of the skull. This might be for the cervical artery. The prootic and the quadrate meet each other in a parallel, moderately-interfingering suture. The medial wall of the prootic has a small vacuity in the posterior part that is pierced by the foramen nervi facialis and two foramina nervi acustici. The upper part of the medial wall of the prootic has an oval recess that participates in the casing of the cranial cavity. Dorsomedioposteriorly, the prootic meets the supraoccipital anteriorly in a transverse suture where the prootic overlaps the supraoccipital in a moderately interfingering suture. In the posterior part of this suture, the two bones meet in in a parallel, smooth contact. Dorsomedioanteriorly, the prootic meets the parietal. In the anterior part of this suture, the prootic underlies the parietal in a faintly interfingering suture. In the posterior part of this suture, the prootic is clasped by the parietal in a smooth suture. Ventrally, the prootic meets the pterygoid at both borders of the sulcus cavernosus, as well as at the lateral and medial borders of the canalis carotici interni. The two bones meet in a faintly interfingering suture, by which the prootic overlaps and partly clasps the pterygoid. Posterodorsally, the prootic contacts the opisthotic in a parallel, smooth suture, which is rather angular along the transverse plane and resembles the letter T.

Opisthotic (Figs. 31–33 and 39–42)

*Desmatochelys lowii—*The contact of the opisthotic with the pterygoid is obscured. The tip of the processus paroccipitalis meets the squamosal in a parallel suture. Posteroventrally, the opisthotic lies on the exoccipital in a subhorizontal, parallel suture. Posterodorsally, the opisthotic meets the supraoccipital in a, as it seems, parallel suture. Anteriorly, the opisthotic underlies the prootic. Laterally, the opisthotic meets the quadrate in an anteriorly parallel suture, while in the middle of the contact the opisthotic underlies the quadrate. In the posterior part of this contact, the two bones again meet each other in a parallel suture. More details about this contact cannot be observed.

*Eretmochelys imbricata—*The opisthotic in this specimen does not participate in the formation of the foramen stapedio-temporale. The processus interfenestralis is rather narrow and does not reach the floor of the cavum acustico-jugulare. The transition part between the processus interfenestralis and the recessus labyrinthicus opisthoticus is pierced by a rather large foramen nervi glossopharyngei. The openings of the semicircular canals are rather large, as can be observed in *Dermochelys coriacea*, while the contrary applies for *C. serpentina*. The opisthotic meets the prootic anteriorly in an alternating mostly blunt to moderately interfingering suture. Anterolaterally, the opisthotic is overlapped by the quadrate. In the anterior part of this suture, the two bones meet in a loose and smooth suture, while in the posterior part, the contact becomes faintly interfingering and is much tighter and steeper. The opisthotic overlaps the lower part of the supraoccipital’s lateral process in a flat angle and a mostly blunt suture, that is only serrated a little toward its center. Posteromedially, the opisthotic overlaps the exoccipital in an alternating smooth to moderately interfingering suture. Posterolaterally, the opisthotic’s processus paroccipitalis meets the squamosal in a remarkably narrow and strongly interfingering suture. Dorsomedially, the opisthotic in this specimen meets the supraoccipital in a very loose, blunt suture, while the two bones do not contact each other directly. The opisthotic contributes posteriorly to the dorsolateral margin of the fenestra postotica, anterolaterally to the fenestra ovalis, anteromedially to the hiatus acusticus, and to the foramen jugulare anterius. The opisthotic in this specimen does not contact the pterygoid.

*Dermochelys coriacea—*The opisthotic in this specimen has a remarkably round processus paroccipitalis. The processus interfenestralis is short and does not reach the floor of the cavum acustico-jugulare. The foramen internum nervi glossopharyngei is rather small. The openings of the semicircular canals and the canals themselves are rather large and not fully defined by bone. Anteriorly, the opisthotic meets the prootic in a parallel, moderately interfingering suture, which is nearly C-shaped to angular and center-hollowed. Laterally, the opisthotic is clasped by the quadrate in a slightly transverse suture and slightly interfingering suture. Together with the latter two bones, the opisthotic contributes to the formation of the foramen stapedio-temporale to approximately one-third. Posteromedially, the opisthotic overlaps the exoccipital in a slightly interfingering suture and forms the dorsolateral margin of the fenestra postotica. Dorsomedially, the opisthotic meets the supraoccipital in a parallel, loose, and slightly interfingering suture. The opisthotic in this specimen neither meets the squamosal nor the pterygoid.

*Chelydra serpentina—*The opisthotic of *C. serpentina* is characterized by its angular morphology and its kinked processus paroccipitalis. The posterior half of the processus paroccipitalis is a flat wall, which ascends laterally and meets the squamosal and the posterior part of the quadrate. The opisthotic overlaps the squamosal in a transverse suture in the anterior part and faintly interfingers with squamosal in a parallel suture in the posterior part. The processus interfenestralis in this specimen is proportionally twice as broad as in the other analyzed specimens. Contrary to the other specimens, the processus interfenestralis of *Chelydra serpentina* does reach the floor of the cavum acustico-jugulare. The foramen jugulare-anterius is rather wide. The opisthotic contributes to a small part to the foramen jugulare posterius. The opisthotic meets the supraoccipital dorsomedially in a blunt suture. Anteromedially, the opisthotic contacts the prootic in a blunt suture. Anterolaterally, the opisthotic is overlapped by the quadrate in a moderately interfingering suture. Medially, the opisthotic overlaps the exoccipital in a moderately interfingering suture. The opisthotic of this specimen does not contribute to the formation of the foramen stapedio-temporale.

Quadrate (Figs. 20, 32 and 34)

*Desmatochelys lowii—*The contacts between the quadrate and its surrounding bones are obscured by matrix. The condylus mandibularis is remarkably broad. The medial half of the condylus is lower than the lateral half, as in *Dermochelys coriacea*. A processus epipterygoideus is not present in this specimen. The cavum tympani is remarkably shallow and oval. The incisura columellae auris is posteriorly open. The processus trochlearis oticum is mainly formed by the quadrate and is very prominent, yet narrow. A deep concavity is present between the processus trochlearis oticum and the lateral margin of the cavum tympani. The quadrate contributes to a small part of the foramen stapedio-temporale. The foramen stapedio-temporale is located on the dorsal side of the otic chamber and points posteriorly. Anteriorly, the quadrate meets the quadratojugal in a short suture. Dorsolaterally, the quadrate meets the squamosal in a varying suture. In the anteriormost part of the suture, the two bones met each other on a narrow, parallel suture. In the middle part of the contact, the quadrate overlaps the squamosal’s medioventral part. In the posterior part of this contact, the quadrate is dorsally and ventrally clasped by the squamosal. Anteromedially, the quadrate meets the prootic in a parallel suture. Posterolaterally, the quadrate meets the opisthotic. Anteroventrally, the quadrate meets the pterygoid in a somewhat parallel suture. The quadrate in this specimen does not contact the jugal.

*Eretmochelys imbricata—*The dorsal surface of the quadrate and the posterolateral side of the processus articularis are remarkably rugose in this specimen. The quadrate constitutes 50% of the rugose and rather prominent processus trochlearis oticum. The incisura columellae auris is posteriorly open. The foramina and canal of the chorda tympani are absent in this specimen, as is typical for *E. imbricata* in general (Gaffney, 1979). At about half way up of the anterior margin of the cavum tympani, a rod-like process of approximately 0.5 cm length ascends laterally in an angle of approximately 30°. This process contacts the quadratojugal’s posterior rim, which is bent toward the inside of the cavum tympani. At the contact surface, the quadratojugal forms a ridge that articulates with the quadrate process. The suture is smooth. Among the herein analyzed specimens, I could observe this feature only in *E. imbricata* on both sides of the skull. To my knowledge, this process has neither been described nor named so far and the functionality of this process is unknown to me. I therefore suggest a global examination of this feature among other turtles. The quadrate meets the prootic medially, above the canalis cavernosus. The anterior part of the suture between the two latter bones is strongly interfingering and transverse, by which the quadrate underlies the prootic in a steep angle. In the posterior part of this suture, the two bones are separated by the canalis stapedio-temporalis and shortly meet each other again at the posteriormost part, where they contact each other in a blunt suture. Posterior to this latter contact, the quadrate meets the opisthotic in a broad suture, which in the anterior part is parallel and smooth, while in the posterior part opisthotic underlies the quadrate in a strongly interfingering suture. Finally, at the very posterior end of the contact between the quadrate and the opisthotic, the two bones meet each other in a blunt transverse suture. The quadrate meets the squamosal dorsolaterally in a narrow and rather tightly interfingering suture, which continues onto the opisthotic, while the squamosal’s medial wall lies on the quadrate. Dorsoposteriorly, the quadrate and squamosal are not in direct contact but form matching surfaces, which are detached from each other by approximately one mm. Medially, beneath the level of the incisura columellae auris, the quadrate meets the pterygoid in a strongly interfingering and slightly transverse suture, by which the quadrate faintly overlaps the pterygoid. Anteromedially, the processus epipterygoideus of the quadrate lies in the pterygoids fossa cartilaginis epipterygoidei. The processus epipterygoideus is approximately four mm long, a little porous on its surface, rod like, and has a concave ending with a prominent circular margin. The quadrate is sparsely participating in the formation of the floor of the cavum acustico-jugulare, as the major part of this floor is formed by the pterygoid.

*Dermochelys coriacea—*The quadrate of *Dermochelys coriacea* is tilted backward by approximately 30°. As in *E. imbricata* the incisura columellae auris in this specimen is open posteriorly. A processus trochlearis oticum cannot be clearly identified. The condylus mandibularis has two articular condyles and an additional protrusion at the anterior part of the margin. The anterodorsal part of the cavum tympani is flattened. Anterodorsomedially, the quadrate contacts the prootic in a strongly interfingering, slightly transverse suture, by which the prootic overlaps the quadrate. The quadrate contributes to approximately one half of the margin of the foramen stapedio-temporale. Posterodorsally, the quadrate meets the opisthotic in a moderately interfingering, transverse suture where the quadrate overlaps the prootic. At the lower part of the medial side of the quadrate, the pterygoid connects the quadrate beneath the level of the incisura columellae auris in an interfingering, slightly transverse suture, where the quadrate overlaps the pterygoid. The processus epipterygoideus is remarkably short and rod like and its ending is oval and somewhat concave. Dorsolaterally, the quadrate meets the squamosal in a patchy, slightly interfingering suture, which is narrow in the anterior part and broad in the posterior part. The quadrate is sparsely participating in the formation of the floor of cavum acustico-jugulare, as the major part of this floor is formed by the pterygoid.

*Chelydra serpentina—*The quadrate of *C. serpentina* contributes to the formation of the cavum tympani and antrum postoticum. The anterior margin of the antrum postoticum is defined by a loop formed by the quadrate, but the posterior aspects are open, instead being formed by the squamosal. The posteroventral wall of the antrum postoticum internally forms a small parasagittal bulge, which continues into a more prominent crest, formed by the squamosal. The incisura columellae auris is closed posteriorly. The contact of the quadrate with itself forms a crenulated ridge that runs parallel to the incisura columellae auris and holds the Eustachian tube below. The small foramen chorda tympani inferius is situated below this ridge. A similar ridge is formed at the interior side as well. Half way up on the anterior part of the processus articularis, a one mm large foramen in situated. From this foramen, a canal ascends medially and branches off into a lower canal and an upper canal. The lower canal connects with the canalis cavernosus, while the upper canal branches off into two thinner canals, which connect to the canalis stapedio-temporalis. The foramina to the three corresponding canals can be observed on the medial face of the quadrate, as marked on the illustration. I am not aware of what these canals contained during life. Gaffney (1979) described and illustrated the medial face of the *C. serpentina*’s quadrate, but did not mention any structures like this. On the lateral side, along the anterior margin of the cavum tympani and on the upper anterior part of the processus articularis, the quadrate meets the quadratojugal in a strongly interfingering, parallel suture. A foramen is situated medially to the processus trochlearis oticum, on the anterior wall of the stapedial canal. The quadrate constitutes approximately 80% to the processus trochlearis oticum. On the dorsal surface of the quadrate, posterior to the processus trochlearis oticum, a rather deep recess is situated. The quadrate meets the prootic anteromedially in a moderately interfingering, transverse suture, where the prootic overlaps the quadrate. Posterior to this suture, the quadrate meets the opisthotic in a moderately interfingering and transverse suture, where the quadrate overlaps the opisthotic. However, the type of this suture changes at the very posterior end, where the quadrate is clasped by the processus paroccipitalis of the opisthotic. The processus epipterygoideus is rather long and flat and lies orthogonal to the narrow contact surface with the pterygoid. The suture between the quadrate and the pterygoid is moderately interfingering and transverse. The quadrate is sparsely participating in the formation of the floor of cavum acustico-jugulare, as the major part of this floor is formed by the pterygoid.

Columella auris (Figs. 35 and 36)

The columella auris is not preserved for the available specimens of *E. imbricata* and *Dermochelys coriacea*.

*Desmatochelys lowii—*In KUVP 1200, a slightly flattened, partly crushed rod is situated in the incisura columellae auris that protrudes from the fenestra postotica. I conclude that this structure is likely the columella auris from which the basis columellae has broken off. The columella can otherwise only be shown to have an oval cross section.

*Chelydra serpentina*—The columella of *C. serpentina* consists of a two cm long, slightly curved rod and a basis columellae with a diameter of around four mm. The basis columellae is a subtriangular hemisphere. In the frontal view, one can observe two pointed corners and a more rounded corner. Both of the pointy corners bear a tiny canal, which can be seen best in the CT scan. In side view, the margin between the two pointy corners is extended backward and forms a collar-like structure.

Basioccipital (Figs. 26, 37 and 39–43)

*Desmatochelys lowii—*I was not able to determine the limits of the basioccipital in the available specimen of *D. lowii* due to crushing and a lack of contrast between bone and matrix.

*Eretmochelys imbricata—*The basioccipital of *E. imbricata* consists of a heart shaped dorsal surface, laterally descending processes that end as the tuberculi basalis and a posteriorly descending, subtriangular and rather wide process that forms the ventral third of the condylus occipitalis. The basioccipital in this specimen has a rather prominent crista dorsalis basioccipitalis with an oval, concave basis tuberculi basalis. The lateral margins of the dorsal surface are anteriorly elevated and wide and form the posteroventral margin of the fenestra ovalis. Further, the dorsal surface of the basioccipital is marked posterolaterally by many small concavities, which are part of the partly moderately interfingering and partly loose suture between the exoccipital and the basioccipital. Also, on the dorsal part of the posterolateral descending processes, the basioccipital meets the exoccipital in a strongly interfingering suture. On the posterior process of the basioccipital, the two bones meet in a parallel, smooth suture, while at the lateral edge of the basioccipital process, the two bones are strongly interfingering. Laterally, the basioccipital clasps the pterygoid in a moderately interfingering suture. Ventrally, the basioccipital meets the basisphenoid’s ventral crest in a blunt suture. The basioccipital in this specimen does not possess a ventral tubercle.

*Dermochelys coriacea—*As in *E. imbricata*, the ventrolaterally descending processes of *Dermochelys coriacea* are rather prominent and form a C-shaped concavity between each other. Further, on the ventral side of the basioccipital of this specimen, along the midline, is one ventral tubercle. On both sides of the crista dorsalis basioccipitalis is a deep trough each, which laterally end in a rather high wall that contacts the exoccipital. The posterior end of the basioccipital’s process that contributes to the condylus occipitalis, shows a rugose surface as it is not completely ossified. The basioccipital in this specimen is overlapped by the exoccipital posterolaterally in a moderately interfingering suture. The contact between these two latter bones is situated mostly on the dorsal surface of the posterior half of the ventrolateral processes. The dorsal surface of the anterior half of this process floors a part of the cavum acustico-jugulare. The anterior surface of the process contacts the pterygoid in a parallel and strongly interfingering suture. Anteriorly, the basioccipital contacts the basisphenoid in a rather loose, parallel, and moderately interfingering suture. In the middle part of the suture, these two bones do not contact each other, as there is a concave space between the basis tuberculi basalis and the posteromedial part of the basisphenoid. The lowermost part of the basioccipital shortly overlaps the parasphenoid’s posterior part in a wavy, moderately interfingering suture.

*Chelydra serpentina—*The basioccipital of *C. serpentina* differs from the other specimens through its rather flat ventral side. The crista dorsalis basioccipitalis is rather short and the tuberculum high, thereby resembling a knob. The limits of the posterior half of this basioccipital are difficult to determine as this bone is fused with the exoccipital. This condition is common in adult specimens, as those sutures fuse with age (Siebenrock, 1897). However, it can be said that the posterior process of the basioccipital is dorsally covered by the two exoccipitals. Anteriorly the basioccipital meets the basisphenoid in a mostly blunt suture. Exceptions can be observed in the area directly next to the basis tuberculi basalis, where these two bones interfinger moderately with each other in the uppermost part of the contact and in the most lateral part of the basioccipital, where the two bones interfinger moderately with each other in the lowermost part of the contact. Posteroventrally, the basioccipital meets the pterygoid in a transverse suture. In the posterior part of this moderately interfingering contact, the basioccipital overlaps the pterygoid, while in the anterior part, the pterygoid overlaps the basioccipital.

Exoccipital (Figs. 38–43)

*Desmatochelys lowii—*In *D. lowii*, I was not able to determine the limits of the exoccipital, as it is nearly impossible to recognize any definite structures, due to crushing and a lack of contrast between the bone and the matrix. Only the posterolateral part of the exoccipital is rather well preserved and one can clearly observe two foramina nervi hypoglossi. The external limits of the exoccipital are faintly visible in this part of the skull. Dorsally, the exoccipital meets the supraoccipital. Although not certain, it seems that the two exoccipitals do not contact each other dorsally. The foramen jugulare posterius is confluent with the rest of the fenestra postotica. The upper lateral process of the exoccipital is rather long and underlies the opisthotic. This process forms the majority of the dorsal margin of the fenestra postotica.

*Eretmochelys imbricata—*The exoccipital in this specimen has three foramina nervi hypoglossi on the inside side of the skull that connect to two foramina nervi hypoglossi on the outside of the skull in both sides. The bone between this foramina forms cone-shaped structures. Anteriorly, the exoccipital has a short, forward pointing process, with a backward oriented distal flange. This process does not reach the opisthotic, therefore the foramen jugulare anterius stays anteroventrally open. The exoccipitals in this specimen do not contact each other dorsally to the foramen magnum. Dorsally, the exoccipital meets the supraoccipital in a vertically transverse, strongly interfingering suture and in the posteriormost part of this suture, the exoccipital is dorsally clasped by the same bone. Ventrally, the exoccipital meets the basioccipital in a strongly interfingering suture, except for at the condylus occipitalis, where the two bones meet each other in a blunt suture and are only interfingering at the lateral edge of the basioccipital’s posterior process. Laterally, the exoccipital is overlapped by the opisthotic in an alternating blunt to interfingering suture. Ventrolaterally, the exoccipital overlaps the posterior part of the roof of the canalis caroticus interni, which is formed by the pterygoid. The suture between those two bones is strongly interfingering. The exoccipital forms the medial margin of the fenestra postotica. The posterior process of the exoccipital participates in the formation of the condylus occipitalis. The foramen jugulare posterius is confluent with the rest of the fenestra postotica.

*Dermochelys coriacea—*The condylus of the exoccipital is not fully ossified. The exoccipitals do not contact each other dorsally to the foramen magnum. The exoccipital is dorsally overlapped by the supraoccipital in a moderately interfingering suture. Laterally, the exoccipital contacts the opisthotic in a moderately interfingering, transverse suture, by which the opisthotic overlaps the exoccipital. Ventrally, the exoccipital overlaps the basioccipital in a moderately interfingering suture. On the medial side, two foramen nervi hypoglossi can be observed, from which the posterior foramen is remarkably wider than the anterior. The anterior process of the exoccipital does not contact the opisthotic and the foramen jugulare anterius is anteroventrally open. The exoccipital in this specimen does not contact the pterygoid, as the two bones are separated by the basioccipital. The foramen jugulare posterius is partly ossified in this specimen.

*Chelydra serpentina—*The exoccipital in this specimen is characterized by its large anterolateral plate, which is subhorizontal, slightly tilted anteroventrally and floors the recessus scalae tympani. This process anteriorly connects to the pterygoid in a moderately interfingering and transverse suture, by which the exoccipital overlaps the pterygoid. The exoccipitals in this specimen do not contact each other dorsally to the foramen magnum. However, they are fused with each other ventromedially. The exoccipital is dorsally overlapped by the supraoccipital in a strongly interfingering suture. The exoccipital is pierced by two foramen nervi hypoglossi which are equal in size. Laterally, the exoccipital meets the opisthotic in a strongly interfingering, transverse suture, where the opisthotic overlaps the exoccipital. The processus interfenestralis is situated at the suture between these two bones. Ventromedially, the exoccipital meets the basioccipital. However, the type of suture cannot be examined between those two bones, as they are fused. This condition is typical in adult specimens (Siebenrock, 1897). The exoccipital forms the majority of the foramen jugulare posterius, with a small contribution of the opisthotic. In contrast to the other species, the anterior process of the exoccipital of *C. serpentina* does contact the opisthotic, which leads to the anteroventral closure of the foramen jugulare anterius.

Supraoccipital (Fig. 32)

*Desmatochelys lowii—*The supraoccipital of *D. lowii* has a broken crest. What remains protrudes 1.5 cm relative to the foramen magnum. The limits of the supraoccipital are mostly obscured by matrix. Dorsally, the supraoccipital meets the parietal. Ventrolaterally the supraoccipital meets the opisthotic in a parallel contact and the prootic in a partly vertically transverse, but mostly parallel contact. The contact between the supraoccipital and the exoccipital is unclear.

*Eretmochelys imbricata—*The dorsal exposure of the supraoccipital is restricted to the posterior half of its crista. The crista supraoccipitalis is rather long, protruding three cm posterior to the foramen magnum. The protrusion of the crista relative to the foramen magnum is longer than the rest of the supraoccipital bone. At the point where the crista appears in the dorsal view, it forms a horizontal, rhomboidal expansion of 1.5 cm. From the crista, the supraoccipital bone widens continuously. Dorsolaterally, three quarters of the supraoccipital are covered by the parietal in a rather strongly interfingering suture. Posteroventrally, the supraoccipital overlaps the exoccipital in a strongly interfingering suture. At the very posterior end of this suture, the supraoccipital clasps the dorsal crest of the exoccipital. Posterolaterally, the supraoccipital meets the opisthotic in a remarkably blunt and rounded suture. Anteroventrally, the supraoccipital contacts the prootic in a transverse suture where the prootic overlaps the supraoccipital’s anterolateral margin. In the anterior part of this suture, the upper half of the contact is somewhat loose, while the lower half shows strong denticulations. The two flat lateral processes of the supraoccipital are ventrally wider, form the recessus labyrinthicus supraoccipitalis, as they participate in the formation of the anterior and posterior semicircular canals of the inner ear. Ventromedially, this processes each form the upper margin of the hiatus acusticus. In the middle of the upper margin is a notch, the foramen aquaducti vestibuli. The supraoccipital constitutes the uppermost part of the foramen magnum.

*Dermochelys coriacea—*The crista supraoccipitalis in this specimen is rather short and massive and the dorsal exposure of the crista reaches only 0.5 cm. The shape of the crista is somewhat asymmetric, but it does not form any horizontal crests. The protrusion of the crista relative to the foramen magnum is slightly shorter than the rest of the supraoccipital bone. The anteriormost upper part of the supraoccipital forms a slightly backward tilted, arched end. Dorsally, the supraoccipital is overlapped by the parietal in a slightly transverse and moderately interfingering suture. Posteroventrally, the supraoccipital overlaps the exoccipital in an interfingering and rather tight suture. Anteroventrally, the supraoccipital’s lateral processes each contact a prootic in a transverse and slightly interfingering suture, by which the prootic overlaps the supraoccipital’s lateroventral margins. Much of the bones surrounding the inner ear and poorly ossified and the recessus labyrinthicus supraoccipitalis is therefore poorly defined. Due to many cavities, the contact between the supraoccipital and the opisthotic is rather fragmentary but can be classified as moderately interfingering and transverse, as the supraoccipital overlaps the opisthotic. The supraoccipital forms the upper margin of the hiatus acusticus and a notch, the foramen aquaducti vestibuli, in the middle of this very margin.

*Chelydra serpentina—*The supraoccipital in this specimen is more prominent than in the other herein analyzed specimens due to its fully exposed crest. However, except for the crest, the supraoccipital bone itself is not exposed on the dorsal skull roof. The protrusion of the crista relative to the foramen magnum is longer than the rest of the supraoccipital bone. The bone in the center of the crista supraoccipitalis is remarkably thin, as well as the ventromedial walls of the lateral processes, which are even partly broken. Anterodorsally, the supraoccipital contacts the parietal in a strongly interfingering and transverse suture, where the parietal overlaps the supraoccipital. The anteriormost upper part of the supraoccipital forms a vertically cut, arched end. Posteroventrally, the supraoccipital overlaps the exoccipital in a strongly interfingering suture. Posterolateroventrally, the supraoccipital meets the opisthotic in a blunt suture. Anteroventrally, the supraoccipital contacts the prootic in a transverse suture, where the posterior half of the two bones contact each other in a blunt suture, which changes in the anterior half into an interfingering suture. As in the other specimens, the supraoccipital forms the upper margin of the hiatus acusticus and a notch, the foramen aquaducti vestibuli, in the middle of this margin.

### Carotid circulation and vidian nerve

*Desmatochelys lowii—*Unfortunately, due to the bad condition of the basicranium in the available specimen, the course of the carotid canals cannot be examined. Although the sella turcica is rather well preserved, no foramina can be observed in its vicinity. Nevertheless, a foramen is present in the center of the dorsal side of the processus pterygoideus externus, which most probably represents an exit of the vidian canal.

*Eretmochelys imbricata—*The internal carotid artery enters the skull through the posterior foramen of the internal carotid canal, which is fully surrounded by the pterygoid. The posterior half of the canal is formed by the pterygoid, while the basisphenoid medially participates in the formation of the anterior half of the canal. At the level of the basioccipital–basisphenoid contact, the internal carotid canal shortly opens to the floor of the fenestra ovalis. Further anteriorly, the internal carotid canal gives off a canal, the “foramen” pro ramo nervi vidiani, which connects to the sulcus cavernosus and at least transmits the vidian nerve (palatine branch of the facial nerve VII) (Albrecht, 1967). Finally, the large internal carotid canal opens into the anterior, lower situated part of the sulcus cavernosus, where it branches into the thick palatine artery and the thin cerebral artery, which enters the sella turcica medially through the canalis carotici cerebralis. A developed palatine carotid canal is not present in this specimen. Ventrally, each internal carotid canal is connected to two carotico-pharyngeal canals, which may have contained small branches of the vidian nerve.

*Dermochelys coriacea—*The vast majority of the internal carotid canal is not ossified in this specimen. The canal is briefly enclosed between the basisphenoid and the pterygoid on the right side of the basisphenoid only. However, on the anterior half of the basisphenoid, two deep, asymmetric grooves give indication for the further path of the internal carotid artery. The carotid artery most probably enters the skull through the fenestra postotica. The course of the cerebral carotid artery and the vidian nerve cannot be observed in this specimen.

*Chelydra serpentina—*The internal carotid artery enters the skull through the fenestra postotica and only later enters a defined internal carotid canal—this can be interpreted as the posterior foramen of the internal carotid canal. The posterior part of this canal is formed by the prootic and the pterygoid. Further anteriorly, where the canal comes closer to the midline, the basisphenoid participates in the formation of the canal as well. The canal gives off a branch that connects to the sulcus cavernosus, the “foramen” pro ramo nervi vidiani. Shortly afterward, the canal of the vidian nerve splits off the internal carotid canal, crosses the pterygoid, and ends in the foramen nervi vidiani at the level of the anterior part of the basisphenoid’s rostrum. Anteriorly to the latter split, the internal carotid canal splits into the thin palatine canal, that crosses the pterygoid and exists in the sulcus cavernosus, and the cerebral canal, which crosses the basisphenoid and exists at the sella turcica.

## Results

### Additional characters

I herein expand the character/taxon matrix of Cadena & Parham (2015) through the addition of the seven characters listed in Table 3. Character 62 pertains to the participation of the epipterygoid to the foramen nervi trigemini. This character had first been developed by Meylan (1987) to investigate trionychian relationships, but has not yet been applied to a global matrix. Character 88 addresses the participation of the opisthotic in the formation of the foramen stapedio-temporale. This character had first been utilized by Brinkman & Nicholls (1991) to reconstruct relationships among baenids, but also has not yet been applied to more global questions. As far as I am aware, characters 32, 33, 64, 87, and 92 are new and are therefore explained in greater detail. Character 32 addresses the presence of a hemispherical depression in the middle of the ventral side of the skull roof. It is typically formed by varying contributions from the postorbital, frontal, and parietal (Fig. 17). This recess can be observed in *D. lowii*, where it is situated in the middle of the anterior half of the postorbital’s ventral side and in *C. serpentina*, where the recess is more strongly pronounced than in *D. lowii* and situated at the triple junction of the postorbital, frontal, and parietal. Such a recess is not present in the other two analyzed specimens. Character 33 describes the notch in the posterior rim of the orbit (Fig. 18). When present, this notch is either located at the top of the posterior margin of the orbit (as in *E. imbricata* and *Dermochelys coriacea*) or in the middle of the posterior margin of the orbit (as in *C. serpentina* and *D. lowii*). Character 64 pertains to the length of the suture between the prootic and the parietal (Fig. 32). In the examined specimens, this contact is either long, constituting almost half of the dorsomedial margin of the prootic, or remarkably short, less than 5% of the dorsomedial margin of the prootic, to absent. Character 87 addresses variation to the morphology of the dorsomedial process of the prootic in medial view (Figs. 39–42). Two states are observed: the prootic either lacks a dorsomedial extension while having a similar height as the opisthotic or the prootic forms a dorsal medial process that ascends far beyond the level of the opisthotic. This character cannot be applied to the available specimen of *D. lowii*, because the limits of the two corresponding bones are not clear. Finally, character 92 pertains to the presence of the processus epipterygoideus, which is absent in *D. lowii*, but present in the sampled specimens of *E. imbricata, Dermochelys coriacea*, and *C. serpentina*. The newly included characters were only scored for the four taxa studied herein, because internal views are not yet available for the taxa included in the matrix of Cadena & Parham (2015).

**Table 3:** Description and scoring of the additional characters 32, 33, 62, 64, 87, 88, and 92.

Character	Bone	Character description	*Desmatochelys lowii*	*Eretmochelys imbricata*	*Dermochelys coriacea*	*Chelydra serpentina*
32	Postorbital	Recess on the ventral side of the dorsal part of the postorbital. 0 = absent; 1 = present	1	0	0	1
33		Position of notch in the posterior margin of the orbit. 0 = at the top of the orbit; 1 = at the mid level	1	0	0	1
62	Epipterygoid	Participation of the epipterygoid to the foramen nervi trigemini. 0 = absent; 1 = present	0	–	–	1
64	Prootic	Extent of the suture with the parietal. 0 = very short or absent; 1 = long	1	0	0	1
87		Dorsomedial process of the prootic in medial view. 0 = absent, prootic has a similar height to the opisthotic; 1 = present, prootic process significantly higher then opisthotic	–	0	0	1
88	Opisthotic	Participation of the opisthotic to the formation of the foramen stapedio-temporale. 0 = absent; 1 = present	1	0	1	0
92	Quadrate	Processus epipterygoideus. 0 = absent; 1 = present	0	1	1	1

### Update of the coding of *D. lowii* of Cadena & Parham (2015)

The scoring of the following list of characters was updated relative to that of Cadena & Parham (2015) based on new observations obtained as part of this study. As Cadena & Parham (2015) relied on previously available literature that only imperfectly documented KU VP1200, all noted differences can be viewed as improvements, not observations of polymorphism. For full descriptions of the following characters, citations and comments of the authors, see Cadena & Parham (2015). The numbers in brackets represent the character numbers of Cadena & Parham, while the numbers without brackets follow the order of the updated matrix in the study herein, which includes seven additional characters.

Character 8 (8), development of prefrontal scutes: previous coding: 0; new: ?
Stages: 0 = one pair; 1 = two pairs or more
Comment: No cranial scutes can be observed in the available specimen of *D. lowii*. This therefore cannot be scored.
Character 18 (18), presence of pineal foramen: previous coding: 1; new: 0
Stages: 0 = absent; 1 = present
Comment: A pineal foramen is not present in the available specimen of *D. lowii*. Instead, the relevant part of the skull is damaged, as can be seen by reference to historic literature (see Discussion).
Character 21 (21), jugal-quadrate contact: previous coding: 1; new: 0
Stages: 0 = absent; 1 = present, quadratojugal does not contribute to lower temporal margin.
Comment: The jugal of *D. lowii* is rather short and separated from the quadrate by the quadratojugal (see Figs. 5 and 18).
Character 78 (74), presence of median pterygoid ridge: previous coding: 1; new: 0
Stages: 0 = incipient to absent; 1 = present, ridge spans nearly the full length of the pteygoids, sometimes reaching the most posterior portion of the vomer. The medial ridge is produced by the extremely concave posterolateral portions of both pterygoids.
Comment: A median pterygoid ridge cannot be observed in *D. lowii*. The relevant area instead is flat.
Character 84 (80), development of basioccipital tubercles: previous coding: 1; new: 0
Stages: 0 = with two or one ventral tubercle; 1 = tubercle absent.
Comment: In the available specimen of *D. lowii*, KUVP 1200, the basicranium is damaged. Therefore, the morphology of the basioccipital cannot be discerned. However, illustrations of the specimen MNA V4516 (Elliot, Irby & Hutchison, 1997) of *D. lowii* show well developed tubercula on the basioccipital.
Character 90 (86), morphology of rostrum basisphenoidale: previous coding: 0; new: 1
Stages: 0 = flat; 1 = rod-like, thick and rounded.
Comment: The rostrum basisphenoidale of *D. lowii* is rod-like, and drop-shaped in cross section. It is more than twice as high as wide at its maximum height.
Character 96 (90), height of dorsum sellae: previous coding: 0; new: 1
Stages: 0 = low; 1 = high.
Comment: The dorsum sellae of *D. lowii* is notably high, about four times higher than the vertical protrusion of the sella turcica relative to the floor of the sulcus cavernosus.
Character 105 (98), ventral covering of foramen nervi hypoglossi: previous coding: 0; new: 2
Stages: 0 = exposed in ventral view; 1 = covered in ventral view by an extension of the pterygoid and the basioccipital; 2 = covered in ventral view by an extension of the basioccipital.
Comment: The foramen nervi hypoglossi are not visible in ventral view, as they are fully covered by the basioccipital.
Character 107 (100), formation of foramen posterius canalis carotici interni: previous coding: 1; new: ?
Stages: 0 = absent; 1 = formed by pterygoid; 2 = formed by pterygoid and basisphenoid halfway along the basisphenoid-pterygoid suture; 3 = formed by prootic, prootic and basisphenoid, or prootic and pterygoid; 4 = formed by basisphenoid only.
Comment: The foramen posterius canalis carotici interni, if present, is not visible in the available specimen of *D. lowii*.
Character 109 (102), size of the fenestra perilymphatica: previous coding: 0; new: ?
Stages: 0 = large; 1 = reduced in size to that of a small foramen.
Comment: The fenestra perilymphatica cannot be identified in the available specimen of *Desmatochelys lowii*.

### Phenetic study

The results of the phenetic study, that bases on bone contacts, are provided in Table 4 in the form of a similarity matrix. The greatest similarity with 51% is observed between the extant marine turtles *D. coriacea* and *E. imbricata*. The smallest among of similarity, by contrast, is observed between *D. lowii* and *C. serpentina*. In all cases, *D. lowii* shows the least amount of similarity relative to the other three species.

**Table 4:** Similarity matrix based on bone contacts.

	*Desmatochelys lowii*	*Eretmochelys imbricata*	*Dermochelys coriacea*	*Chelydra serpentina*
*Desmatochelys lowii*	–	44%	34%	33%
*Eretmochelys imbricata*	44%	–	51%	45%
*Dermochelys coriacea*	34%	51%	–	38%
*Chelydra serpentina*	33%	45%	38%	–

**Note:**

The similarity between taxa is shown in %.

### Phylogenetic analysis

**Figure 44:**
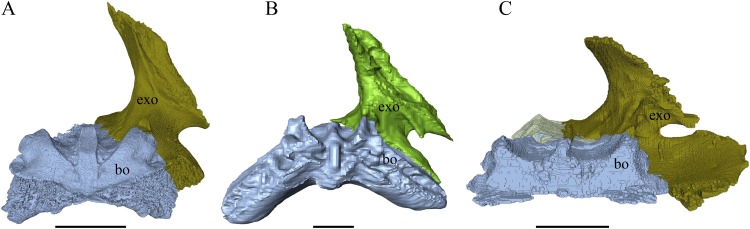
Basioccipital and exoccipital in anterior view. (A) *Eretmochelys imbricata*; (B) *Dermochelys coriacea*; and (C) *Chelydra serpentina*. The bar marks 10 mm.

The phylogenetic analysis resulted in 10 most parsimonius trees with 932 steps. A time calibrated extract of the strict consensus tree is provided in Fig. 44. Protostegidae is universally recovered as monophyletic outside of crown Chelonioidea.

## Discussion

### Morphology

Absence of the processus epipterygoideus of *D. lowii*

The processus epipterygoideus was defined by Gaffney (1972) to be an anterior extension of the quadrate that either ascends toward the epipterygoid or the descending process of the parietal and normally is situated ventral to the foramen nervi trigemini. This structure does not exist in *D. lowii*. Instead, the contact between the quadrate and the pterygoid is rather straight and no process-like structure can be observed on the anterior part of the quadrate. A lack of the processus epipterygoideus has previously only been noted in pleurodires (Gaffney, 1979), but the structure is rarely addressed in the description of fossils. It is therefore unclear to me if the absence of this structure in *Desmatochelys lowii* is an autapomorphy or has phylogenetic significance.

“Pineal foramen” of *D. lowii*

**Figure 45:**
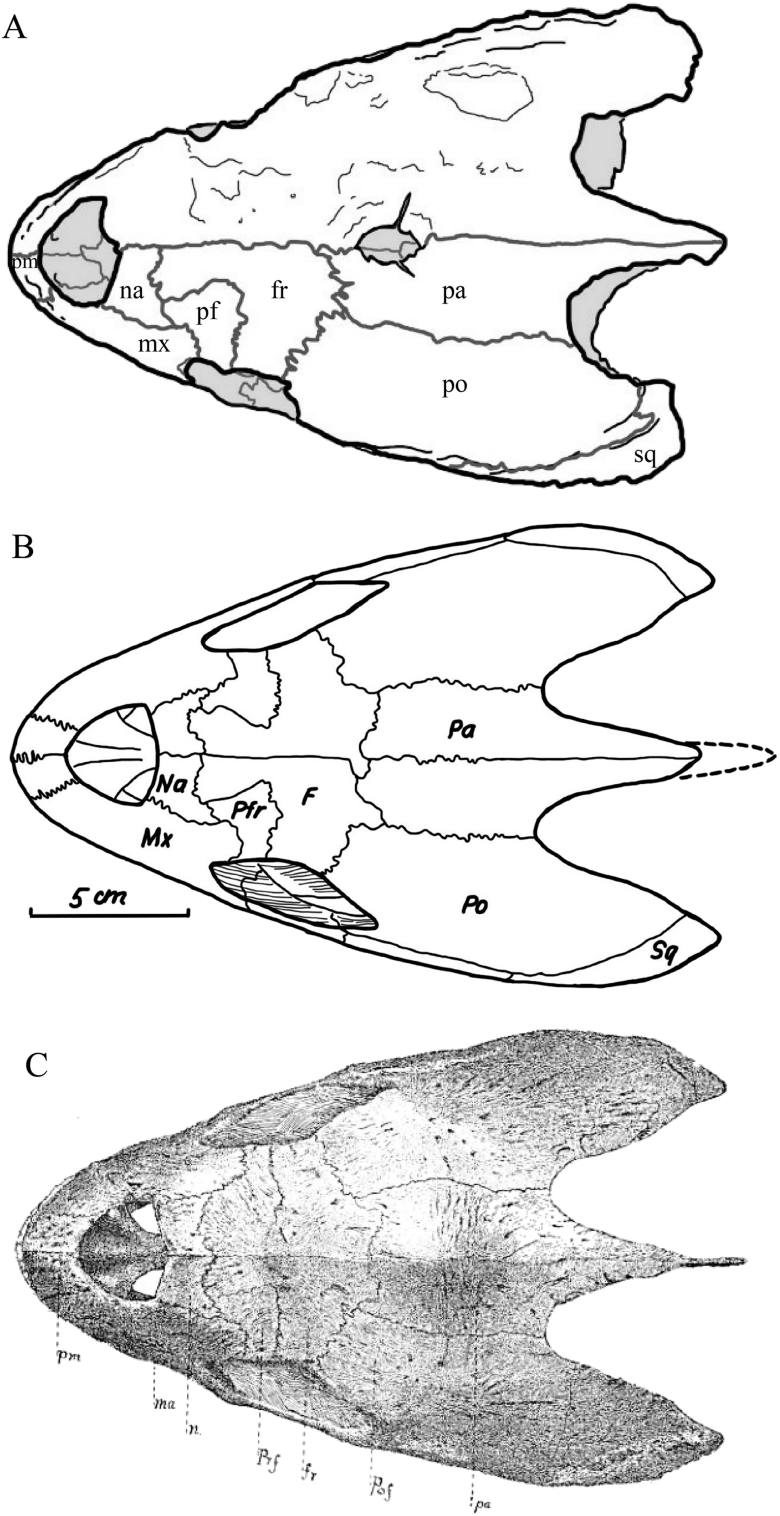
Comparison historical illustrations of KUVP 1200, holotype of *D. lowii*, in dorsal view. (A) Illustration of current condition of specimen; (B) redrawn from Zangerl & Sloan (1960); and (C) reproduced from Williston (1894).

The skull roof of KU VP1200, the holotype of *D. lowii*, is currently characterized by a median hole located between the parietals, just posterior to the frontals (Fig. 45). Elliot, Irby & Hutchison (1997) and Cadena & Parham (2015) interpreted this hole to be a pineal foramen, but I here conclude it to be an artifact, as neither Williston (1894, Fig. 45C) nor Zangerl & Sloan (1960, Figs. 45B and 46) illustrate or mention the presence of a pineal foramen in this specimen. The description of Williston (1894) is relatively brief, but he explicitly mentions damage to the basicranium, while illustrating an intact dorsal skull roof. Zangerl & Sloan (1960) similarly draw attention to the broken crista supraoccipitalis and the crushed basicranium of the skull, but once again do not highlight any damages to the skull roof. As all of these authors certainly were aware of the significance that a pineal foramen would have in a fossil turtle, it seems all but certain that the hole was not yet present when they studied this skull. Detailed observation of the margins surrounding the hole reveals radiating fractures, which further suggests that this part of the skull was damaged postburial, likely in the last decades. It is nevertheless clear that the parietals are unusually thin in the damaged part of the skull.

**Figure 46:**
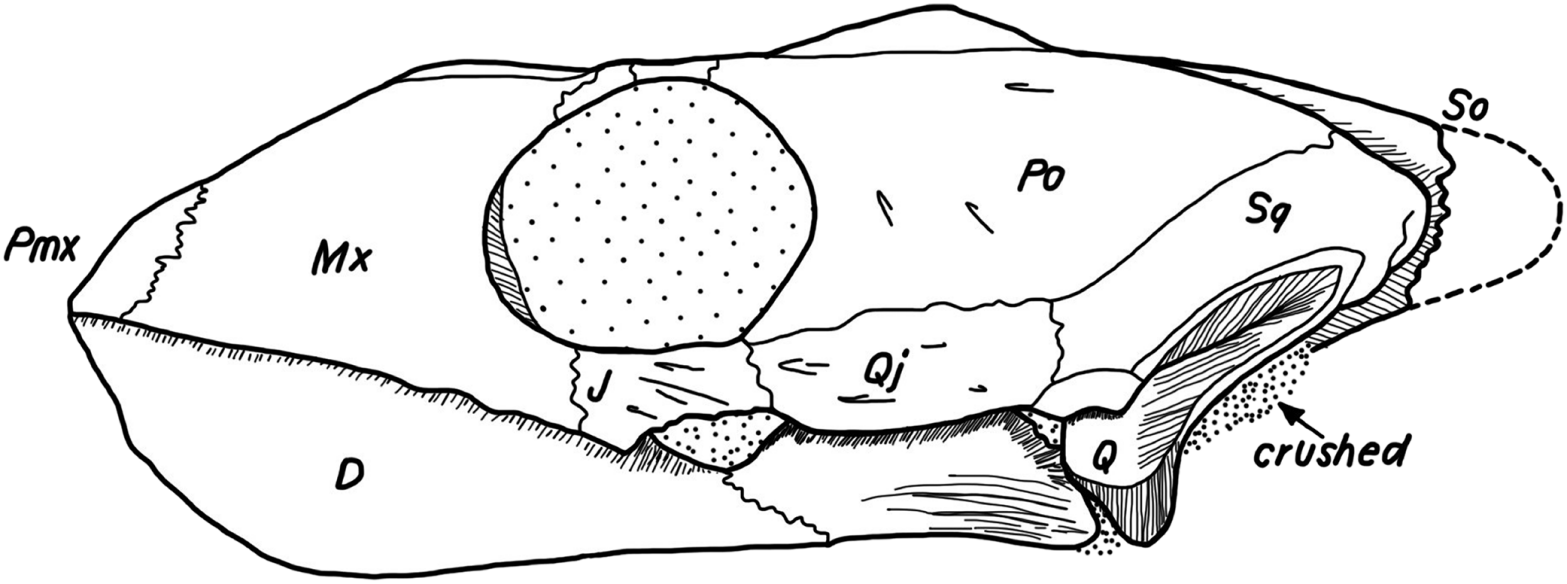
Illustration of KUVP 1200, holotype of *D. lowii*, in lateral view, redrawn from Zangerl & Sloan (1960). Note that crushed basicranium and broken tip of the supraoccipital are marked, but no damage indicated to the dorsal bulge.

**Figure 47:**
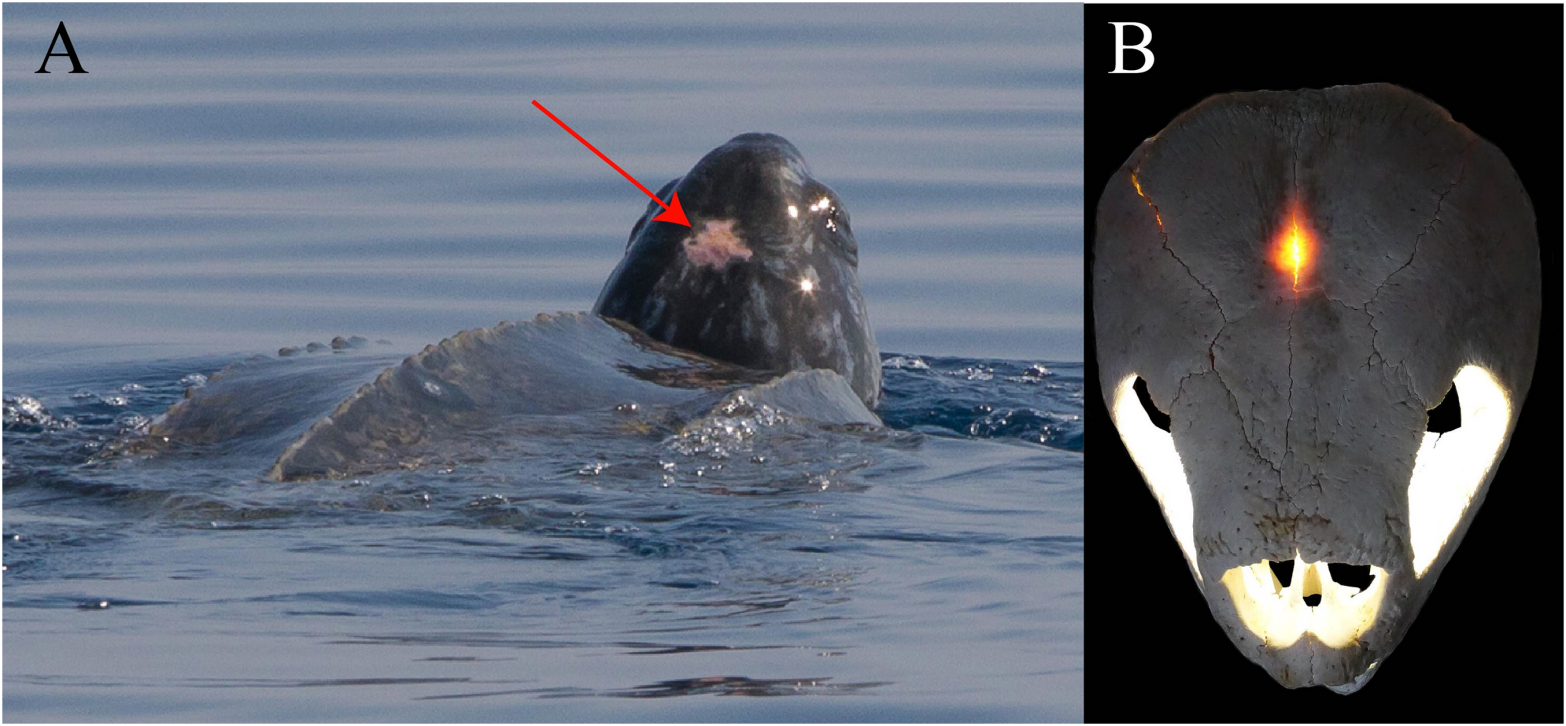
Pictures of the “pink spot” of *D. coriacea*. (A) “Pink spot” marked with a red arrow on a living *D. coriacea* (picture by Silvia Bonizzoni, Dolphin Biology and Conservation) and (B) skull of a *D. coriacea* with light shining from the interior through the thinnest part of the parietal’s dorsal plate.

The hole in the skull of *D. lowii* is located on top and slightly anterior to an elevation on top of the skull roof, just above the highest point of the braincase, as can be observed in the medial view of the 3D models. In *D. coriacea* a similar arrangement can be observed, but the parietal bone is only thinned instead of showing a hole. Davenport et al. (2014) conducted a study on the so called “pink spot” on the head of *D. coriacea*, an unpigmented spot in living individuals located just above the thin bone roofing the top of the braincase. At the uppermost part of the brain, the pineal gland (epiphysis) is situated (Wyneken, 2001). Davenport et al. (2014) suggested that this translucent spot (Fig. 47) helps stimulate the pineal gland by allowing light to pass through the skull roof as a way to sense seasons. This condition of the skull of *D. lowii* appears to be equivalent to that of *D. coriacea* and it seems reasonable to infer that it may have served a similar function. Only a sister group relationship, however, will be able to confirm if these structures are truly homologous (i.e., synapomorphic).

Basicranium of *D. lowii*

Unfortunately, it is nearly impossible to discern characters from the basicranium of the available specimen of *Desmatochelys lowii* that may yield phylogenetically relevant information. The basicranium is badly crushed, partially missing, and the contrast between the bone and the matrix is low. As a result, it is nearly impossible to observe any sutures, even in the scans. In addition to that, in the area of the posterior part of the basisphenoid and the anterior part of the basioccipital, a hole of one cm diameter was drilled for public installation. Neither Williston (1894) nor Zangerl & Sloan (1960) illustrate the posteroventral part of this specimen, but instead note its poor preservation.

Everhart & Pearson (2009) recently reported a fossil marine turtle (FHSM VP17470), which they preliminarily identified as *D. lowii*. The fossil consists of a fragmented shell, limb bones, paddles, and a skull from the Turonian Fairport Chalk of Mitchell County, Kansas, USA. The skull is crushed, but the basicranium seems to be intact. A detailed analysis of this specimen will first need to clarify if it is indeed referable to *D. lowii* and I therefore here refrain from describe the morphology of its basicranium.

### Parasphenoid

The parasphenoid is a dermal bone that is located below the endochondral basisphenoid and that occurs in most vertebrates (De Beer, 1937; Pehrson, 1945; Jollie, 1957; Bellairs & Kamal, 1981; Bratislava, 1993; Rieppel, 1993; Sheil, 2013). However, the two bones often fuse during ontogeny, making it difficult to distinguish them from one another in adult specimens (Sterli et al., 2010). Pehrson (1945) conducted an embryological study and summarized the available literature on the development and presence of the parasphenoid in reptiles and concluded that cheloniids do not posses a parasphenoid, as the corresponding blastemas do not ossify during ontogeny of *Lepidochelys olivacea*. By contrast, all other turtles, including *D. coriacea*, at some point of their ontogeny shows signs of an ossified parasphenoid, a conclusion recently confirmed by observations from fossil turtles (Sterli et al., 2010; Rabi et al., 2013).

**Figure 48:**
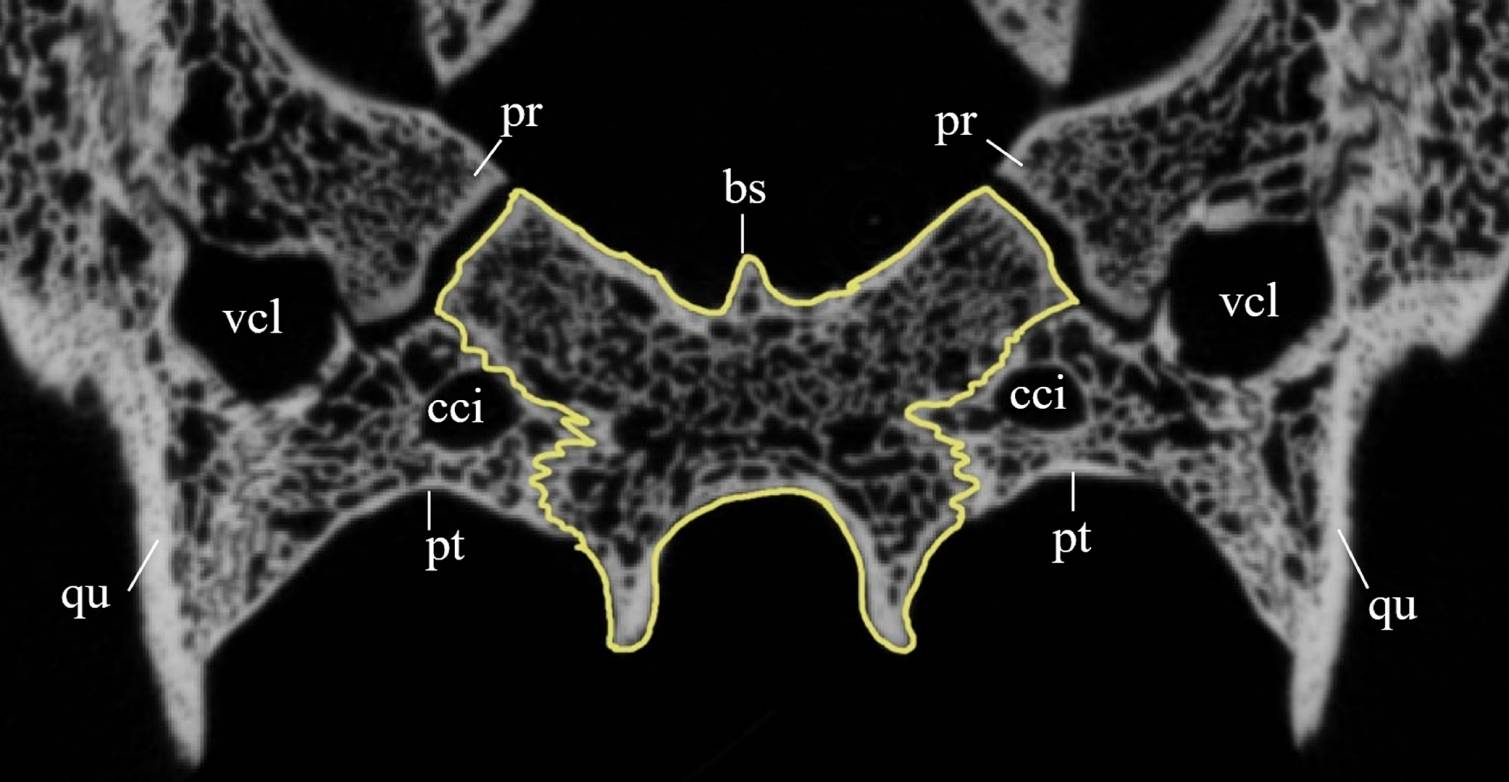
Cross section image of the basisphenoid of *Eretmochelys imbricata*. The yellow line marks the outline of the basisphenoid.

As it is difficult to discern the parasphenoid in extant turtles externally, the use of CT scans provides novel access to this structure. The available adult specimen of *C. serpentina* allows discerning the parasphenoid from the basisphenoid toward the anterior, but both bones become indistinguishable toward the posterior. On the other hand, the parasphenoid can be fully distinguished from the basisphenoid in the available adult specimen of *D. coriacea*. Although high quality scans are available for *E. imbricata*, there is no trace of a distinct, dense bone that underlies the fully spongiose basisphenoid therefore confirming the absence of this structure in this species of marine turtle (Fig. 48). Finally, the parasphenoid could also not be observed in the available specimen of *D. lowii*, but as internal structures cannot be resolved with any confidence in the basicranium of this specimen, this should not be taken as evidence of absence.

Sulcus pro-epipterygoidei in *C. serpentina*

**Figure 49:**
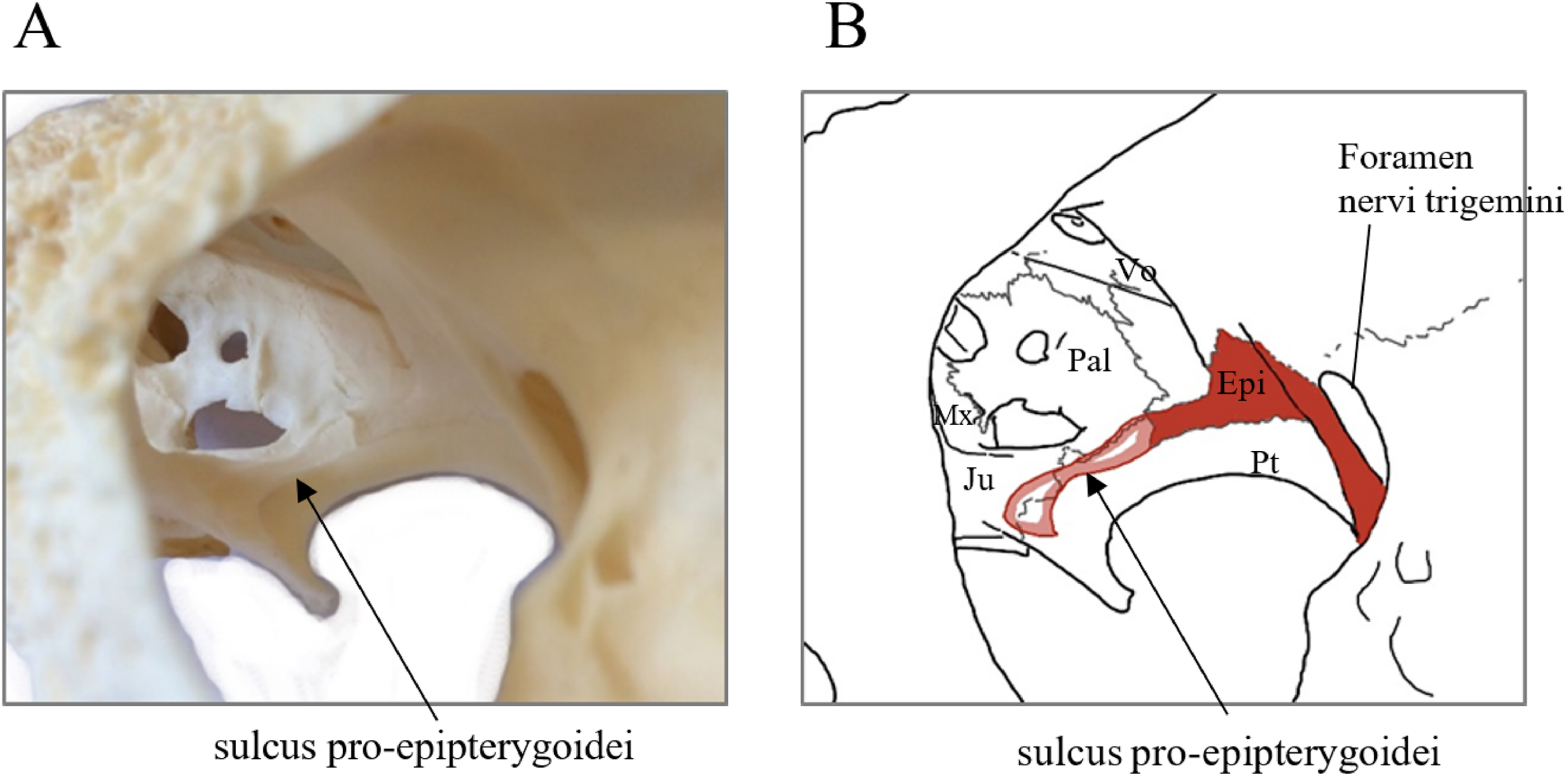
Photography (A) and illustration (B) of the sulcus pro-epipterygoidei. In the illustration, the dark red area marks the epipterygoid, while the anterior to it light red area highlights the extent of the sulcus epipterygoidei.

In *C. serpentina* a groove is present on the dorsal side of the external pterygoid process (Fig. 49). Gaffney (1972) illustrated and briefly noted (Gaffney, 1979) that there is an unnamed “anterior space” that holds the unossified anterior extension of the anterior process of the epipterygoid. This extension is ossified, among others, in various emydids (Gaffney & Meylan, 1988; Joyce & Bell, 2004). To aid communication, I propose the term *sulcus pro-epipterygoidei*, which highlights the anatomical position of this groove in the anterior prolongation of the epipterygoid. As this structure has not yet been reported for many turtles, its phylogenetic relevance is unclear to me.

### Phenetic analysis of bone contacts

The use of CT scanning technology not only provides novel insights into the internal morphology of the skull (i.e., brain endocast, nerve and circulation canal systems), but also the nature of the bony contacts. I here document the detailed nature of all available bony contacts for four turtle taxa in the form of tables (Tables S3.1–S3.4). Although the information encoded in these tables may contain phylogenetic information, it is impractical to integrate it into an explicit phylogenetic matrix for the moment, as only four taxa would be scored for all newly developed characters.

As an alternative I here present a phenetic study that quantifies the amount of similarity in regards to the contacts developed between two taxa. The results of this study (Table 4) show that *E. imbricata* and *D. coriacea* show the greatest amount of bone contact similarity with 51%. *C. serpentina* shares more similar bone contacts with *E. imbricata* (45%) than with *D. coriacea* (38%). *Desmatochelys lowii* has more similarities in bone contacts with *E. imbricata* (44%) than with *Dermochelys coriacea* (35%) and the fewest with *C. serpentina* (33%). Among extant species, the strong similarity between *E. imbricata* and *D. coriacea* and their lower degree of similarity with *C. serpentina* correspond with the currently hypothesized phylogenetic relations of these species (Crawford et al., 2015). If similarity is used as a tentative phylogenetic tool, the high dissimilarity between *D. lowii* and all extant taxa might be used to suggest that it is perhaps situated outside the clade formed by the extant taxa (i.e., Americhelydia). This result is consistent with protostegids not being situated within or near Chelonioidea. Nevertheless, it has to be mentioned that there is a certain bias in the data of *D. lowii* due to the fact that some contacts are obscured by matrix and not completely identifiable. It would be interesting to further test this method by exploring if additional recent turtle species are “correctly” placed in currently accepted phylogenies as well, as this data might contribute to the independent test of phylogenetic relationships with using morphology.

### Phylogeny

As part of this study, I expanded the phylogenetic analysis of Cadena & Parham (2015) by updating the scoring of *D. lowii* based on the new observations obtained herein and by adding seven new characters. Two analyses were performed that differ in the inclusion of the new characters. The cladogram that only utilizes the updated coding of *Desmatochelys lowii* does not show any major differences to the one in Cadena & Parham (2015) by recognizing a monophyletic Protostegidae within Dermochelyidae. The cladogram that includes the new character as well, however, places Protostegidae outside of crown Chelonioidea. The resulting topology (see Fig. 44) has weak resolution within Protostegidae. Only *Archelon ischyros* and *Protostega gigas* are forming a clade, but not *D. padillai* and *D. lowii*. There is no reason to name a new genus for *padillai* for the moment, as the cladogram is too poorly resolved to contradict the sister group relationship previously hypothesized for the two currently accepted species of *Desmatochelys* (Cadena & Parham, 2015). The analyses highlight that the improved scoring of *D. lowii* had no impact on the topology, in contrast to the newly developed characters.

Placement of protostegids along the stem lineage of Chelonioidea is a novel result for a global phylogenetic analysis of turtle relationships. Joyce (2007) had shown “protostegids” to be situated outside crown Cryptodira, but sampling was limited to *S. gaffneyi*. Using a large sample of marine turtles in a global context, Cadena & Parham (2015) on the other side recently retrieved Protostegidae within Dermochelyidae. This result mirrors previous analyses of marine turtle relationships (Hirayama, 1998; Kear & Lee, 2006), but is contradicted by molecular calibration analyses, that suggest a divergence data for crown Chelonioidea near the K–T boundary, not the Barremian, as suggested by the oldest known protostegids (Joyce et al., 2013). The herein proposed placement of protostegids partially resolves this conflict, as Barremian protostegids are still within the maximum proposed divergence date for Americhelyidia, the next more inclusive clade (Joyce et al., 2013).

## Conclusions

The type skull of *D. lowii* from the late Cretaceous (middle Cenomanian to early Turonian) Greenhorn Limestone of Jefferson County, Nebraska, is herein redescribed in detail using μCT scans to provide new data that may help resolve the conundrum surrounding the phylogenetic placement of the Cretaceous marine turtle group Protostegidae and the origin of extant marine turtles. The detailed external and internal morphology of this specimen are compared bone by bone with the extant marine turtles *D. coriacea* and *E. imbricata* and the snapping turtle *C. serpentina*. Novel insights include the realization that the pineal gland may have approached the surface of the skull of *D. lowii*, but that a true foramen is not developed, as in the extant *D. coriacea*. A parasphenoid, or at least remnants of the parasphenoid, are present in *C. serpentina* and *D. coriacea*, but confirmed to be absent in *E. imbricata*. The available skull of *D. lowii* is too poorly preserved to allow discerning the presence of this bone. A sulcus is found in the anterior prolongation of the epipterygoid in *C. serpentina*, which is herein named the sulcus pro-epipterygoidei. Protostegids should be checked for the processus epipterygoideus, the recess on the parietal, and the parasphenoid. A phenetic analysis that utilizes newly obtained bone contact data suggests that *Desmatochelys lowii* is least similar of the four turtles included, which would be concordant with a phylogenetic placement outside Americhelydia. The recent global phylogeny of turtle relationships of Cadena & Parham (2015) was expanded through the inclusion of seven new characters and updated in regards to the coding of *D. lowii*. The resulting phylogenetic analysis is in broad agreement with that of Cadena & Parham (2015), but inclusion of the new characters results in the placement of Protostegidae outside of Chelonioidea. Additional insights into the basicranial anatomy of *D. lowii* might be gained in the future by obtained μCT scans of FHSM VP17470 and MNA V4516, two crushed skulls from the Late Cretaceous of Kansas and Arizona, respectively, which have preliminarily been referred to that taxon, but still lack detailed description (Elliot, Irby & Hutchison, 1997; Everhart & Pearson, 2009). As the phenetic study herein obtained results that are broadly congruent with some recent phylogenetic analyses, it may be of interst to expand the dataset through the addition of more taxa.

## Supplemental Information

10.7717/peerj.5964/supp-1 Supplemental Information 1 Matrix.

10.7717/peerj.5964/supp-2 Supplemental Information 2 Supplementary material.

## Institutional Abbreviations

KU: University of Kansas, Lawence, Kansas, USA
FHSM: Fort Hays State Musuem, Fort Hays, Kansas, USA
MNA: Museum of Northern Arizona, Flagstaff, Arizona, USA
NMB: Naturhistorisches Museum Basel, Basel, Switzerland
UFR: Université de Fribourg, Fribourg, Switzerland
SMF: Senkenberg Naturmuseum, Frankfurt, Germany.

## Abbreviations Used in Figures

bo: basioccipital
bs: basisphenoid
b. col: basis collumelae
ccc: canalis caroticus cerebralis
col: columella
epi: epipterygoid
exo: exoccipital
f: foramen
fna: foramen nervi abducentis
fr: frontal
ju: jugal
mx: maxilla
na: nasal
op: opisthotic
pa: parietal
pal: palatine
pc: processus clinoideus
pf: prefrontal
pm: premaxilla
po: postorbital
pr: prootic
pt: pterygoid
qj: quadratojugal
qu: quadrate
so: supraoccipital
st: sella turcica
tb: tuberculum basioccipitale
vo: vomer

## Additional Information and Declarations

### Data Availability

The following information was supplied regarding data availability: The CT scan images of the four examined specimens are available at Morphobank. *Dermochelys coriacea* http://morphobank.org/permalink/?P3142; *Chelydra serpentina* http://morphobank.org/permalink/?P3143; *Eretmochelys imbricata* http://morphobank.org/permalink/?P3145; *Desmatochelys lowii* http://morphobank.org/permalink/?P3146.
