# Supplementary material for: Comparative cranial morphology of the Late Cretaceous protostegid sea turtle Desmatochelys lowii
Source: PeerJ. 2018 Dec 7;6:e5964. doi: 10.7717/peerj.5964 (PMC6287587; doi:10.7717/peerj.5964)
Supplement: Supplemental Information 2 [file peerj-06-5964-s002.pdf]

SUPPLEMENTARY MATERIAL

Appendix S1: Volume Renderings and CT Scan Slice Examples of the Specimens

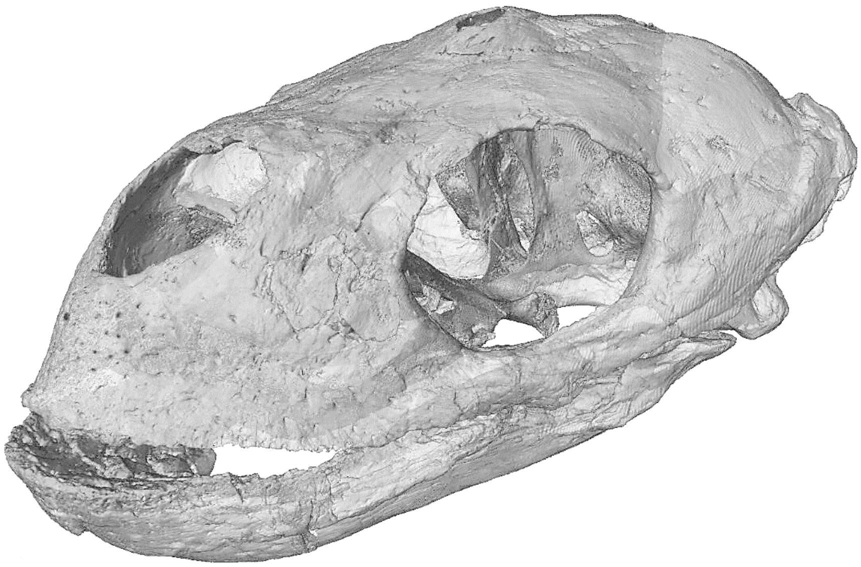

Figure S1.1: Volume rendering of *Desmatochelys lowii*

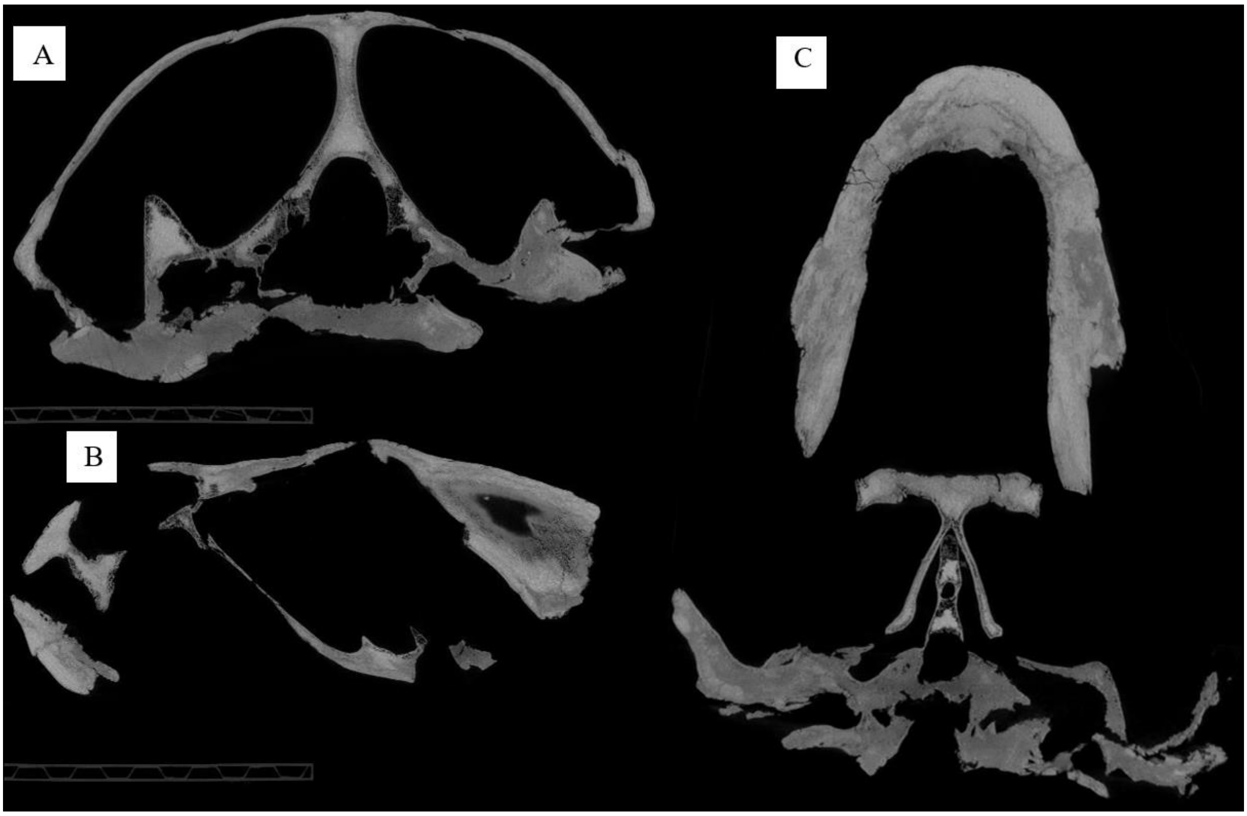

Figure S1.2: Cross-section images of *Desmatochelys lowii*. A: coronal plane, B: saggital plane C: transverse plane.

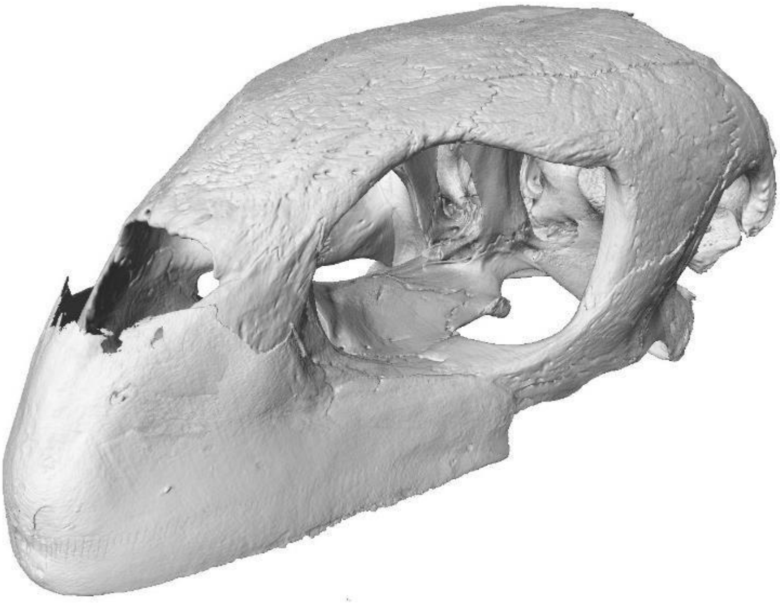

Figure S1.3: Volume rendering of the *Eretmochelys imbricata.*

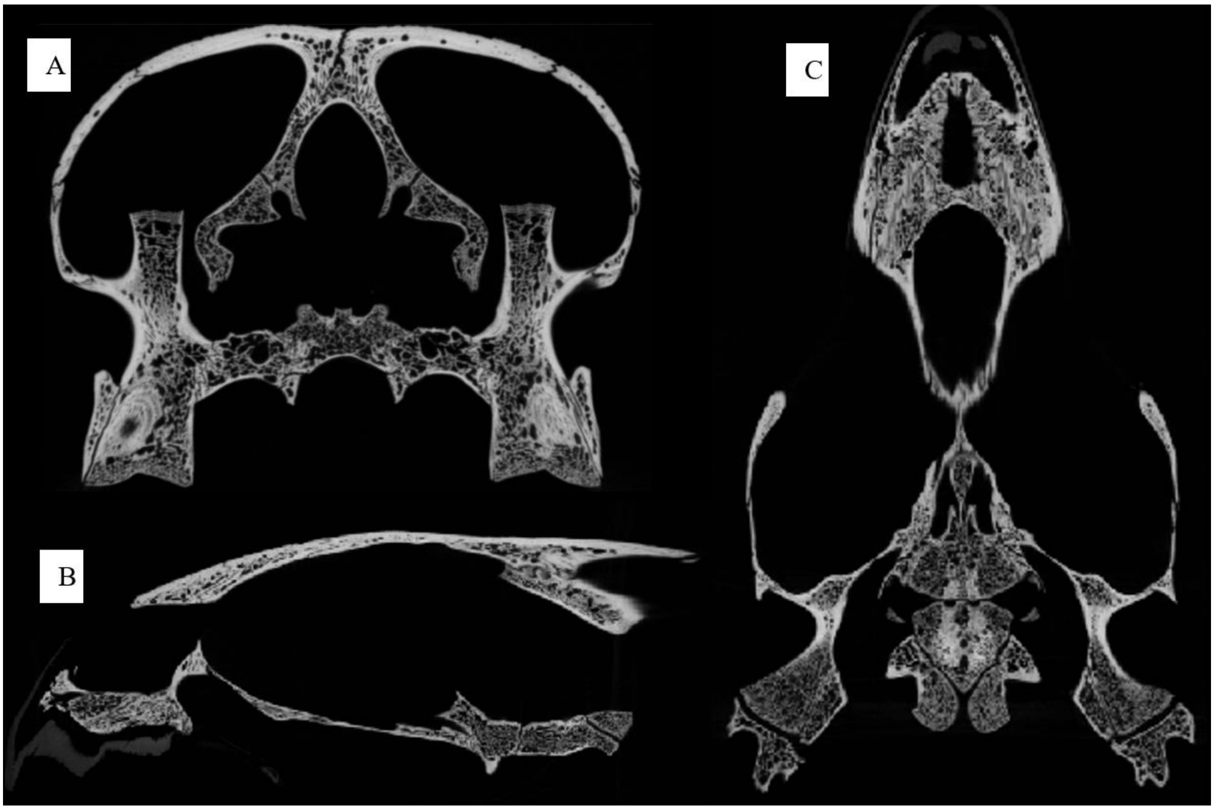

Figure S1.4: Cross-section images of *Eretmochelys imbricata.* A: coronal plane, B: saggital plane C: transverse plane.

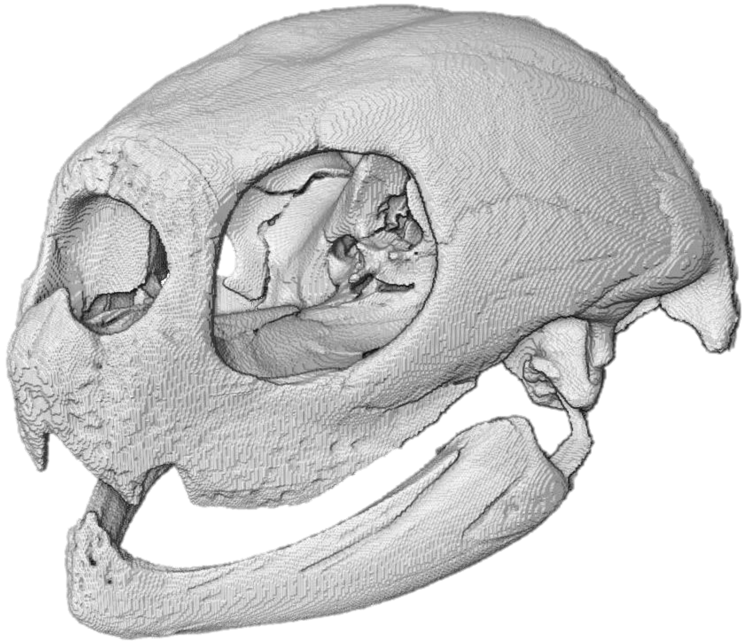

Figure S1.5: Volume rendering of *Dermochelys coriacea.*

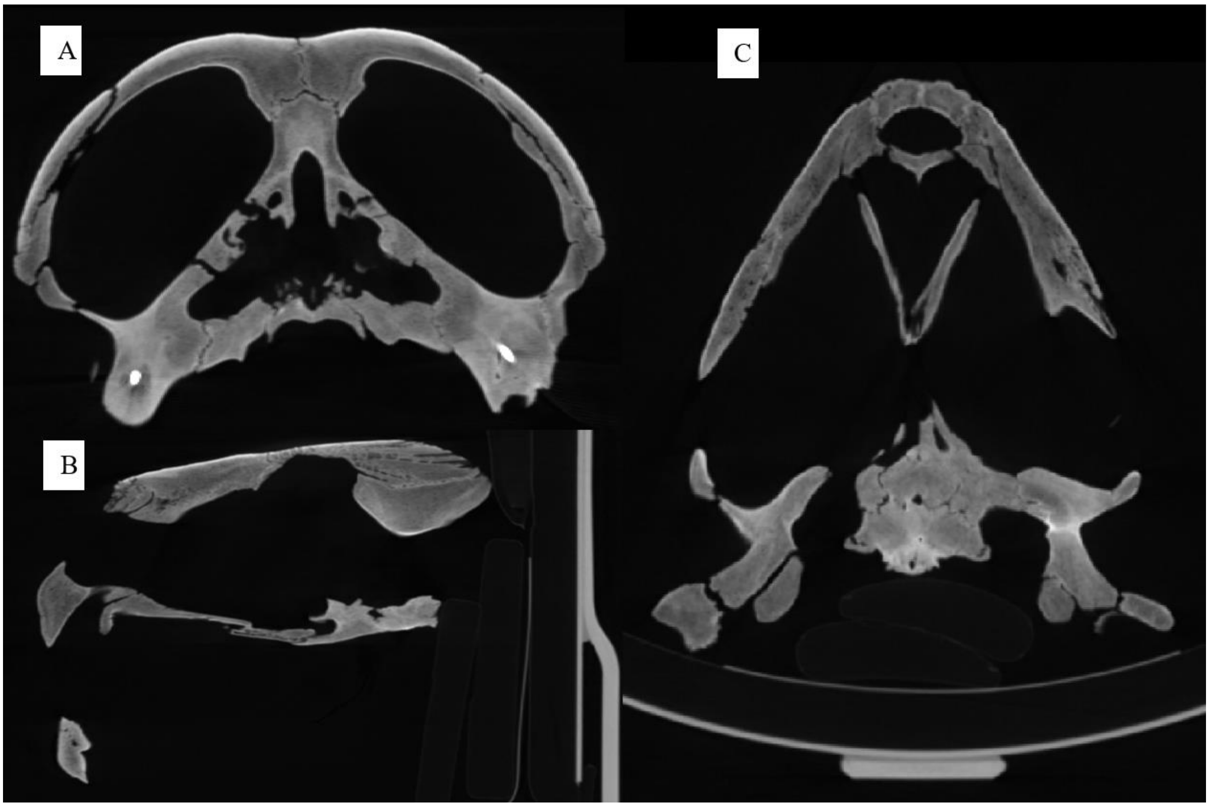

Figure S1.6: Cross-section images of *Dermochelys coriacea.* A: coronal plane, B: saggital plane C: transverse plane.

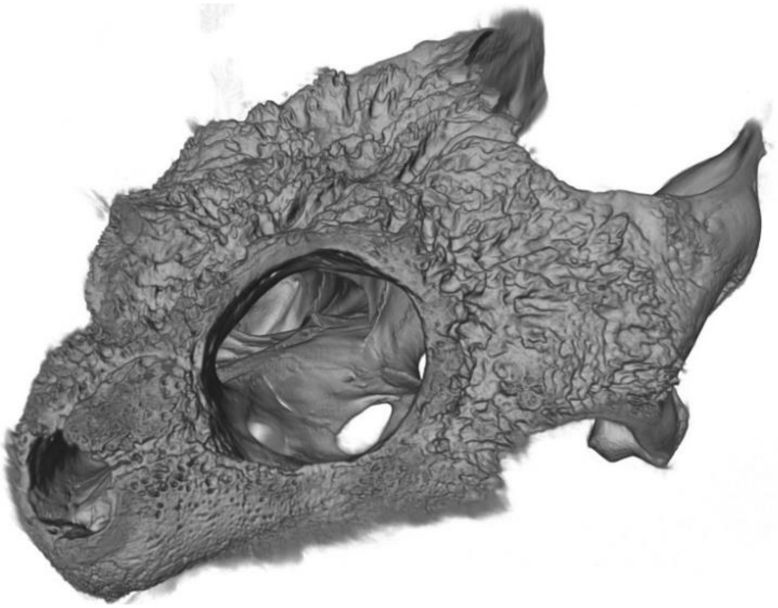

Figure S1.7: Volume rendering of *Chelydra serpentina.*

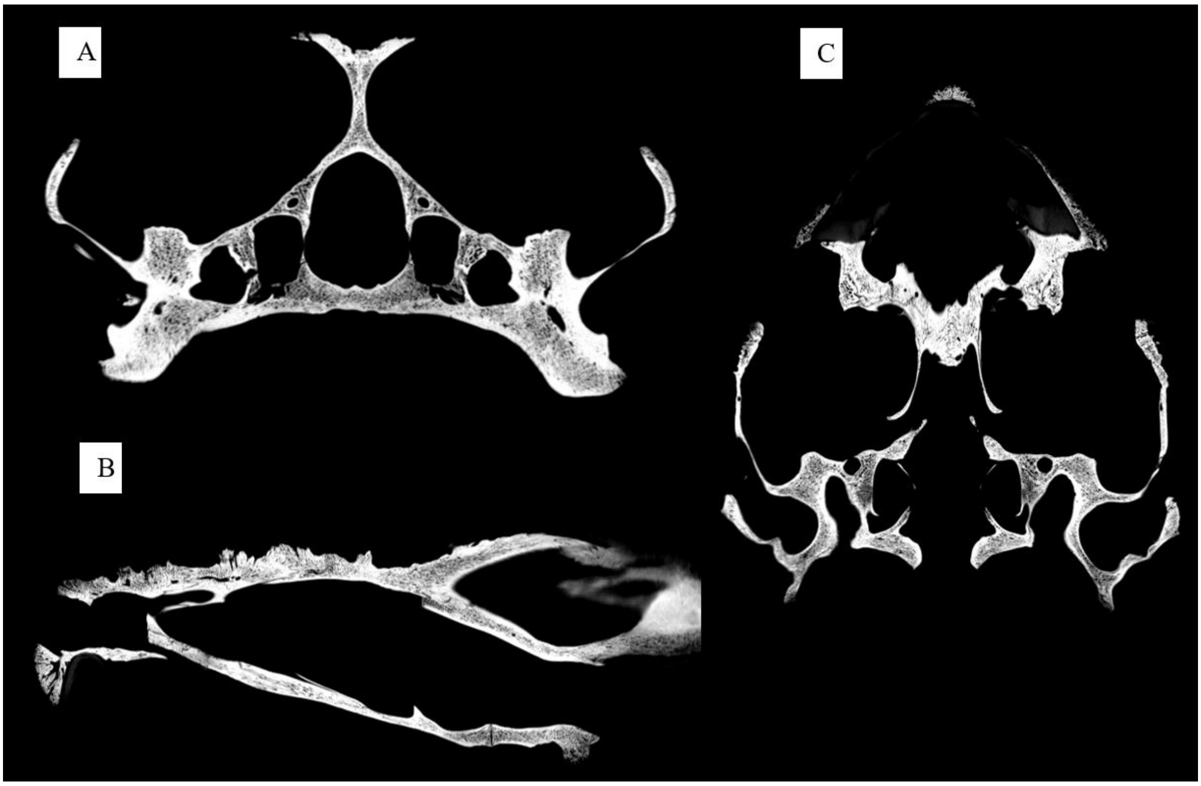

Figure S1.8: Cross-section images of *Chelydra serpentina.* A: coronal plane B: saggital plane C: transverse plane.

Appendix S2: List of Segmented Bones

Out of four specimens, in total 81 skull bones were segmented. Every bone was segmented only once per specimen, although some bones occur twice in a skull, e.g. the parietals.

Table S2.1: Segmented bones listed for each specimen. 0 = absent, 1 = present, ? = determination unclear.

	ossified elements	*Desmatochelys lowii*	*Eretmochelys imbricata*	*Dermochelys coriacea*	*Chelydra serpentina*
dermal roofing	Nasal	1	0	0	0
	Prefrontal	1	1	1	1
	Frontal	1	1	1	1
	Parietal	1	1	1	1
	Poostorbital	1	1	1	1
	Jugal	1	1	1	1
	Quadratojugal	1	1	1	1
	Squamosal	1	1	1	1
palate	Premaxilla	1	1	1	1
	Maxilla	1	1	1	1
	Vomer	1	1	1	1
	Palatine	1	1	1	1
basicranium & braincase	Pterygoid	1	1	1	1
	Epipterygoid	1	?/1	0	1
	Basisphenoid	1/?	1	1	1
	Parasphenoid	?	0	1	?/1
	Prootic	1	1	1	1
	Opistotic	1	1	1	1
	Quadrate	1	1	1	1
	Basioccipital	1/?	1	1	1
	Exoccipital	1	1	1	1
	Supraoccipital	1	1	1	1

Appendix S3: Phenetic Analysis-Tables

Table S3.1: Bone contacts in *Desmatochelys lowii.* p: parallel; o: overlapping; u: underlying; c: clasping; cb: clasped by; t: vertically transverse; s: smooth; if: faintly interfingering; im: moderately interfingering; ?: determination unclear

A \ B	Na	Pf	Fr	Pa	Po	Ju	Qj	Sq	Pm	Mx	Vo	Pal	Pt	Epi	Bs	Ps	Pr	Op	Qu	Bo	Exo	So
Nasal	p+if	o	u							o+is												
Prefrontal	u		o+im							u+is, c	u+s,o+ is,p+i	u?										
Frontal	o	u+im	p+im	o+im	u+im																	
Parietal			u+im	p+im	u+if								t+is	t			t+im					o
Postorbital			o+im	o+if		c	o+is	o+s,cb ,u+s,c														
Jugal					cb		o			o+im												
Quadratojugal					u+is	u		o											s			
Squamosal					u+s,c, o+s,c		u											p	u+if,c			
Premaxilla									p+im	p	p?											
Maxilla	u+is	o+is, cb				u+im			p		u?cb?	cb, o										
Vomer		o+s,u+ is,p+i							p?	o?c?	-	u?+i										
Palatine		o?								c, u	o?+i	p+im	u+i									
Pterygoid				t+is								o+i	p+i	t	?		?	?	c+if	?		
Epipterygoid				t									t									
Basisphenoid													?		-					?	?	
Parashpenoid																-						
Prootic				t+im									?					o	p+if			t,p
Opisthotic								p					?				u		p,u,p		p	p
Quadrate							s	o+if,cb					cb+if				p+if	p,o,p				
Basioccipital													?		?					-	?	
Exoccipital															?			p		?		?
Supraoccipital				u													t,p	p			?	-

Tab. S3.2: Bone contacts in *Eretmochelys imbricata*. p: parallel; o: overlapping; u: underlying; c: clasping; cb: clasped by; t: vertically transverse; s: smooth; if: faintly interfingering; im: moderately interfingering; ?: determination unclear.

A \ B	Na	Pf	Fr	Pa	Po	Ju	Qj	Sq	Pm	Mx	Vo	Pal	Pt	Epi	Bs	Ps	Pr	Op	Qu	Bo	Exo	So
Nasal																						
Prefrontal		p+im	o+im							t+is	p+is											
Frontal		u+im	p+im	o+im, cb+is	u+im																	
Parietal			u+im, c+is	p+im	p+im			p+is					t+is									o+s, o+is
Postorbital			o+im	p+im		t+im	p+is	p+is														
Jugal					t+im		o+is			o+im		c+s										
Quadratojugal					p+is	u+is		t+im											p+im			
Squamosal				p+is	p+is		t+im											c+if	p+im			
Premaxilla									p+im	o+im	o+is											
Maxilla		t+is				u+im			u+im		p+im, o+im	o+im, cb+im, p+im										
Vomer		p+is							u+is	p+im, u+im	-	o+is, s, u+if	o+im									
Palatine						cb+s				u+im, c+im, p+im	u+is, s, o+if		o+im									
Pterygoid				t+is							u+im	u+im	p+im		u+s, u+im, cb+im		p+is, c+s		u+im	cb+im, o+im	u+is	
Epipterygoid																						
Basisphenoid													o+s, o+im, c+im		-		(p+s)			p+s,u+is		
Parashpenoid																-						
Prootic													(p+is, cb+s)		(p+s)			im	p+im, cb+im			t+im
Opisthotic								cb+if									im		u+if		o+s+im	
Quadrate							p+im	p+im					o+im				p+im, c+im	o+if				
Basioccipital													c+im, u+im		p+s,o+is					-	u+im	
Exoccipital													o+is					u+s+im		o+im		t+is,cb
Supraoccipital				u+s, u+is													t+im				t+is, c	-

Tab. S3.3: Bone contacts in *Dermochelys coriacea*. p: parallel; o: overlapping; u: underlying; c: clasping; cb: clasped by; t: vertically transverse; s: smooth; if: faintly interfingering; im: moderately interfingering; ?: determination unclear.

A \ B	Na	Pf	Fr	Pa	Po	Ju	Qj	Sq	Pm	Mx	Vo	Pal	Pt	Epi	Bs	Ps	Pr	Op	Qu	Bo	Exo	So
Nasal																						
Prefrontal		p+if	cb+if		o+is					t+if	t+s											
Frontal		c+if	p+im	cb+if	c+if																	
Parietal			c+if	p+im	u+im			c,u+s														o+s, o+if
Postorbital		u+is	cb+if	o+im		c+if	t+s	o+if														
Jugal					cb+if		o+if	t+s		o+is												
Quadratojugal					t+s	u+if		cb+if											o+im, p+if			
Squamosal				cb,o+s	u+if	t+s	c+if												o+if, p+im, cb			
Premaxilla									p+if	p+if	o+s											
Maxilla		t+if				u+is			p+if			p+im										
Vomer		t+s							u+s		-	p+if, o+s	o+s									
Palatine										p+im	p+if, u+s		o+s, u+s									
Pterygoid											u+s	u+s, o+s	p+is		p+is	o+if	t+if		u+im	cb+im		
Epipterygoid																						
Basisphenoid													p+is		-	p+if	t+s			p+im		
Parashpenoid													u+if		p+if	-				u+if		
Prootic													t+if		t+s			p+im	p+im			p+if
Opisthotic																	p+im		cb+if		o+if	p+if
Quadrate							u+im, p+if	u+if, p+im,c					o+im				p+im	c+if				
Basioccipital													c+im		p+im	o+if				-	u+im	
Exoccipital																		u+if		o+im		u+im
Supraoccipital				u+s, u+if													p+if	p+if			o+im	-

Tab. S3.4: Bone contacts in *Chelydra serpentina*. p: parallel; o: overlapping; u: underlying; c: clasping; cb: clasped by; t: vertically transverse; s: smooth; if: faintly interfingering; im: moderately interfingering; ?: determination unclear.

A \ B	Na	Pf	Fr	Pa	Po	Ju	Qj	Sq	Pm	Mx	Vo	Pal	Pt	Epi	Bs	Ps	Pr	Op	Qu	Bo	Exo	So
Nasal																						
Prefrontal		p+im	c+im, p+im		o+if					t+is, p+im	c+is	o+im										
Frontal		cb+im, p+im	p+if	o+is, p+im	p+im																	
Parietal			u+is, p+im	p+if	o+im									t+s			o+if, c+s					o+im
Postorbital		u+if	p+im	u+im		t+if, p+im	p+im	p+im														
Jugal					t+if, p+im		p+if			o+im		p+s	cb+is									
Quadratojugal					p+im	p+if		c+if											p+im			
Squamosal					p+im		cb+if											p+im	c+if			
Premaxilla									p+im	p+im	cb+im											
Maxilla		t+is, p+im				u+im			p+im		o+if	cb+if	cb+im									
Vomer		cb+is							c+im	u+if		u+if	cb+im									
Palatine		u+im				p+s				c+if	o+if		cb+im									
Pterygoid						c+is				c+im	c+im	c+im	p+is	u+im, t+s	u+s, p+im	o+s	u+if, cb+s		u+if	cb+if	u+im	
Epipterygoid				t+s									o+im, t+s									
Basisphenoid													o+s, p+im		-	p+if	p+im, p+s			p+s, p+im		
Parashpenoid													u+s		p+if	-						
Prootic				u+if, cb+s									o+if, c+s		p+im, p+s			p+s	p+im			o+im, p+s
Opisthotic								p+im									p+s		c+is	(p+s)	o+is	p+s
Quadrate							p+im	cb+if					o+if				p+im	cb+is				
Basioccipital													c+if		p+s, p+im			(p+s)		-	?	
Exoccipital													o+im					u+is		?	?	u+im, cb
Supraoccipital				u+im													u+im, p+s	p+s			o+im, c	-

Appendix S4: Backbone Constraint Tree Topology

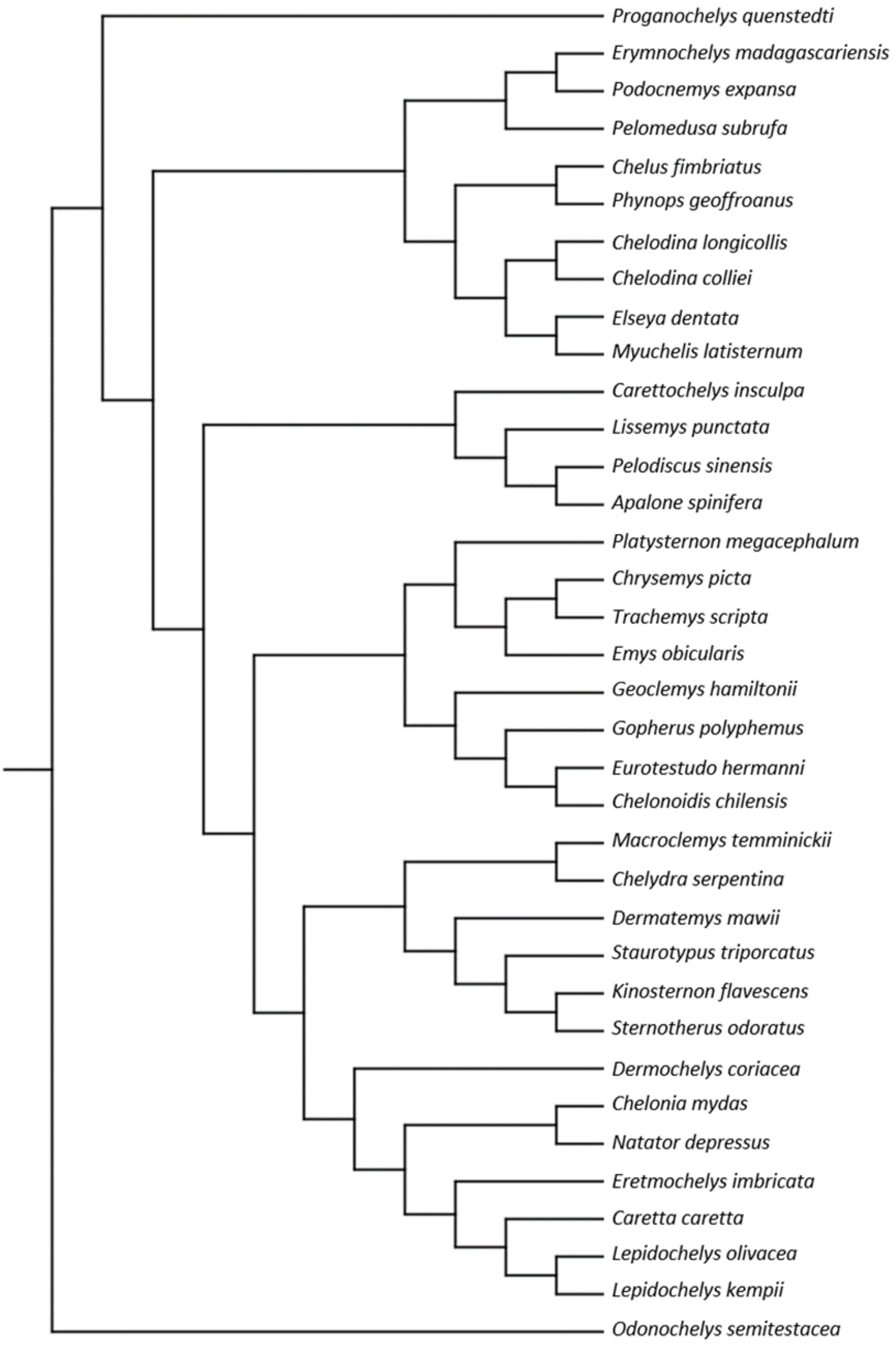

Figure S4.1: Backbone constraint tree topology after Crawford et al. 2015

Appendix S5: Inactive Taxa

Inactive taxa (81/154) from the initial matrix of Cadena & Parham (2015)

1	*Annemys latiens*	28	*Chisternon undatum*	55	*Niolamia argentina*
2	*Basilochelys macrobios*	29	*Chitracephalus dumonii*	56	*Notoemys laticentralis*
3	*Changmachelys bohlini*	30	*Condorchelys antiqua*	57	*Notoemys oxfordiensis*
4	*Eileanchelys waldmani*	31	*Corsochelys haliniches*	58	*Notoemys zapatocaensis*
5	*Chubutemys copelloi*	32	*Ctenochelys stenoporus*	59	*Ocepechelon bouyai*
6	*Liaochelys jianchangensis*	33	*Dinochelys whitei*	60	*Ordosemys skull*
7	*Manchurochelys*	34	*Dorsetochelys delairi*	61	*Patagoniaemys gasparinae*
8	*manchoukuoensis*	35	*Echmatemys wyomingensis*	62	*Peligrochelys walshae*
9	*Otwayemys cunicularius*	36	*Eochelone brabantica*	63	*Platychelys oberndorferi*
10	*Plastomenus aff. thomassii*	37	*Erquelinnesia gosseleti*	64	*Portlandemys macdowelli*
11	*Sinemys brevispinus*	38	*Euclastes acutirostris*	65	*Prochelidella cerrobarcinae*
12	*Stylemys nebrascensis*	39	*Euclastes platyops*	66	*Procolpochelys grandaeva*
13	*Xenochelys Formosa*	40	*Euclastes wielandi*	67	*Proterochersis robusta*
14	*Adocus amtgai*	41	*Heckerochelys romani*	68	*Protochelydra zangerli*
15	*Alienochelys selloumi*	42	*Helochelydra nopcsai*	69	*Rhinochelys pulchriceps*
16	*Allopleuron hoffmanni*	43	*Hoplochelys crassa*	70	*Sandownia harrisi*
17	*Angolachelys mbaxi*	44	*Hoyasemys jimenezi*	71	*Shachemys laosiana*
18	*Argillochelys africana*	45	*Indochelys spatulata*	72	*Siamochelys peninsularis*
19	*Argillochelys cuneiceps*	46	*Itilochelys rasstrigin*	73	*Sinemys gamera*
20	*Ashleychelys palmeri*	47	*Lophochelys*	74	*Syllomus aegyptiacus*
21	*Basilemys variolosa*	48	*Macroclemys schmidti*	75	*Tasbacka aldabergeni*
22	*Brachyopsemys tingitana*	49	*Mexichelys coahuilaensis*	76	*Tasbacka ouledabdounensis*
23	*Calcarichelys gemma*	50	*Mongolemys elegans*	77	*Terlinguachelys fischbecki*
24	*Carolinochelys wilsoni*	51	*Mongolochelys efremovi*	78	*Warkalania carinaminor*
25	*Chelonoidis gringorum*	52	*Neurankylus eximius*	79	*Xinjiangchelys junggarensis*
26	*Chelosphargis advena*	53	*Nichollsemys bareri*	80	*Yaminuechelys maior*
27	*Chengyuchelys*	54	*Ninjemys oweni*	81	*Yehguia ta*

82
